# Exposure to childhood maltreatment is associated with specific epigenetic patterns in sperm

**DOI:** 10.1038/s41380-024-02872-3

**Published:** 2025-01-03

**Authors:** Jetro J. Tuulari, Matthieu Bourgery, Jo Iversen, Thomas Gade Koefoed, Annukka Ahonen, Ammar Ahmedani, Eeva-Leena Kataja, Linnea Karlsson, Romain Barrès, Hasse Karlsson, Noora Kotaja

**Affiliations:** 1 FinnBrain Birth Cohort Study, Turku Brain and Mind Center, Department of Clinical Medicine, University of Turku, Turku, Finland; 2 Department of Psychiatry, Turku University Hospital and University of Turku, Turku, Finland; 3 Turku Collegium for Science and Medicine, University of Turku, Turku, Finland; 4 Centre for Population Health Research, Turku University Hospital and University of Turku, Turku, Finland; 5 Institute of Biomedicine, Integrative Physiology and Pharmacology Unit, University of Turku, Turku, Finland; 6 Novo Nordisk Foundation Center for Basic Metabolic Research, Faculty of Health and Medical Sciences, University of Copenhagen, Copenhagen, Denmark; 7 Department of Paediatrics and Adolescent Medicine, Turku University Hospital and University of Turku, Turku, Finland; 8 Institut de Pharmacologie Moléculaire et Cellulaire, Université Côte d’Azur & Centre National pour la Recherche Scientifique (CNRS), Valbonne, France

**Keywords:** Molecular biology, Biomarkers

## Abstract

Childhood maltreatment exposure (CME) increases the risk of adverse long-term health consequences for the exposed individual. Animal studies suggest that CME may also influence the health and behaviour in the next generation offspring through CME-driven epigenetic changes in the germ line. Here we investigated the associated between early life stress on the epigenome of sperm in humans with history of CME. We measured paternal CME using the Trauma and Distress Scale (TADS) questionnaire and mapped sperm-borne sncRNAs expression by small RNA sequencing (small RNA-seq) and DNA methylation (DNAme) in spermatozoa by reduced-representation bisulfite sequencing (RRBS-seq) in males from the FinnBrain Birth Cohort Study. The study design was a (nested) case-control study, high-TADS (TADS ≥ 39, *n* = 25 for DNAme and *n* = 14 for small RNA-seq) and low-TADS (TADS ≤ 10, *n* = 30 for DNAme and *n* = 16 for small RNA-seq). We identified 3 genomic regions with differential methylation between low and high-TADS and 68 tRNA-derived small RNAs (tsRNAs) and miRNAs with different levels in males with high CME (False discovery rate, FDR corrected *p* < 0.05). Of potential interest, we identified differential expression of miRNA hsa-mir-34c-5p and differential methylation levels near the *CRTC1* and *GBX2* genes, which are documented to control brain development. Our results provide further evidence that early life stress influences the paternal germline epigenome and supports a possible effect in modulating the development of the central nervous system of the next generation.

## Introduction

Adverse childhood experiences (ACEs) include harms that affect children indirectly through their living environments (e.g., parental conflict, substance abuse, or mental illness) or directly (abuse and neglect). The direct harms are commonly described as childhood maltreatment exposure (CME). CME is highly prevalent, as shown by a recent systematic review and meta-analysis that reported a pooled prevalence of ca. 23% in Europe and the U.S. for adults who reported at least one ACE [1]. Worldwide, as many as 12% of adults report a history of childhood sexual abuse, 23% of childhood physical abuse, and 36% of emotional abuse [2]. ACEs have numerous adverse consequences for later health, via a range of hormonal, metabolic, and immunological pathways [3], especially for mental health outcomes [2, 4]. In addition to affecting health later in life [5], accumulating evidence indicates that paternal ACEs /CME may also affect the health of the next generation [6–10].

Animal studies of paternal inheritance induced by early life stress have shown that advert psychological exposures change the epigenetic marks in sperm and the metabolic and behavioural phenotype of the offspring [11–15]. The commonly recognized epigenetic marks are DNA methylation, histone modifications, and expression of small non-coding RNAs (sncRNA) [16–19]. When carried in gametes, these epigenetic marks have the potential to change the early embryonic developmental trajectory and affect offspring phenotype. Numerous studies have identified a link between paternal exposure to a plethora of physical environmental exposures like toxins, cigarette smoking, physical activity, and nutritional stress and changes in sperm epigenome or altered offspring phenotype [16, 20]. However, the association between early-life psychological stress and epigenetic changes in spermatozoa remains unclear.

To our knowledge, three human studies have established a link between paternal early-life stress and changes in the sperm epigenome have been published to date. In the first, childhood Trauma Questionnaire (CTQ) and Conflict Tactics Scales (CTS) were used to quantify CME in 34 males (17 men exposed to high, 5 men to medium, and 12 men to no childhood abuse), an association was found between DNA methylation and CTQ/composite abuse score [8]. In this cohort, 12 DNA regions were found differentially methylated between individuals with different childhood abuse (high vs. low), including genes associated with neuronal function (*MAPT*, *CLU*), fat cell regulation (*PRDM16*), and immune function (*SDK1*) [8]. In the second study, CME was quantified using an adverse childhood experiences (ACEs) screen; and comparing a group of individuals with the highest ACE score to individuals with the lowest score, a negative correlation was found between levels of multiple miRNAs of the miR-449/34 family and ACE scores [6]. In the third study (preprint at the time of writing), a longitudinal approach that used three different age groups, sperm miR-16 and miR-375 levels were higher in the serum of children exposed to paternal loss and maternal separation (ages 7–12 years) compared to controls [21]. This study also showed similar patterns in another cohort of 18–25-year-olds and, using the CTQ to quantify ACEs, report that the same miRNA species have lower expression in sperm of adult men exposed to higher CME at ages 21–50 years [21]. Interestingly, they also replicate prior findings [6] on lower levels of miR-34 but not miR-449.

In the current study, we measured CME using the Trauma and Distress Scale (TADS) questionnaire and quantified sperm sncRNA profiles and DNAme from males that were divided into 2 groups, a control group with low TADS scores (TADS ≤ 10) and a case group with high TADS scores (TADS ≥ 39). Following the primary analyses, we also performed replication analyses to prior work where possible. This was an exploratory study and no a priori hypotheses on the strength or direction of the possible associations between CME and sperm DNA methylation and sncRNA profiles.

## Material and Methods

The study was conducted in accordance with the Declaration of Helsinki and was approved by the Ethics Committee of the Hospital District of Southwest Finland (15.3.2011 §95, ETMK: 31/180/2011). We followed the Strengthening the Reporting of Observational Studies in Epidemiology (https://www.strobe-statement.org/) reporting guideline (case-control studies v4). All participants signed an informed consent for participating to the studies.

### Participants

Participants were recruited at gestational week (gwk) 12 from maternity clinics in Southwest Finland from 2011 to 2015 to take part in the FinnBrain Birth Cohort Study (http://www.finnbrain.fi), which was established to prospectively investigate the effects of early life stress, including prenatal stress exposure, on child brain development and health. The cohort entails 3808 families and included full trios (mother, father, child) on approximately half of the families [22]. The fathers of the cohort were later recruited separately for these visits when their children were approximately 9 years of age.

The division into case and control groups was based on previously collected data. CME was assessed by questionnaires filled in by the fathers-to-be following the initial recruitment approximately at gwk 14 (2011–2015). There was no case - control matching during recruitment although the approach and analyses in the current study are based on case - control design. The new data were collected during visits that took place between 02/2019 – 07/2021. The recruitment rate for the visits has been 59.6%, which is typical for our cohort study. Until the time of writing, this has been a cross-sectional study.

**Fig. 1:**
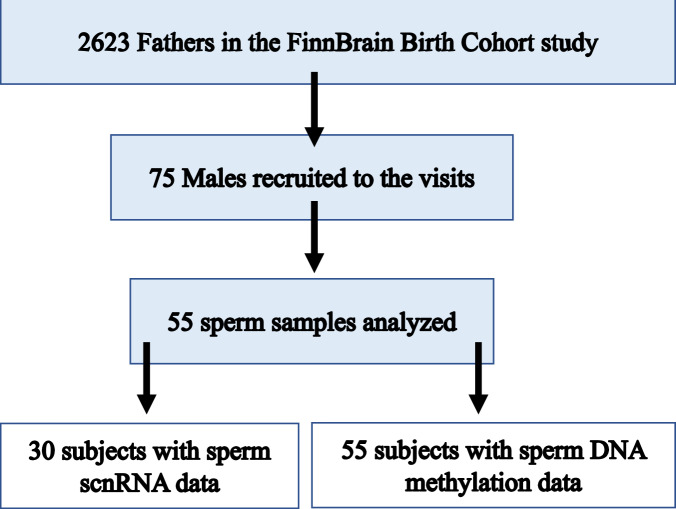
Flowchart of the participant selection. The participants were a subsample from FinnBrain Birth cohort study and the flowchart shows the participant selection that for the current study was based on low or high CME questionnaire scores.

Altogether 75 males participated in the current study. The final sample size was determined by the available resources and comparison to prior studies with similar design. We processed sperm samples from 58 individuals with TADS scores ranging between 0 and 78. 55 samples were analyzed by RRBS-seq to identify DNA methylation patterns, and 30 were analyzed by small RNA-seq (27 samples overlapping). The flowchart of participant selection is presented in Fig. 1. The personnel carrying out the sperm epigenome analyses were blind to the group allocation.

The bias assessment is as follows: there was no systematic monitoring for selection bias during recruitment as the primary recruitment for the cohort entailed a population-representative sample and was fairly uniform on e.g., for participants’ age range; we strived to quantify the influence of potential confounding factors by including multivariate statistical tests; questionnaire-based measurements are prone to measurement error and TADS questionnaire to recall bias, but these limitations apply to all similar studies; finally we performed the processing of semen samples with utmost care and where possible assured their quality to reduce measurement errors.

### The demographics and questionnaire data

Data were mostly obtained over the prenatal period and data collection was continued postnatally. Information about the parents’ CME was collected using the Trauma and Distress Scale (TADS) at gwk 14 [23]. The TADS comprises five core domains: emotional neglect, emotional abuse, physical neglect, physical abuse, and sexual abuse. In this study, we calculated the cumulative exposure to early life stress events of the infants’ fathers and mothers by the age of 18 years (direct sum scores). Of note, the TADS scores have two possible derived values so that one can use direct sum scores or sums of factor scores [23]. Here we used the direct TADS sum scores as the primary variable to perform case-control divisions and statistical testing, but we have used the factor sum scores in our prior article [24]. The direct and factor sum scores were highly correlated (in our sample Spearman’s rho = 0.954, *p* < 0.001).

Depressive symptoms were assessed by implementing the Edinburgh Postnatal Depression Scale (EPDS) [25]. This 10-item questionnaire is scaled from 0 to 30 points with a bigger score denoting increased symptom severity. Anxiety symptoms were quantified with the anxiety subscale of Symptom Checklist 90 (SCL-90) [26]. The SCL-90 anxiety subscale consists of 10 items with a total score range of 0 to 40 with larger scores denoting increased symptom severity. Alcohol use was quantified with a structured questionnaire [27], and the derived variable we chose was mean alcohol use per week (1 unit = 12 g of alcohol). Smoking was quantified with a yes/no binary variable. BMI was obtained from height and weight measurements performed during the research visits that are described below.

### The study visits

Participants that matched the case-control criteria (control group TADS ≤ 10; and case group TADS ≥ 39) were invited to a separate study visit. During the visit fathers filled in questionnaires for smoking and alcohol use habits, depressive symptoms (EPDS), and anxiety symptoms (SCL-90). They also gave additional biological samples (not reported here). Weight and height were measured for defining body mass index (BMI), and waist circumference was measured following standard procedures. Sperm samples were collected before or at the visit.

### The sperm sample collection

Participants were allowed two options for delivering the semen samples following 2-7-day abstinence from ejaculation: either collecting the sample at home and delivering it to the research site at the start of the visit or giving the sample during the visit. The semen was collected by masturbation. Obtained samples were incubated at +37 °C for 5–30 min for liquefaction, and spermatozoa were purified by centrifuging through 50% Puresperm (Nidacon) solution at 400 x *g* for 15 min. The sperm pellet was subsequently washed with mild somatic cell lysis buffer (0.01% SDS, 0.005% Triton X-100) to eliminate remaining somatic cell contamination. The purity of the samples was assessed by light microscopic analysis. The total number of spermatozoa before purification ranged between 40 and 800 million (only one high-TADS sample had less than 40 million spermatozoa), and after purification, all samples were pure with only very minor somatic cell contamination (Supplementary Fig. 1A). Sperm purification was conducted promptly within the same day and purified samples were frozen for storage.

### Small RNA sequencing

#### Sample preparation

Total RNA was extracted from 14 high-TADS (TADS ≥ 39) and 16 low-TADS (TADS ≤ 10) samples (10 million spermatozoa per sample) by TRIzol LS (Invitrogen) containing tris(2-carboxyethyl) phosphine (TCEP, Sigma-Aldrich) as a reducing agent to enhance sperm nucleus lysis and precipitated with isopropanol in the presence of 2 µl of GlycoBlue (15 mg/ml, Invitrogen). After DNaseI treatment (Sigma-Aldrich) without heat inactivation, RNA was re-extracted with Trizol LS to remove DNaseI. The quality of the RNA sample was analyzed by Bioanalyzer (Agilent RNA 6000 Pico Kit). Bioanalyzer analysis validated the lack of somatic cell contamination, as demonstrated by the absence of ribosomal RNA peaks and low RIN values [2, 3] (Supplementary Fig. 1B). The RNA yield from 10 million spermatozoa was 10–200 ng. Libraries for small RNA-seq were prepared using NEB Next® Multiplex Small RNA kit (New England Biolabs), and the libraries were sequenced by NovaSeq 6000 system (Illumina).

#### SncRNA-Seq processing

The quality of the reads was assessed using FastQC (v0.11.9) (http://www.bioinformatics.babraham.ac.uk/projects/fastqc/). Adapters and low quality reads were trimmed off using cutadapt (v3.5) [28, 29] was used to map the 15-45 nucleotides long reads first to the human genome (hg38), then subsequently to ribosomal RNAs (rRNAdb), miRNAs (miRBase v22), YRNA mapping and transfer RNAs (tRNA) (GtRNAdb v2.0) using software default settings. Sequences mapped to tRNAs were annotated as 5’ end-, 3’ end-, or 3’ CCA end-derived according to their location on the parental tRNA sequence. All sperm samples showed typical size distribution of averaged sncRNA reads, including a peak at 21-23 nt for miRNAs, a peak at 31-32 nt for YRNAs, a peak at 31-32 nt for tRNA-derived small RNAs (tsRNAs), and a peak at 31-33 nt for PIWI-interacting RNAs (piRNAs) (Supplementary Fig. 1C, D). All sequences mapping to rRNAs, miRNAs, and tRNAs were extracted from SPORTS1.1 as a text output file. For piRNA analysis, reads were mapped to piRNA clusters [30] in the Human genome (UCSC: Hg38) using HISAT2 (v2.1.0) [31], and assigned and counted using featureCounts (v2.0.0) [32–34] against reference gtf files. Altogether, we identified unfiltered normalized reads mapping to a total of 838 miRNAs, 266 tsRNAs, 6195 piRNA genomic clusters, 8 rRNA genes (12S-rRNA, 16S-rRNA, 18S-rRNA, 28S-rRNA, 45S-rRNA, 5.8S-rRNA, 5S-rRNA, other-rRNA) and 4 YRNA genes (YRNA1, 3, 4 and 5). Raw read counts were filtered to a minimum of 10 total counts across all 35 samples and normalized.

### Reduced Representation Bisulfite Sequencing (RRBS)

#### Sample preparation

Sperm DNA was extracted from ~10 million spermatozoa/sample by lysing cells in RLT+ buffer (Qiagen) supplied with 1% Beta-mercaptoethanol for 15 min, and then passing the lysate through AllPrep Mini Spin Columns (Qiagen) and proceeding with manufacturer’s instructions. The quantification was done by measuring the DNA concentration on a Qubit fluorometer using the Qubit dsDNA High Sensitivity Assay Kit (Thermo Fischer), and was further confirmed by comparing to the quantities measured on a Nanodrop 2000 Spectrophotometer (Thermo Fischer). The quality was further assessed by looking at the 260/280 ratio on the Nanodrop, and samples were only accepted with a ratio exceeding 1.8. The reduced-representation bisulfite sequencing (RRBS-seq) was performed by the Single-Cell Omics platform at the Novo Nordisk Foundation Center for Basic Metabolic Research, University of Copenhagen. RRBS libraries were constructed from 100 ng genomic DNA using the Ovation® RRBS Methyl-Seq library preparation kit (Tecan) according to the manufacturer’s instructions. Final libraries were quantified by Qubit (Thermo Fisher) and quality checked on Bioanalyzer (Agilent). Pooled libraries were subjected to either a 101-bp single-end sequencing on a NovaSeq 6000 platform (Illumina) or a 76-bp single-end sequencing on a NextSeq 500 platform (Illumina). A total of 5.5 billion reads were generated.

#### RRBS processing

FASTQ files were first generated using bcl2fastq (v. 2.20.0) and subsequently trimmed using Trim Galore! (v. 0.6.4) as described in The Analysis Guide for NuGEN Ovation RRBS Methyl-Seq (https://github.com/nugentechnologies/NuMetRRBS). Similarly, diversity adapter sequences were removed using the supplied trimRRBSdiversityAdaptCustomers.py script. Methylation coverage was extracted using Bismark by aligning reads to the GRCh38 assembly. Here, the deduplicate_bismark step was performed using the --barcode option to deduplicate reads based on UMIs, and the --ignore 3 parameter was used during methylation extraction to disregard restriction enzyme sites.

### Statistical analyses

In line with prior work, we used between-group comparisons to identify features associated with CME in the sperm epigenome, comparing those with low to those with high CME. These discovery analyses were followed by multivariate analyses that included potential confounders. Third, we performed replication analyses by Dickson et al. [6]. The multivariate and replication analyses were performed with JASP 0.16.3 (https://jasp-stats.org/). Most variables reported in Table 1 had non-normal distributions and as this applied to the majority of sperm epigenetic variables as well, we thus used non-parametric correlation statistics in multivariate and replication analyses.

**Table 1:** Descriptive statistics of demographics across the whole sample (*N* = 55).

	Whole sample *N* = 55		Low CME *N* = 26		High CME *N* = 29		High/Low CME group difference	
							*Mann Whitney U test*	
Continous variables	Mean	SD	Mean	SD	Mean	SD	*p*	Rank-Biserial
								Correlation
Age (years)	38.764	5.910	36.828	4.699	40.583	6.593	0.054	−0.310
Body mass index (kg/m2)	26.197	4.057	25.869	3.912	26.790	4.357	0.589	−0.089
Waist circumference (cm)	92.095	11.184	92.369	11.824	91.917	11.003	0.754	0.052
EPDS score	3.505	3.317	2.303	2.436	4.750	3.391	0.005	−0.451
SCL-90 score	3.455	3.452	2.276	2.814	4.917	3.741	0.002	−0.493
Average daily alcohol consumption	0.483	0.531	0.631	0.645	0.315	0.295	0.046	0.319
TADS factor sum	13.321	14.350	1.034	1.017	28.167	6.631	<0.001	−1.000
TADS direct sum score	24.528	22.655	4.690	2.480	48.500	7.553	<0.001	−1.000
Semen sample volume (ml)	3.182	1.490	3.410	1.435	3.000	1.573	0.227	0.195
Sperm concentration 10exp6/ml	113.724	84.667	109.800	78.234	107.304	85.355	0.837	0.034
Purified sperm concentration 10exp6/ml	167.213	153.247	177.193	163.072	152.346	147.700	0.642	0.076
**Categorical variables (frequencies)**		***N***	***N***		***N***			
Educational level	low	16	4		12		0.273	1.201^a^
	mid	17	11		6			
	high	22	11		11			
Relationship status	single	2	1		1		0.744	0.107^a^
	married	35	15		20			
	cohabitation	15	7		8			
	separated	3	3		0			
							0.321	0.134
Smoking	Yes	19	10		9			
	No	36	16		20			

^a^Kruskall Wallis test statistic

### Discovery analyses (high vs. low CME group comparisons)

Differential expression analysis of sncRNAs was performed using DESeq2 (v1.36.0) which is based on a negative binomial generalized linear model [35]. RsRNA and YRNAs were analyzed using a Wilcoxon-rank exact test. All 30 samples were split into 2 groups, low-TADS (TADS ≤ 10, *n* = 16) and high-TADS (TADS ≥ 39, *n* = 14). Low-TADS group was defined as the control group. Because of the small sample size, we opted to carry out the discovery analyses as a simple between groups comparison but provide multivariate analyses for all identified sncRNAs as outlined below.

For DNA methylation analysis, the methylation coverage files were analysed using the DMRichR R package (v. 1.7.8). In brief, all 55 sperm DNA samples were split into 2 groups, low TADS (TADS < 10, *n* = 30) and high TADS (TADS > 40, *n* = 25). DMRs were subsequently detected using the DMRichR::DM.R-command. Specifically, the TADS group was supplied as the test-covariate, and the following variables were included for adjustment of possible confounding effects: BMI, age, smoking, daily alcohol consumption, summed SCL-90-R scores (assessing current psychological symptoms) summed EPDS-scores (assessing current depressive symptoms), semen volume and purified semen concentration.

All statistical tests were based on two-sided *p* values. Multiple comparison correction for both scnRNA and DNAme analyses was carried out using Benjamini and Hochberg procedure yielding false discovery rate corrected p values [36].

### Multivariate analyses for sncRNAs with covariates

We performed three different multivariate analyses with partial correlations testing for the association between low vs high CME: (A) controlling for semen sample volume and sperm concentration; (B) controlling for health characteristics including age, BMI, smoking (yes/no), average alcohol use per day, as well as depressive and anxiety symptoms at the time of the sperm sample collection; C) controlling for covariates in A + B.

### Replication analyses of prior work (miRNAs hsa-miR-34c-5p and hsa-miR-449a)

We also performed replication analyses of Dickson et al. [6] for the sncRNA data by exploring the associations between miRNA that their work implicated important and ACE exposure. For this, we looked at between-group differences (low vs. high ACE exposure; reported in Supplementary Fig. 4) and Spearman correlations between CME (TADS scores) and miRNA expression levels for hsa-miR-34c-5p and hsa-miR-449a. We chose to use the TADS factor scores here as the variable had a “more normal” distribution (correlation to TADS sum score Spearman rho = 0.954). We extended these analyses also to partial correlation models where we controlled for age and BMI at measurement, and smoking (yes/no).

## Results

We analyzed the association between CME, as quantified by TADS scores, and two epigenetic features in sperm: the abundance of sncRNAs and levels of DNA methylation. The study enrolment is presented as a flowchart (Fig. 1), demographics are reported in Table 1, and correlations across the sperm characteristics and demographics are available in Supplementary Table 1.

### Childhood maltreatment exposure is associated with modified sperm sncRNA profile

**Fig. 2:**
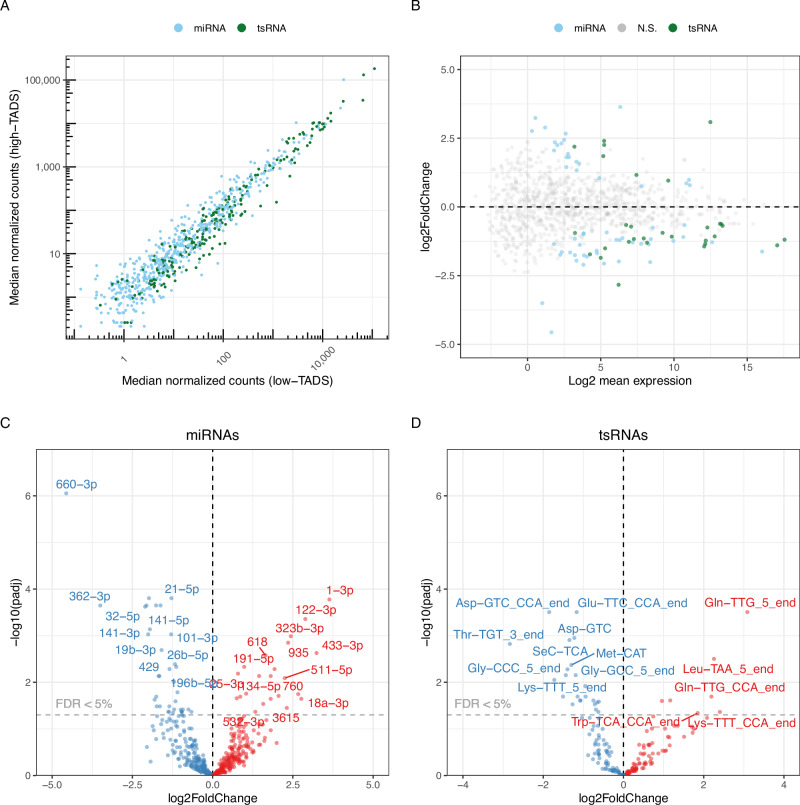
Differential levels of sperm sncRNAs in high-TADS vs low-TADS sperm samples. **A** Scatter plot shows sncRNA expression between high- and low-TADS score groups. One dot represents the median of normalized counts of all samples belonging to either high- or low-TADS group. **B** MA plot displays the relationship between the log2 fold change in high-TADS vs low-TADS samples and log2 mean expression for all miRNAs and tsRNAs. **C** Volcano plots show differential expression of miRNAs in high- vs low-TADS samples. **D** Volcano plots show differential expression of tsRNAs in high- vs low-TADS samples. Blue and red colours refer to downregulated and upregulated sncRNAs respectively.

The analysis of small RNA-seq data from 14 high-TADS and 16 low-TADS sperm samples showed that the abundance of five analysed classes of sncRNAs was generally similar between high-TADS and low-TADS groups (Fig. 2A). The vast majority of sncRNA reads originated from rsRNAs and YRNAs (Supplementary Fig. 1). The reads derived from YRNA and rRNA genes were similarly distributed in high- and low-TADS sperm samples, with some differences in the relative number of reads derived from 12S-rsRNA, 16S-rsRNA, 5.8-rsRNA, RNY1 and RNY4 between high- and low-TADS samples (Supplementary Fig. 1E).

Differential expression analysis of individual miRNAs, tsRNAs, and piRNA clusters showed significant differences in high-TADS vs. low-TADS sperm (Fig. 2). The expression of a total of 29 miRNAs, 15 tsRNAs, and 3 piRNA clusters were lower in high-TADS groups sperm compared to low-TADS sperm (log2FC < -1.0 and *P*_adj_ < 0.05), while correspondingly 18 miRNAs, 6 tsRNAs, and 1 piRNA cluster were higher (log2FC > 1.0 and *P*_adj_ < 0.05) (Fig. 2B–D, Supplementary Tables 2–4). The expression levels of three tsRNAs and five miRNAs showing the most significant changes (log2FC > 2, and P_adj_ < 0.01) and the number of normalized counts among all samples above the threshold “baseMean > 10” are plotted in Supplementary Fig. 2. Interestingly, hsa-miR-34c-5p, which was earlier shown to be lower in sperm samples of individuals with high ACE scores [6, 21], was also lower in our discovery analysis (Supplementary Fig. 2H).

### CME associations are generally robust to potential confounders in multivariate analyses

We identified the following potentially relevant covariates and confounders, based on prior literature, that were also measured in the current study (Table 1, Supplementary Table 5): sperm sample volume, sperm concentration, age, BMI, smoking, alcohol use, depressive symptoms, and anxiety symptoms. The multivariate statistical analyses showed that the associations that were implicated in the discovery analyses were very robust to include covariates. Most of the associations remained statistically significant after controlling for all potential confounders (Supplementary Tables 6 and 7).

### Expression of hsa-miR-34c-5p is robustly associated with CME across studies

**Fig. 3:**
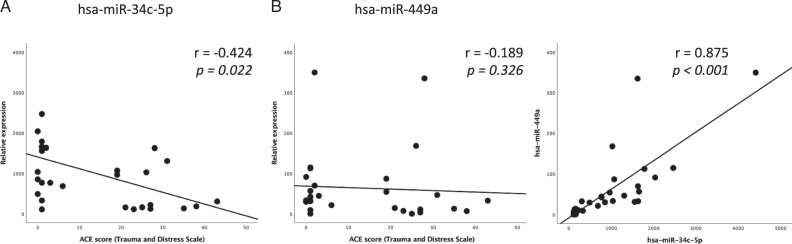
The associations between TADS scores and miRNA expression levels that were identified in prior work. **A** We found a negative association between ACE score and hsa-miR-34c-5p, **B** but no associations for hsa-miR-449a. The scatter plots correspond to prior report by Dickson et al. [6], Figure 2.

Some associations between TADS scores and the expression of different miRNA in sperm were previously identified [6]. In line with prior work, our discovery analysis showed that the relative expression levels of hsa-miR-34c-5p was lower in high early life stress exposure group. A separate correlation-based replication analysis returned a negative association between ACE score and hsa-miR-34c-5p with a similar effect size, but no associations for hsa-miR-449a levels (Fig. 3A and B; replicating Fig. 2 plots of Dickson et al.). Like shown previously, the expression of the hsa-miR-34c-5p and hsa-miR-34c-5p (Fig. 3C). Partial correlation analyses for the depicted associations and controlled for age and BMI at measurement, and smoking (yes/no) identified associations between hsa-miR-34c-5p and TADS score (r = −0.528, *p* = 0.001), but not between hsa-miR-449a and TADS score (r = −0.185, *p* = 0.339). Of note, the association of hsa-miR-34c-5p vs. TADS score was slightly weaker when controlling for all covariates (r = −0.420, *p* = 0.073) (Supplementary Table 6). We next performed corresponding between-group comparisons (Supplementary Fig. 3; replicating plots in Dickson et al. Figure 1). We replicated the lower expression levels in ACE exposed group for hsa-miR-34c-5p (W = 140, *p* = 0.017, rank biserial correlation [rbc] = 0.538) and that there were no differences in expression levels of hsa-miR-152-3p and hsa-miR-375-3p. We replicated the differences between low/high CME groups in the discovery analyses and replication analyses as well as the negative correlation between continuous CME scores and hsa-miR-34c-5p. Our analyses thus show that sperm expression of hsa-miR-34c-5p is robustly negatively associated with CME.

### Childhood maltreatment exposure is associated with modified sperm CpG methylation

**Fig. 4:**
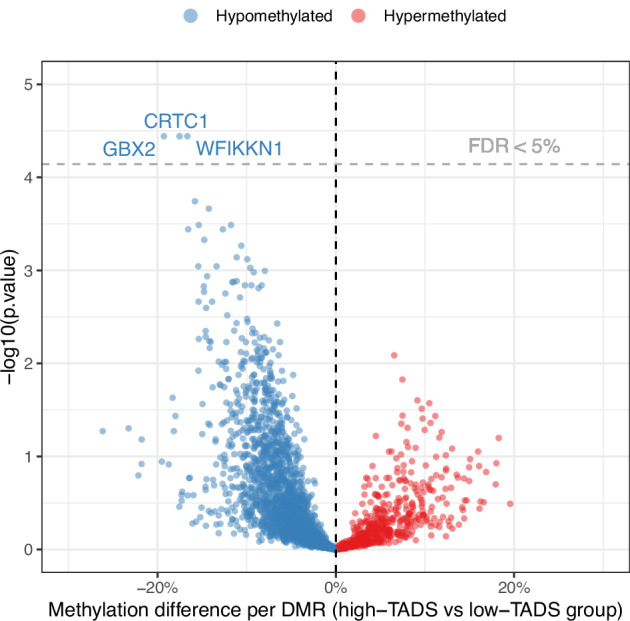
Differential levels of CpG methylation in high-TADS vs low-TADS sperm samples. Each dot represents one candidate genomic region tested for differential methylation. The three regions that showed statistically significant group differences after correction for multiple testing (FDR-corrected *p* values < 0.05) are annotated according to the nearest gene.

To investigate if early life stress experience is associated with particular sperm DNA methylation signatures, we measured DNA methylation levels across CpG-rich regions using Reduced Representation Bisulfite Sequencing (RRBS). We identified 3 differentially methylated regions (DMRs) between the high and the low TADS group (false discovery rate, FDR < 0.05, Fig. 4, Supplementary Fig. 4, and Supplementary Table 8). These DMRs are located in the 3’ UTR of the CREB Regulated Transcription Coactivator 1 gene (*CRTC1)*, the promoter WAP, follistatin/kazal, immunoglobulin, kunitz and netrin domain containing 1 gene (*WFIKKN1*), and in the distal intergenic region nearest the Gastrulation Brain Homeobox 2 (*GBX2)* gene.

Using pyrosequencing, we validated our RRBS results on a selection of genomics regions identified by RRBS; three regions with FDR below 0.2 and two with FDR above 0.7 (Supplementary Table 9). Consistent with the RRBS results, we detected lower DNA methylation across the statistically significant DMR (FDR < 0.05) in the high TADS group (Supplementary File 1). Thus, CME is associated with distinct DNA methylation signatures in sperm.

## Discussion

Here, we report that CME is associated with specific epigenetic signatures in sperm. We identified differential expression of numerous sncRNAs and 3 genomic regions with hypomethylation in the high TADS score group. We found an interesting overlap with previous reports for miRNAs, particularly miR-34c-5p, although most of our results were distinct from prior reports. Specifically, we discovered that the expression of miRNA hsa-mir-34c-5p and the methylation levels near the *CRTC1* and *GBX2* genes are different in individuals with high TADS scores. Given the documented role of these genes on brain development, we speculate that remodelling of the sperm epigenome by psychological stress during childhood may affect the developmental programming of the embryo post fertilization and affect the function of the central nervous system in the next generation offspring.

### Questionnaires of early life stressors

Early life adversity and stress are usually assessed retrospectively, and such measures are inherently prone to recall bias and they are unable to capture exposures for the youngest ages, e.g., under the age of three years. It is clear, however, that these questionnaires capture useful information about the cumulative stressors that have been present during childhood. Attesting to this ACEs have been associated with multiple adverse health outcomes [4, 37, 38].

We measured early life stress with TADS questionnaire that captures five dimensions of neglect and abuse CME that are also part of the most widely adopted CTQ. The other frequently adopted option is to use questionnaires that quantify ACEs. For instance, the ACE Study Questionnaire includes yes/no answers to 10 questions involving participants’ experiences at home until the age of 18. Five of the questions probe CME: physical abuse, verbal abuse, sexual abuse, physical neglect, and emotional neglect which are also the main features of CTQ and TADS questionnaires. The other five questions probe adversity of family members (that has likely a negative influence on the exposed individual): a caretaker with alcoholism or alcohol abuse, experiencing domestic violence, caretaker incarceration, a family member diagnosed with a mental illness, and the loss of a caretaker through divorce, death, or abandonment [6]. In summary, there are many options for quantifying ACEs and CME [39], and while most of them probe the five main types of childhood maltreatment, there is very little knowledge on similarities and differences between the questionnaires [39].

### Stable epigenetic marks in sperm

The stability of the sperm epigenome is not well known. Indeed, human studies frequently use cross-sectional data, which creates obvious limitations for quantifying measurement error (this limitation applies to the current study as well). Roberts et al. [8] report an intra-class correlation coefficient (ICC) between replicate sperm samples taken ca. 3 months apart for the DNAme profiles that were associated with abuse exposure so that ICC values were higher than 0.7 for 90% of implicated sites. Dickson et al. did not collect replicate samples [6]. The field would benefit from larger-scale studies that describe the “normative” sncRNA and DNAme profiles in the sperm and describe, which components are stable and which ones are more dynamic across months and longer-term e.g., over several years. Commonly used measures of sperm cell epigenetics, small RNAs and DNA methylation (DNAme) patterns, are modifiable through lifestyle factors, health, and environmental exposures [16]. Correspondingly, if ACEs cause epigenetic programming in germ line cells that are relevant to intergenerational inheritance, most of them should be relatively stable following the exposure and they would not increase or decrease in time, which is in contrast to many other studied exposures such as cigarette smoking, exercise, acute stress, diet, and obesity.

### Possible links from sperm epigenome to offspring brain development

Although epigenetic mechanisms following fertilization related to DNAme and sncRNAs are likely essential in typical development and may be closely intertwined [15, 16], the most intriguing and robust evidence supports sncRNAs in conveying epigenetic inheritance [15, 40, 41]. Rodent studies have shown transgenerational inheritance of paternal ACEs via changes in miRNA profiles [12–14, 42, 43]. Similar effects have been reproduced without paternal exposure by injecting the implicated miRNA into zygotes [6, 14, 43].

Prior work implicated that ACEs were negatively associated with the abundance of miRNAs 449/34 in sperm [6, 21]. Importantly, these miRNAs are unexpressed in oocytes but transmitted to them upon fertilization [6, 44], and are key regulators of brain development [45–48], including foetal human brain development [49, 50], and possibly also later in development [51]. Our analyses provided partial replication for prior work by implicating a negative association between ACEs and miR-34 (Fig. 4, Supplementary Fig. 3). As a novel and interesting finding, we identified 21 tsRNAs which had differential abundance in high- vs low-CME participants. In addition to miRNAs, tsRNAs have also been linked to epigenetic inheritance and could be a biomarker for CME in line with miRNAs [52].

Prior studies and the current study identified sperm epigenetic features that could potentially have effects on offspring brain development, which ties in with our recent neuroimaging studies that link paternal CME with offspring neonate brain structure [24, 53, 54]. Several studies, including from members of our group, have identified that lifestyle factors remodel DNA methylation near genes controlling the development of the brain in human sperm [55, 56], supporting that genomic regions involved in brain development are hotspots of epigenetic variation in response to environmental stress.

Our analyses identified three differentially methylated regions near *CRTC1*, *WFIKKN1*, and *GBX2*. Expression of *CRTC1* in the hippocampus has been implicated in mood-related disorders such as depression [57, 58]. Increased methylation in the 3’ UTR region might relate to increased gene expression of *CRTC1* [59]. Expression of *CRTC1* may be decreased in the brain, as methylation in the *CRTC1* DMR in spermatozoa of individuals with high-TADS has the potential to programme *CRTC1* expression throughout embryogenesis [59]. *GBX2* regulates brain development and is linked with neural crest differentiation, dopaminergic neurogenesis, and the formation of the neural plate [60, 61]. Whether the DMR near *GBX2* regulate brain development and neuronal function requires further investigation. *WFIKKN1* regulates the effects of transforming growth factor beta (TGFB) especially in muscle tissue [62].

The reduction in methylation percentage between the high TADS and the control group was in the range of 19-21% for all the statistically significant DMRs. These magnitudes implicate that in one-single ejaculate, a substantial number of spermatozoa are affected by epigenetic reprogramming, representing a potentially high effect size. Thus, in the light of the role of gametic epigenetic signature in embryonic development, remodelling of the sperm epigenome in high TADS individuals may carry biological relevance. While intergenerational effects carried by the sperm epigenome in humans have not been definitively demonstrated, an altered gametic epigenome after CME may influence the development of the central nervous system and modulate the behaviour of the next generation offspring.

### Sperm epigenome as a biomarker of childhood ACEs/CME

Epigenetics is a nascent field with typically small sample sizes that are related to high costs and the need for special infrastructure. This is not too different from the early stages of fields such as genetics and neuroimaging. Within this context, collecting very large data sets is not always feasible. Future studies would benefit from including replicate sperm samples to at least part of the participants to quantify the stable/repeatable elements in both early life stress exposure and control groups. This could potentially be used to identify the most relevant measures of interest for later analyses and greatly decrease the need for multiple comparisons correction. Early life stressors are challenging to quantify reliably, and it may well be that the effects of the exposures are different for different ages, for instance before and after puberty. Prospective cohorts that have detailed information over childhood and measures of early life stressors, including ACEs and CME, could share light on the matter by including sperm data collection e.g., during early adulthood.

Roberts et al. were able to estimate a parsimonious epigenetic marker for childhood abuse using an elastic net model (penalized regression), which identified three DNAme probes that predicted high vs. no childhood abuse in 71% of participants [8]. Such findings are very promising and may lead to parsimonious predictive models in the future. Future mechanistic studies should advance our understanding of how DNAme are affected by the underlying genome, and how sncRNAs and DNAme interact in causal pathways following fertilization. Dickson et al. combined data from human and animal models and were able to show that the effects of their CSI stress paradigm implicate similar sncRNA profiles in mice and that these effects are transmitted to embryos and can thus have inter/transgenerational effects [6]. Studies that focus on RNA are much better able to measure such epigenetic signatures since DNA methylation undergoes erasure and reestablishment following fertilization, which makes studying the immediate post-fertilization effects challenging [16].

### Practical implications

Intergenerational transmission of well-being, health and disease is an important research topic with many implications for health care and societies. It has been postulated that a key component of ACEs, CME is the single most important preventable risk factor for future mental health [1, 4, 37, 38]. CME has also been shown to have effects on health outcomes even when genetic confounding is taken into account [63]. Total annual costs attributable to ACEs were estimated to be US$581 billion in Europe and $748 billion in North America [1, 37]. Over 75% of these costs arose in individuals with two or more ACEs. Elucidating the mechanisms of intergenerational epigenetic inheritance in humans should be of high priority. Finding interventions to intergenerational effects that pass from one generation to the other could potentially spare future generations from the exposures of their ancestors.

While we fully acknowledge the lack of definitive evidence in humans, and the inherent limitations of our study (detailed below), our results may have revealed a mechanism by which early life psychological stress may programme gametes and affect the next generation offspring, through a process referred to as epigenetic inheritance [64]. Since most of the epigenetic signatures that we detected in the high-TADS group were robust to covariates relating to mental health and lifestyle, it is not unlikely that these distinct epigenetic signatures were the result of early life stress specifically, and that these signatures are relatively stable across the male lifespan. We profiled the sperm epigenome across a population of sperm cells of single ejaculates, so that the reported differences reflect the average for each participant. In the hypothetic scenario where a men with CME history would conceive a child, the sperm which would fertilize the egg would be more likely to carry epigenetic features of CME response, such as lower methylation of *CRTC1*, *WFIKKN1*, and *GBX2*, and lower amount of hsa-miR-34c-5p, than individuals without history of CME, provided these epigenetic signatures are not related to lowered fertility [65]. While fertilizing with a spermatozoa carrying altered signatures could have implications to offspring development and phenotype later in life, we acknowledge that epigenetic inheritance per se was not addressed in this study, and that there are many factors that limit the possibilities for demonstrating epigenetic inheritance in humans [66]. Even if CME-induced epigenetic inheritance were to be definitely identified in humans, whether the traits that are passed on are beneficial or deleterious to the offspring across the life course would need long explorations [67].

### Strengths and limitations

We used the largest sample size to date for identifying epigenetic marks relates to CME, but the sample size is still modest and larger sample sizes are needed in the future. Measurement error and test-retest reliability of sperm epigenome were not quantified, which could be done with repeated measurements of sperm epigenome. CME measures were obtained retrospectively and are prone to recall bias, but CME measures are widely used and provide the only tangible way of assessing childhood experiences. All participants were Scandinavian/Caucasian, which makes the source population homogenous but necessitates the inclusion of more ethnically diverse populations in the future. One key strength of our study is that samples were not confounded with epigenetic signatures of infertility. Sperm was indeed collected from men with proven fertility that is, after a pregnancy was achieved. Samples from participants seeking fertility treatment or with unknown fertility status are risking that CME causes fertility issues, associated with altered sncRNA and DNAme profiles.

## Conclusions

In the current study, multiple sncRNAs were found associated with CME. For the majority of sncRNAs differentially expressed in the high compared to low TADS score group, these associations were robust after testing for semen quality and health-related factors as confounders. We discovered 3 regions of the genome carrying distinct DNA methylation profiles in sperm from individuals with history of childhood psychological stress, notably at proximity of genes regulating the development of the central nervous system. Collectively, our findings support that earlier stress exposures, even from as far as childhood, can leave epigenetic imprints in sperm cells that is are stable across the life course. Our work adds to the accumulating number of studies showing that environmental factors at the broad sense influences the epigenome for human sperm, and further opens for determining the consequence of environmentally acquired epigenetic signatures in sperm on the health of the offspring.

## Supplementary information

Supplementary information

## Data Availability

The Finnish law and ethical permissions do not allow the sharing of the data used in this study. Data sharing is possible via formal agreements and interested investigators are requested to contact FinnBrain study administration; (https://sites.utu.fi/finnbrain/en/contact/).

## Code availability

The preprocessing and statistical models were performed with openly available tools, and we report the details in the methods section.
