# Supplementary material for: Exposure to childhood maltreatment is associated with specific epigenetic patterns in sperm
Source: Mol Psychiatry. 2025 Jan 3;30(6):2635–44. doi: 10.1038/s41380-024-02872-3 (PMC12092256; doi:10.1038/s41380-024-02872-3)
Supplement: Supplementary file 1 — Supplementary information [file 41380_2024_2872_MOESM1_ESM.pdf]

Supplementary file 1: Exposure to childhood maltreatment is associated with specific epigenetic patterns in sperm

DNAme validation analysis

Methods

Targeted bisulfite pyrosequencing was performed in the same samples used for RRBS to validate the reported DMRs. Specifically, the top significant DMR (CTRC1, FDR=0.035), as well as two additional nominally significant DMRs with FDR<0.16 (C8orf58/PDLIM2 and FSCN1/RNF216) were chosen as validation targets. In addition, we chose a single, non-significant DMR (MRPL36, FDR=0.74) to serve as a negative control. DNA sequences were retrieved from the GRCh38 assembly using the UCSC genome browser, and theoretical CpGs were therefore included in the primer design. Several primers targeting each region were designed using Qiagen PyroMark Assay Design Software 2.0. Ultimately, one primer pair was selected for each DMR, based on the best genomic overlap with the previously reported regions. Sequences for PCR primer pairs and sequencing primers can be viewed in supplementary table X. Briefly, 200 ng of genomic DNA was bisulfite-converted using the EZ DNA Methylation-Gold Kit (Zymo Research), and 10-15 ng of bisulfite-converted DNA was amplified with the Qiagen PyroMark PCR kit following manufacturer's instructions. Samples were sequenced on the Pyromark Q48 Autoprep, and data was analyzed with the PyroMark Q48 Autoprep software.

Statistical tests

To test for significant differences in methylation levels between the high-TADS and the low-TADS group in the validation data, we modeled the methylation level at each CpG site independently. We used a simple beta-regression model framework (betareg R-package), that appropriately handles 0/100% methylation measurements. Specifically, we used the the TADS-group indicator variable as the main explanatory variable, along with the following covariates: BMI, age, smoking (yes/no), daily alcohol consumption, summed SCL-90-R scores (assessment of current psychological symptoms), summed EPDS-scores (assessment of current depression symptoms), semen volume, and purified semen concentration.

To determine significance at the individual CpG sites, we extracted the P-values for the null hypothesis assuming no effect of the TADS-group variable, and used a significance threshold of P<0.05.

Main findings

We find that the decreased methylation percentage in the high-TADS group compared to the low-TADS group is generally conserved in the validation data for all three DMRs (Supplementary Figure A).

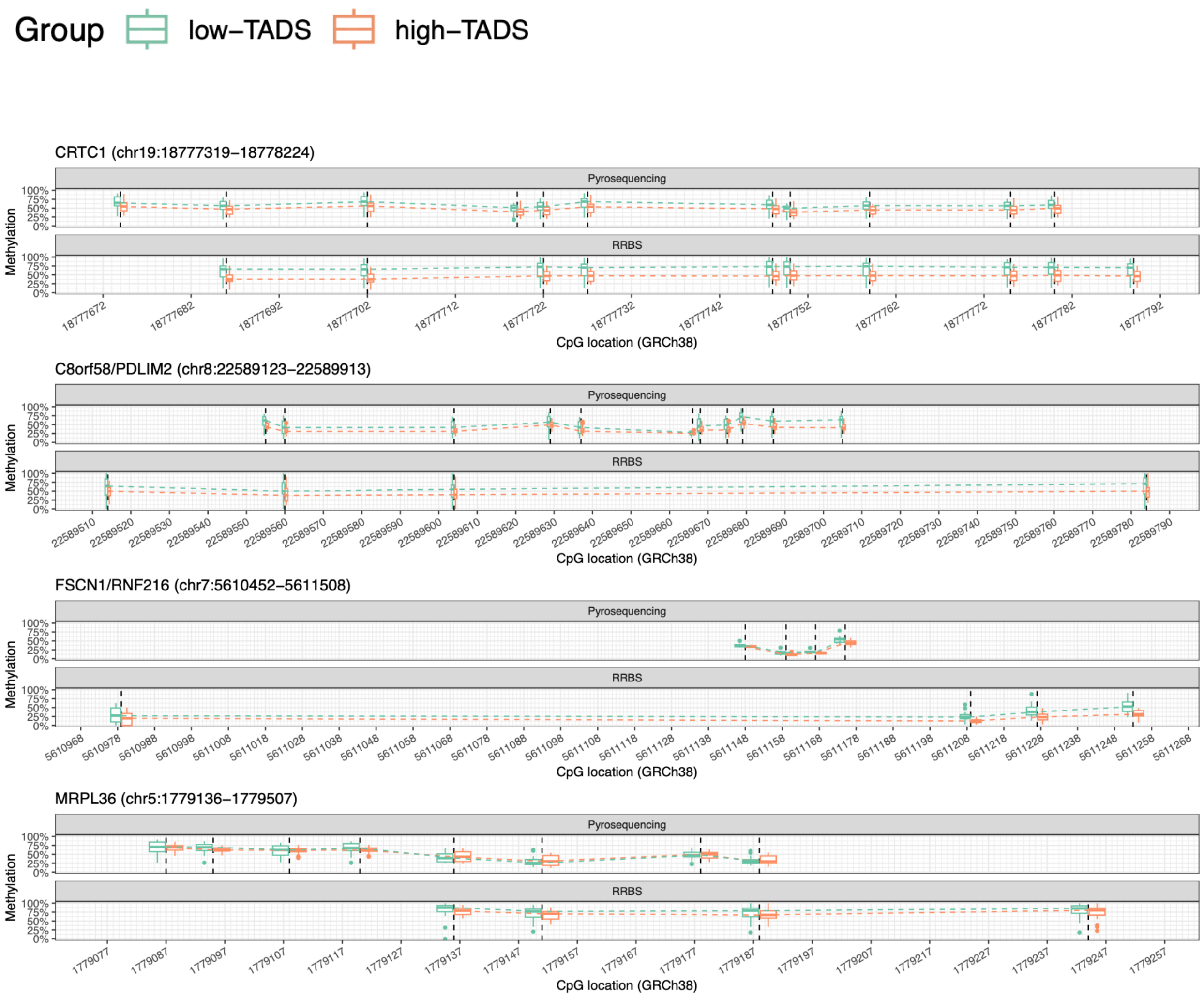

Supplementary figure A: Methylation results from pyrosequencing-based validation of RRBS data. Methylation levels are shown for each CpG site at the four DMRs: CRTC1 (FDR=0.035), C8orf58/PDLIM2 (FDR=0.11), FSCN1/RNF216 (FDR=0.16), and MRPL36 (FDR=0.74). The experimentally observed CpG sites from either pyrosequencing or RRBS are indicated by dashed vertical lines. The median methylation levels between neighboring CpG sites across the low and high TADS groups are connected by green and orange dashed lines respectively.

Notably, there is a significant ($P<0.05$) effect of high TADS at all CpG sites within two of the three tested DMRs: CRTC1/ and FSCN1/RNF216 (Supplementary Figure B). While the use of more complex statistical models that account for correlation between neighboring CpGs would

be preferred, we argue that this approach is sufficient validation of the reported results at the current stage. However, it should be noted that the performed pyrosequencing is only a technical validation of the reported results, and a biological replication by others would therefore be greatly informative.

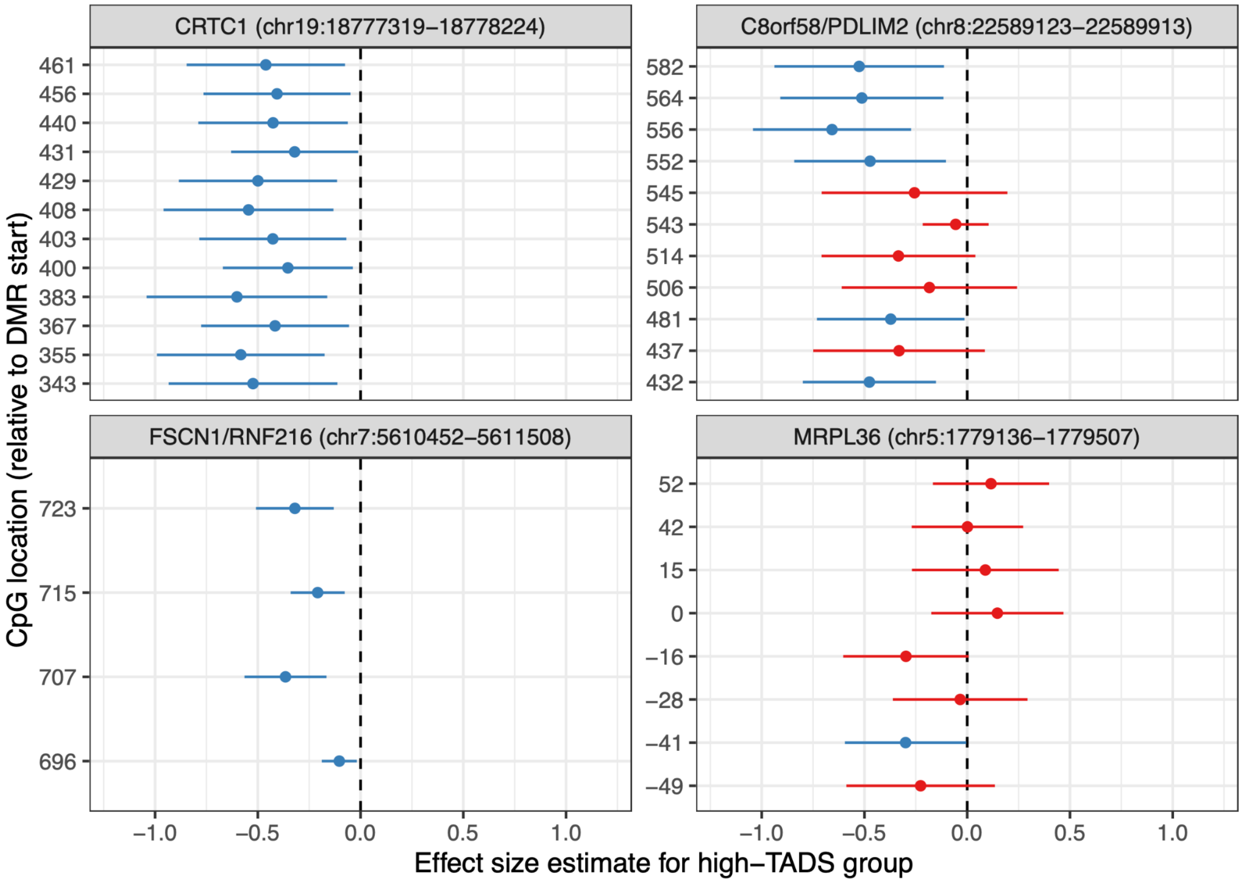

Supplementary figure B: Statistical evaluation of the TADS-group effect in the pyrosequencing validation data. Beta regression effect size estimates with 95% confidence are given on the untransformed scale. A blue color indicates significance (null hypothesis B=0, P<0.05) of the TADS-group variable at the given CpG.

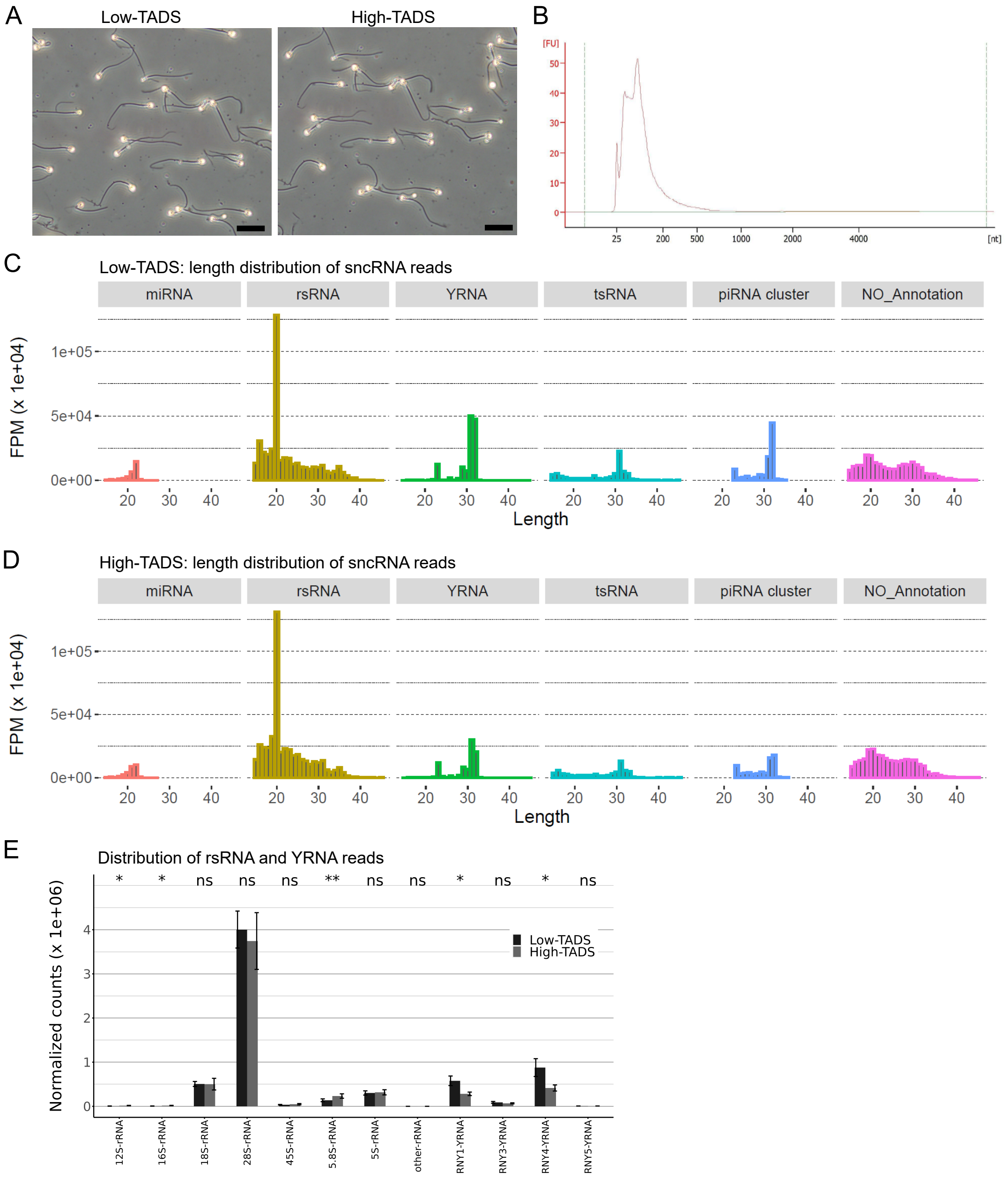

Supplementary Figure 1. Quality control of sperm samples. (A) Phase contrast images of representative unstained sperm samples from low-TADS and high-TADS individuals. Scale bars: 20 µm. **(B)** Representative bioanalyzer profile of sperm total RNA sample (low-TADS). **(C, D)** Size distribution of sperm sncRNA reads, representative examples of low-TADS (C) and high-TADS (D) samples are shown. FPM: average fragments per million. Size of sncRNA reads is indicated in nucleotides on the x-axis (Length). **(E)** Distribution of rsRNA and YRNA reads in low- and high-TADS sperm samples. The bars represent the means ± standard error of the normalized reads (FPM, fragment per million) mapping to different types of rsRNAs and YRNAs in low-TADS (n = 16) and high-TADS (n = 14) samples. Wilcoxon-rank exact test indicated significant differences in the abundance of 12S-rsRNA (W=162, p=0.03827), 16S-rsRNA (W=170, p=0.0152), and 5.8-rsRNA (W=177, p=0.005991), as well as for RNY1 (W=58, p=0.02454) and RNY4 (W=57, p=0.02184).

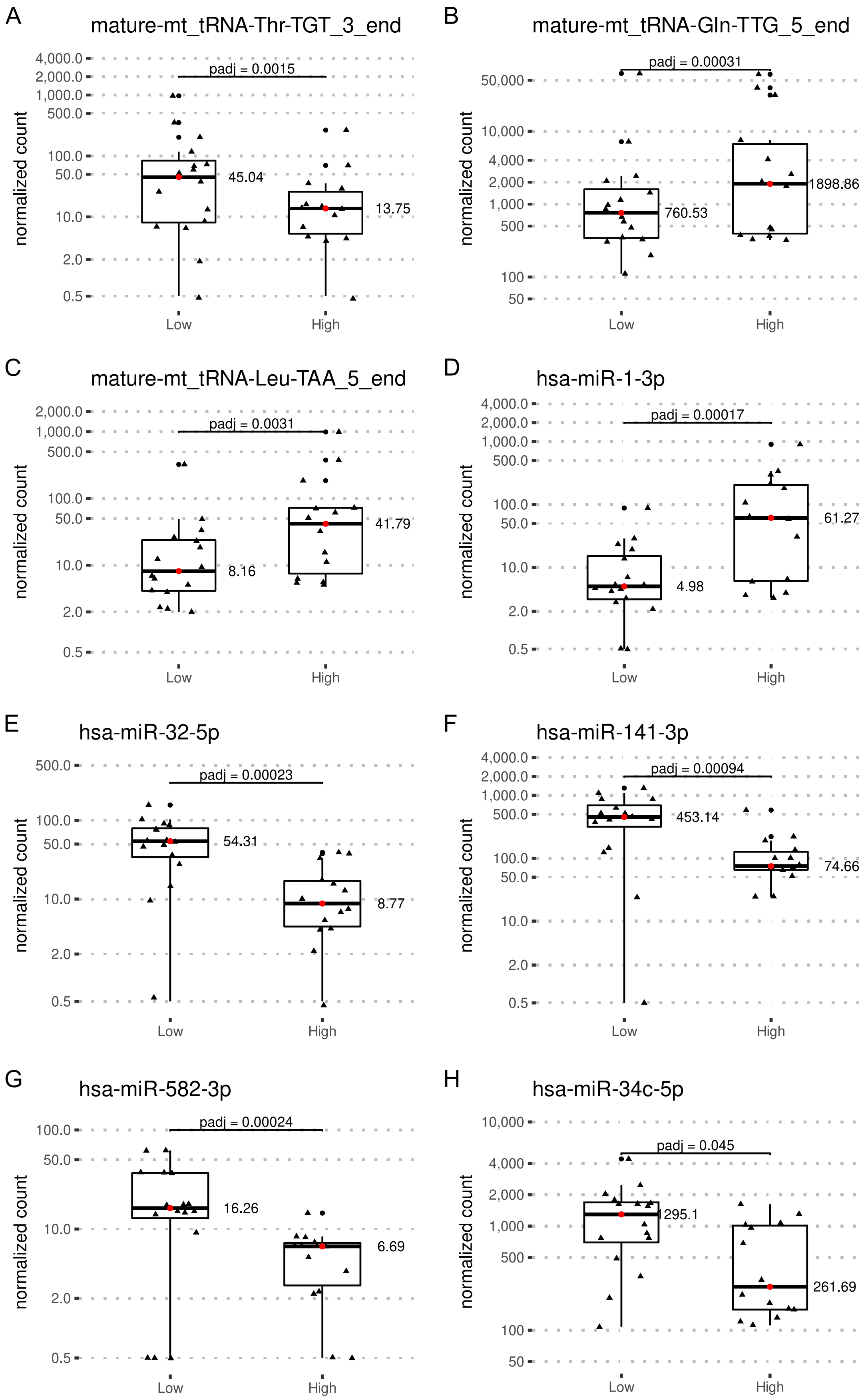
A
mature-mt_tRNA-Thr-TGT_3_end
padj = 0.0015
45.04
13.75
B
mature-mt_tRNA-Gln-TTG_5_end
padj = 0.00031
760.53
1898.86
C
mature-mt_tRNA-Leu-TAA_5_end
padj = 0.0031
8.16
41.79
D
hsa-miR-1-3p
padj = 0.00017
4.98
61.27
E
hsa-miR-32-5p
padj = 0.00023
54.31
8.77
F
hsa-miR-141-3p
padj = 0.00094
453.14
74.66
G
hsa-miR-582-3p
padj = 0.00024
16.26
6.69
H
hsa-miR-34c-5p
padj = 0.045
1295.1
261.69
normalized count
Low
High

Supplementary Figure 2. Expression levels of a selection of individual sncRNAs in high-TADS and low-TADS sperm samples. (A-G) Box plots show the normalized expression levels of selected sncRNAs with an absolute log2 fold change > 2.0, Padj < 0.01 and baseMean > 10. **(A)** mature-mt_tRNA-Thr-TGT_3_end; **(B)** mature-mt_tRNA-Gln-TTG_5_end; **(C)** mature-mt_tRNA-Leu-TAA_5_end; **(D)** hsa-miR-1-3p; **(E)** hsa-miR-32-5p; **(F)** hsa-miR-141-3p; **(G)** hsa-miR-582-3p. **(H)** Box plot show the normalized expression level of hsa-miR-34c-5p (log2 fold change = -1.2, Padj = 0.045 and baseMean = 992). P-adjusted value was calculated by DESeq2 during differential expression analysis.

Supplementary Figure 3. Replication of Dickson et al. 2018 [replicating plots in Figure 1].

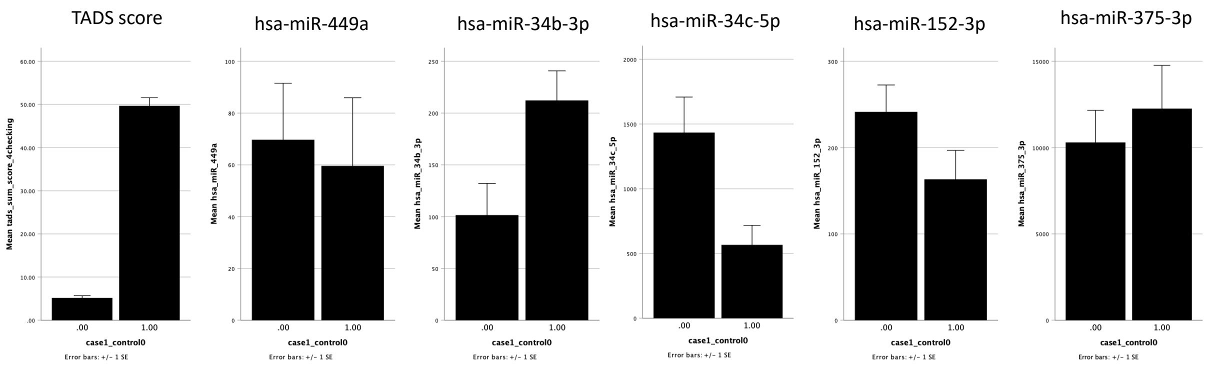

Supplementary figure 3. The bar plots depict mean values and error bars standard deviations to and possible between group differences between low and high ACE exposure and miRNA expression levels that were identified in prior work (Dickson et al. 2018). We replicated the lower expression levels in ACE exposed group for hsa-miR-34c-5p (W = 140, p = 0.017, rank biserial correlation [rbc] = 0.538) and that there were no differences in expression levels of hsa-miR-152-3p and hsa-miR-375-3p. Our analyses did not replicate group differences for has-miR-449a. Finally, we found the opposite group difference for hsa-miR-34b-3p, higher expression levels in the ACE exposed group (W = 24, p < 0.001, rbc = -0.736).

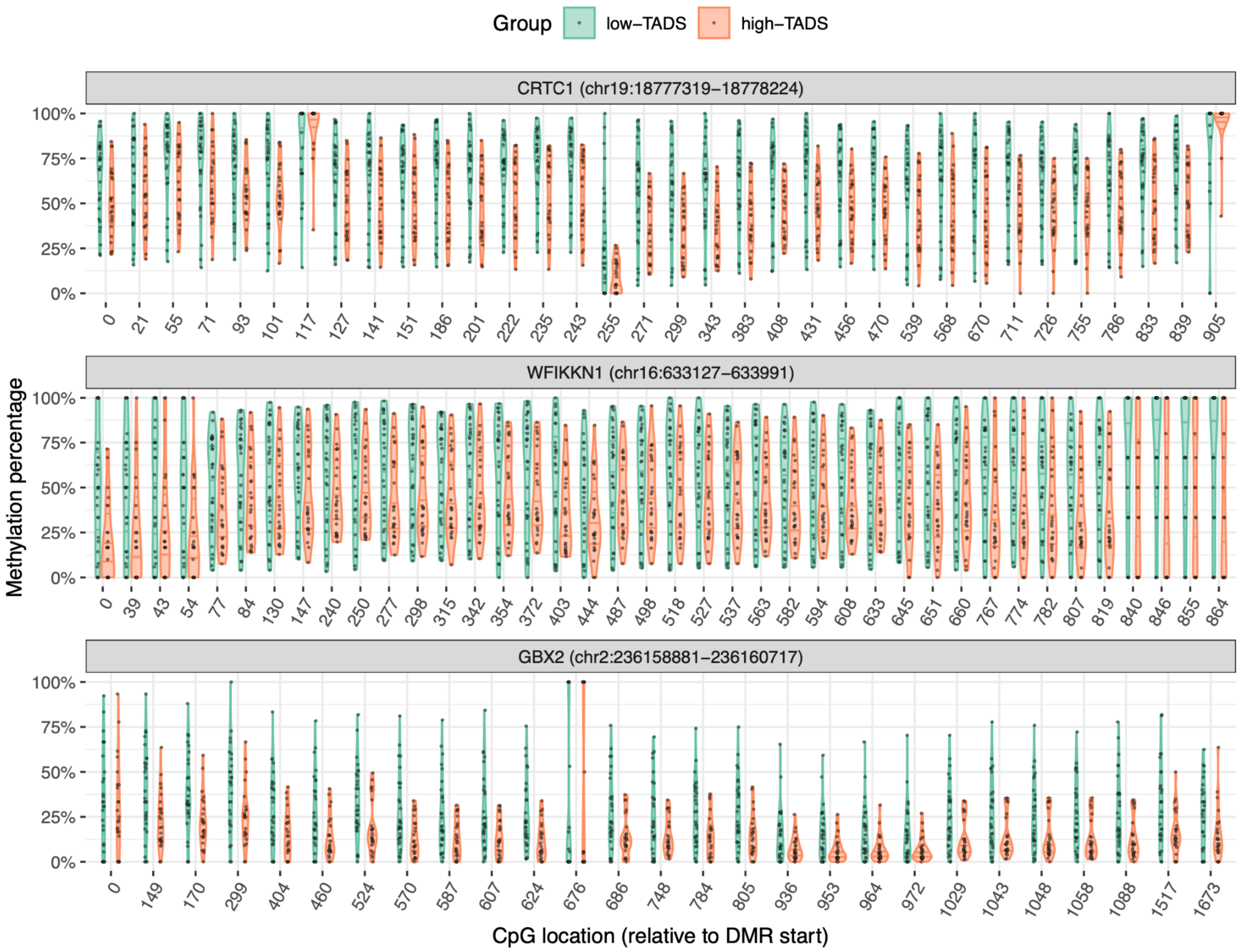

Supplementary figure 4. Raw methylation percentages per individual by TADS-group. Raw methylation levels are unadjusted for covariates. To avoid overplotting, only every second CpG site is visualized at the three DMRs.

Supplementary Table 1. Zero order correlations of the variables presented in Table 1.

Supplement 1. Full correlation matrix for the demographics data; including the TADS case control division and the availability of the sncRNA samples

**Variable**																
1. Age	Spearman's rho	—														
	p-value	—														
2. BMI	Spearman's rho	-0.183	—													
	p-value	0.182	—													
3. waist circum.	Spearman's rho	-0.038	0.876	—												
	p-value	0.785	< .001	—												
4. EPDS	Spearman's rho	0.101	-0.206	-0.144	—											
	p-value	0.464	0.131	0.295	—											
5. SCL	Spearman's rho	-0.013	0.016	0.036	0.631	—										
	p-value	0.927	0.908	0.796	< .001	—										
6. TADS factor sum	Spearman's rho	0.252	0.099	-0.003	0.445	0.371	—									
	p-value	0.063	0.472	0.984	< .001	0.005	—									
7. TADS direct sum	Spearman's rho	0.210	0.072	-0.028	0.385	0.331	0.933	—								
	p-value	0.123	0.602	0.838	0.004	0.013	< .001	—								
8. Education	Spearman's rho	0.288	-0.283	-0.174	0.036	-0.082	-0.267	-0.186	—							
	p-value	0.033	0.036	0.204	0.794	0.551	0.049	0.174	—							
9. Relationship status	Spearman's rho	0.265	-0.300	-0.198	0.028	-0.003	0.052	-0.003	0.032	—						
	p-value	0.051	0.026	0.148	0.841	0.982	0.708	0.984	0.816	—						
10. Smoking	Spearman's rho	-0.182	0.026	0.167	-0.028	0.036	-0.010	0.033	0.000	0.162	—					
	p-value	0.183	0.848	0.222	0.839	0.792	0.944	0.813	1.000	0.239	—					
11. Semen volume	Spearman's rho	0.050	-0.097	-0.123	0.067	0.024	-0.165	-0.176	0.202	0.027	-0.112	—				
	p-value	0.717	0.483	0.370	0.626	0.860	0.229	0.198	0.140	0.843	0.415	—				
12. Sperm concentration	Spearman's rho	-0.085	-0.005	-0.095	-0.115	-0.099	-0.003	0.031	-0.224	-0.251	-0.192	-0.344	—			
	p-value	0.535	0.969	0.492	0.402	0.473	0.981	0.822	0.100	0.065	0.161	0.010	—			
13. Purified sperm conc.	Spearman's rho	-0.028	-0.102	-0.205	-0.178	-0.168	-0.074	-0.028	-0.147	-0.186	-0.261	0.009	0.866	—		
	p-value	0.838	0.458	0.133	0.193	0.220	0.590	0.840	0.283	0.173	0.054	0.951	< .001	—		
14. low / high CME	Spearman's rho	0.268	0.077	-0.045	0.393	0.429	0.870	0.864	-0.152	-0.045	-0.048	-0.169	-0.030	-0.066	—	
	p-value	0.052	0.585	0.751	0.004	0.001	< .001	< .001	0.277	0.747	0.734	0.227	0.833	0.640	—	
15. sncRNA data (yes/no)	Spearman's rho	-0.015	0.085	0.104	-0.050	-0.028	-0.072	-0.070	-0.037	-0.136	0.128	-0.036	-0.105	-0.033	-0.017	—
	p-value	0.914	0.538	0.449	0.718	0.841	0.603	0.611	0.791	0.322	0.352	0.797	0.444	0.810	0.903	—
	**Variable**	1. Age	2. BMI	3. waist circum.	4. EPDS	5. SCL	6. TADS factor sum	7. TADS direct sum	8. Education	9. Relationship status	10. Smoking	11. Semen volume	12. Sperm concentration	13. Purified sperm conc.	14. low / high CME	15. sncRNA data (yes/no)

Supplementary Table 2. Differentially expressed miRNAs in high vs. low-TADS sperm samples. Misregulated miRNAs (log2 FC < -1.0 or > 1.0 , Padj < 0.05) are indicated in red .

ID	baseMean	log2FoldChange	lfcSE	stat	pvalue	padj
hsa-miR-660-3p	3,101500135	-4,561803256	0,757346143	-6,023405941	1,71E-09	8,83E-07
hsa-miR-21-5p	6242,759839	-1,283861963	0,259004519	-4,956909505	7,16E-07	0,000156861
hsa-miR-29c-3p	75,34136418	-1,984049731	0,404073736	-4,910118015	9,10E-07	0,000156861
hsa-miR-1-3p	80,554804	3,637958996	0,751490991	4,840988171	1,29E-06	0,000166985
hsa-miR-148a-3p	66405,46094	-1,623419789	0,343919045	-4,720354432	2,35E-06	0,000224506
hsa-miR-374a-5p	14,45585974	-1,761443849	0,374799492	-4,699696471	2,61E-06	0,000224506
hsa-miR-32-5p	37,33745718	-2,058645714	0,443673089	-4,640005813	3,48E-06	0,000225153
hsa-miR-362-3p	2,005977529	-3,500327309	0,753291156	-4,646712341	3,37E-06	0,000225153
hsa-miR-582-3p	14,07731268	-2,104137361	0,457014653	-4,60409168	4,14E-06	0,000237975
hsa-miR-122-3p	2,297464358	2,888466593	0,648785708	4,452111932	8,50E-06	0,000439604
hsa-miR-141-5p	10,11577373	-1,95443766	0,452331956	-4,320803854	1,55E-05	0,000730671
hsa-miR-101-3p	144,656318	-1,291893357	0,305525138	-4,228435554	2,35E-05	0,000935857
hsa-miR-141-3p	332,4025389	-2,007651895	0,474040282	-4,235192605	2,28E-05	0,000935857
hsa-miR-323b-3p	5,639839721	2,436685164	0,581502372	4,190327131	2,79E-05	0,001028655
hsa-miR-935	3,640694944	2,352306381	0,573520936	4,101517893	4,10E-05	0,00141468
hsa-miR-19b-3p	26,28656221	-1,591603821	0,397791291	-4,001102731	6,30E-05	0,002037238
hsa-miR-433-3p	1,447163747	3,237756371	0,819227021	3,95220896	7,74E-05	0,002354877
hsa-miR-618	10,11396589	1,644713472	0,420475905	3,91155225	9,17E-05	0,002633966
hsa-miR-26b-5p	342,0698427	-1,170802511	0,308918835	-3,790000404	0,000150647	0,004099185
hsa-miR-191-5p	2085,645736	0,984691093	0,26307208	3,743046748	0,000181802	0,004670046
hsa-miR-30e-5p	89,3670748	-1,132909111	0,303536747	-3,732362304	0,000189692	0,004670046
hsa-miR-409-3p	6,772556714	1,929657327	0,524082536	3,681972198	0,000231437	0,005202293
hsa-miR-429	215,9760604	-1,343417465	0,364594951	-3,684684771	0,000228986	0,005202293
hsa-miR-25-3p	2167,158702	0,790504849	0,21877204	3,613372396	0,00030224	0,006510756
hsa-miR-323a-3p	6,289228729	1,449095331	0,405674943	3,572060236	0,000354184	0,007324523
hsa-miR-1251-5p	26,84100441	-1,683285278	0,475337722	-3,541240682	0,00039825	0,007353404
hsa-miR-134-5p	7,078655296	1,809567063	0,50858843	3,558018539	0,000373663	0,007353404
hsa-miR-452-5p	7,490226351	-1,65692756	0,467772116	-3,542168302	0,000396852	0,007353404
hsa-miR-511-5p	4,088970552	2,246977637	0,640985834	3,505502799	0,000455746	0,008124846
hsa-miR-196b-5p	78,09911468	-1,208462922	0,350415665	-3,448655536	0,000563385	0,009709
hsa-miR-200a-3p	1589,589226	-1,095093841	0,332299058	-3,295506907	0,000982443	0,016384612
hsa-miR-509-3p	29,91898882	1,045282393	0,319908794	3,267438758	0,001085253	0,017533626
hsa-miR-760	6,084416485	2,666922391	0,819950822	3,252539446	0,001143787	0,017919333
hsa-miR-3909	3,507430463	2,054956925	0,638331716	3,219261826	0,001285211	0,019542764
hsa-miR-1307-5p	9,388266432	-1,755094794	0,548421587	-3,200265697	0,00137301	0,020281312
hsa-miR-10a-5p	1838,731738	0,86096074	0,269773643	3,19141904	0,001415758	0,020331855
hsa-miR-425-5p	216,5098649	0,750887035	0,237584009	3,160511685	0,001574923	0,022006355
hsa-miR-18a-3p	1,230635881	2,763110225	0,879025117	3,143380289	0,001670087	0,022613908
hsa-miR-576-5p	6,352917315	1,808746189	0,576553859	3,13716778	0,001705885	0,022613908
hsa-miR-891b	16,63310563	-1,806623774	0,579543548	-3,117321863	0,001825022	0,02358841
hsa-miR-660-5p	9,385472118	-1,155792096	0,374526485	-3,08600898	0,002028627	0,025580491
hsa-miR-370-3p	11,56071324	1,577384524	0,51877441	3,040598176	0,002361087	0,029063857
hsa-miR-1296-5p	3,503832951	-1,348585568	0,450428473	-2,994006041	0,002753405	0,032820771
hsa-miR-548a-3p	4,699495362	-1,742312914	0,583782451	-2,984524308	0,002840196	0,032820771
hsa-miR-548ah-3p	5,034952645	-1,642592484	0,550697889	-2,982747016	0,00285674	0,032820771
hsa-miR-3615	4,95247623	2,31108492	0,782642883	2,952923958	0,003147795	0,035378478
hsa-miR-192-5p	375,4246493	-0,753822924	0,258186038	-2,919688955	0,003503809	0,038254731
hsa-miR-29b-3p	66,87896477	-1,195435528	0,410033866	-2,91545559	0,003551697	0,038254731
hsa-let-7a-3p	42,28901812	-0,926192794	0,318539223	-2,90762558	0,003641841	0,038425137
hsa-miR-518f-5p	14,06408165	-0,995343776	0,343293293	-2,899397672	0,003738804	0,038659229
hsa-miR-210-3p	11,87441739	-1,379959062	0,483793309	-2,852373186	0,004339412	0,042975901
hsa-miR-27a-3p	1679,878335	-0,65981354	0,231896064	-2,845298574	0,004436981	0,042975901
hsa-miR-518d-5p	14,22796053	-0,963104035	0,340746975	-2,826449259	0,00470672	0,042975901
hsa-miR-520c-5p	14,22796053	-0,963104035	0,340746975	-2,826449259	0,00470672	0,042975901
hsa-miR-526a-5p	14,22796053	-0,963104035	0,340746975	-2,826449259	0,00470672	0,042975901
hsa-miR-532-3p	10,23563794	1,332892456	0,46776186	2,849510769	0,004378652	0,042975901
hsa-miR-888-5p	310,5369553	-1,363309692	0,482704271	-2,82431661	0,004738155	0,042975901
hsa-miR-34c-5p	992,3372246	-1,237862119	0,441936743	-2,800993897	0,005094548	0,045411749
hsa-miR-340-5p	87,22835812	-0,943285159	0,342196918	-2,75655656	0,005841351	0,051186075
hsa-miR-96-5p	90,62132264	-0,893796467	0,325888634	-2,74264388	0,006094674	0,052515777
hsa-miR-106b-5p	12,08413295	-1,057953729	0,38736356	-2,731164824	0,00631109	0,052948239
hsa-miR-146b-3p	24,54244475	0,977370125	0,35812193	2,729154633	0,006349692	0,052948239
hsa-miR-582-5p	2,982825049	-1,730074578	0,635569288	-2,722086502	0,006487116	0,053235539
hsa-miR-224-5p	49,36196913	0,918211039	0,343718395	2,671404997	0,007553445	0,06007894
hsa-miR-651-5p	14,27174221	-1,347683978	0,504175749	-2,673044031	0,007516638	0,06007894
hsa-miR-28-3p	405,9676426	0,541881001	0,204406934	2,650991287	0,008025591	0,062240056
hsa-miR-361-3p	67,14226276	0,823473981	0,31082728	2,649297643	0,008065926	0,062240056
hsa-miR-5683	3,77232814	-1,568452018	0,59560714	-2,633366715	0,008454301	0,064277554
hsa-miR-23a-5p	4,339177242	1,67495828	0,637923998	2,625639237	0,008648643	0,06480215
hsa-miR-215-5p	240,3175641	-1,306381306	0,499455155	-2,615612819	0,008906749	0,065782704
hsa-miR-34c-3p	100,8018155	0,847786161	0,324864352	2,609662021	0,009063172	0,065995207
hsa-miR-30d-3p	5,29000306	-1,123189451	0,433932232	-2,588398299	0,009642342	0,069237369
hsa-miR-335-5p	49,76327618	-1,200338926	0,465236291	-2,580062968	0,00987823	0,06995952
hsa-miR-10b-3p	103,6973378	-0,913864535	0,355667092	-2,56943798	0,010186362	0,070217991
hsa-miR-890	5,089700297	-1,428499996	0,55505105	-2,573637138	0,010063576	0,070217991
hsa-miR-664a-3p	14,93475535	0,83278055	0,328934876	2,531749023	0,01134952	0,0772066
hsa-miR-136-5p	1,700304049	-1,603518999	0,637584589	-2,514990209	0,011903569	0,078899297
hsa-miR-449b-3p	2,667898789	1,44941639	0,576052419	2,516118917	0,011865514	0,078899297
hsa-miR-339-5p	17,46580691	1,562413124	0,625674452	2,497166249	0,012519025	0,080904199
hsa-miR-518a-3p	11,30511167	1,036408977	0,414531117	2,50019585	0,012412466	0,080904199
hsa-miR-140-5p	13,2806734	-1,139313403	0,460259131	-2,475373817	0,013309678	0,084951898
hsa-miR-342-3p	70,58920831	0,755886175	0,306333374	2,467527991	0,013604958	0,085777604
hsa-miR-142-5p	4,184102557	-1,868755639	0,760451313	-2,457429697	0,01399352	0,087164454
hsa-miR-15a-5p	7,52598749	-1,098748256	0,449680966	-2,443395072	0,0145498	0,089550554
hsa-miR-105-5p	22,97118482	0,838994799	0,344584494	2,434801372	0,014899966	0,090626854
hsa-miR-212-5p	25,31840727	0,967113238	0,398052191	2,429614158	0,015114904	0,090865179
hsa-miR-22-5p	464,4652923	-0,725498598	0,30235685	-2,399477963	0,016418468	0,097567218
hsa-miR-221-5p	3,934578339	-1,29178499	0,54061554	-2,389470693	0,01687267	0,09801315
hsa-miR-664a-5p	60,39987519	0,588418734	0,245838593	2,39351652	0,01668773	0,09801315
hsa-miR-140-3p	153,8536817	-0,655343893	0,274937107	-2,383613838	0,017143581	0,09848035
hsa-let-7f-2-3p	1,366397113	-1,834091878	0,785166166	-2,335928312	0,019494978	0,107069977
hsa-miR-130a-3p	21,09530871	-0,888601075	0,379773586	-2,339818009	0,019293139	0,107069977
hsa-miR-3127-5p	1,663095961	1,811376382	0,773002743	2,343298778	0,01911407	0,107069977
hsa-miR-561-5p	3,20379679	-1,397856863	0,597670001	-2,338843944	0,019343511	0,107069977
hsa-miR-6842-3p	2,912303069	1,283054837	0,55007694	2,332500682	0,019674367	0,107069977
hsa-miR-139-5p	95,63017697	0,817672282	0,35339744	2,313747046	0,020681602	0,111379042
hsa-miR-125a-3p	26,21930424	0,967041457	0,420325823	2,300694851	0,021408885	0,114107152
hsa-miR-34b-3p	171,1054568	0,902819185	0,394077318	2,290969677	0,021965168	0,115877465
hsa-miR-107	165,1738663	-0,885752159	0,387312717	-2,286917316	0,02220065	0,115936726
hsa-miR-2110	87,0631655	0,686600881	0,305858702	2,244830303	0,024779029	0,125595665
hsa-miR-34b-5p	1,892326073	-1,54036946	0,685685048	-2,246467914	0,024674052	0,125595665
hsa-miR-432-5p	8,113502143	1,428256789	0,634934247	2,249456218	0,024483485	0,125595665
hsa-miR-106b-3p	106,1208799	0,579281509	0,258970741	2,236860836	0,025295437	0,126968358
hsa-miR-1271-5p	4,782249168	1,039840688	0,471732761	2,204300346	0,027503229	0,135420659
hsa-miR-147b-3p	9,171149279	-1,014944015	0,459923046	-2,206769205	0,027330188	0,135420659
hsa-let-7d-5p	130,9221732	0,665424438	0,30358243	2,191906947	0,028386226	0,137155879
hsa-miR-525-5p	8,180293354	0,829029914	0,377726713	2,194787625	0,028178836	0,137155879
hsa-miR-28-5p	105,1590167	0,628766319	0,290356993	2,165493976	0,030349876	0,145285982
hsa-miR-495-3p	46,67884523	0,673980148	0,315992943	2,132896206	0,032933242	0,154930194
hsa-miR-6510-3p	1,877684618	1,62905222	0,763908374	2,132523057	0,032963871	0,154930194
hsa-miR-30c-5p	519,5359521	0,816738285	0,384001958	2,126911774	0,033427414	0,155693453
hsa-miR-193a-3p	3,189035464	-1,521350565	0,717363516	-2,12075263	0,033942625	0,156681581
hsa-miR-3925-3p	13,77844844	0,97760665	0,463183663	2,110624202	0,034804626	0,157842031
hsa-miR-92b-5p	18,52442552	1,086379016	0,513927075	2,113877763	0,034525709	0,157842031
hsa-miR-20b-5p	21,79560981	-0,86383409	0,414763399	-2,082715333	0,037277179	0,163324589
hsa-miR-338-5p	12,67485407	0,942420452	0,45235721	2,08335455	0,037218918	0,163324589
hsa-miR-598-3p	20,56829073	-0,7097897	0,339716518	-2,08935881	0,036675436	0,163324589
hsa-miR-9-3p	107,297806	-0,678805588	0,325682563	-2,084255239	0,037136957	0,163324589
hsa-miR-548aq-3p	1,909237707	-1,758414391	0,847290181	-2,075339041	0,037955124	0,16489747
hsa-miR-153-3p	4,888381077	-1,315538532	0,639195941	-2,058114652	0,039579129	0,17052008
hsa-miR-103a-3p	306,3504458	-0,538069551	0,262965572	-2,046159681	0,040740663	0,174073741
hsa-miR-519a-3p	5,509115669	-1,156638677	0,569223066	-2,031960308	0,042157674	0,178651784
hsa-miR-17-5p	20,9858207	-0,589424836	0,290886007	-2,026308664	0,042733164	0,179618258
hsa-miR-15b-5p	101,6947004	0,584873271	0,289344401	2,021374074	0,043241056	0,180287308
hsa-miR-1180-3p	79,99197629	0,640473718	0,318024222	2,013914898	0,044018469	0,180615465
hsa-miR-523-3p	1,513552191	1,611929452	0,800386727	2,013938261	0,044016016	0,180615465
hsa-miR-450a-5p	8,222030858	-1,142885791	0,568499944	-2,01035339	0,0443938	0,180721217
hsa-miR-200a-5p	136,7984878	-0,565697172	0,283216276	-1,997403471	0,045781371	0,184913819
hsa-miR-328-3p	29,43383511	1,080647092	0,542706353	1,991218799	0,046456838	0,186187483
hsa-miR-216b-5p	6,159288664	0,860430571	0,434352573	1,980949637	0,047596922	0,189289297
hsa-miR-136-3p	5,302667857	-0,834339425	0,422830762	-1,973223093	0,048470151	0,191290594
hsa-miR-150-5p	28,33453443	1,23465314	0,629053534	1,96271553	0,049679238	0,194577014
hsa-miR-4683	1,97696523	1,994676176	1,024218135	1,94751109	0,051473492	0,200088685
hsa-miR-885-5p	1,246175384	1,483229251	0,776996994	1,90892534	0,056271724	0,217108068
hsa-miR-1301-3p	6,645121529	1,119625814	0,594841649	1,882224984	0,059805475	0,220048483
hsa-miR-24-3p	1139,362188	-0,508267073	0,2707035	-1,877578507	0,060438848	0,220048483
hsa-miR-296-5p	16,85567618	1,044059307	0,554445235	1,883070212	0,059690853	0,220048483
hsa-miR-517a-3p	83,06100193	-0,623175371	0,33188484	-1,877685556	0,060424194	0,220048483
hsa-miR-517b-3p	83,06100193	-0,623175371	0,33188484	-1,877685556	0,060424194	0,220048483
hsa-miR-766-3p	2,93179895	1,441607188	0,765442668	1,88336403	0,059651052	0,220048483
hsa-miR-892c-5p	2,451661383	-1,241544519	0,657800481	-1,887418077	0,059104116	0,220048483
hsa-miR-9-5p	195,0398382	0,698073609	0,370109325	1,886128129	0,059277691	0,220048483
hsa-miR-3150b-3p	3,523027441	0,846532001	0,45603594	1,856283523	0,063413156	0,22926295
hsa-miR-152-3p	198,9099248	-0,543146082	0,29522824	-1,839749755	0,065804985	0,232387017
hsa-miR-181d-5p	16,36610834	-0,660587828	0,359933736	-1,835304009	0,066460657	0,232387017
hsa-miR-363-3p	1214,334054	-0,632174107	0,344086155	-1,837255285	0,066172218	0,232387017
hsa-miR-485-5p	5,952553784	0,980108842	0,531636853	1,843568286	0,065246081	0,232387017
hsa-miR-517c-3p	20,92661269	-0,799650586	0,435807381	-1,8348716	0,066524717	0,232387017
hsa-miR-365a-3p	6,923263431	1,067826919	0,588272507	1,815190928	0,069494573	0,237938374
hsa-miR-365b-3p	6,923263431	1,067826919	0,588272507	1,815190928	0,069494573	0,237938374
hsa-miR-516b-5p	908,5242683	-0,66261566	0,364427961	-1,818234963	0,069028228	0,237938374
hsa-miR-139-3p	2,678957244	1,10419418	0,613257075	1,800540466	0,07177534	0,243573512
hsa-miR-6715b-3p	3,941022711	0,886905405	0,493109977	1,798595539	0,072082684	0,243573512
hsa-miR-132-3p	326,3539197	0,588501294	0,328708756	1,790342617	0,073398849	0,244820677
hsa-miR-217-5p	1,460427415	-2,048260638	1,142437225	-1,792886815	0,072991024	0,244820677
hsa-miR-19a-3p	1,917938778	-1,107363403	0,621688364	-1,781219443	0,074876608	0,246568195
hsa-miR-425-3p	9,455630081	-0,738190706	0,414338811	-1,781611299	0,074812641	0,246568195
hsa-miR-10b-5p	1823,693427	0,530500754	0,298966969	1,77444604	0,0759894	0,246952535
hsa-miR-128-3p	336,3066441	0,669857366	0,378009561	1,772064614	0,076383832	0,246952535
hsa-miR-7154-5p	3,738687537	-1,01602231	0,573437925	-1,771808708	0,076426317	0,246952535
hsa-miR-548b-5p	4,809590202	0,836001399	0,478208585	1,748194042	0,080430433	0,258276608
hsa-miR-3158-3p	74,04398207	0,58364683	0,336257442	1,735714241	0,082614374	0,263652046
hsa-miR-487b-3p	5,114742019	0,802393126	0,464297882	1,728186056	0,083954873	0,264662618
hsa-miR-502-3p	98,91174179	-0,49543254	0,286544098	-1,728992304	0,083810472	0,264662618
hsa-miR-129-5p	20,11502228	0,606681591	0,353448291	1,716464914	0,086076984	0,268060447
hsa-miR-493-5p	6,830456558	0,605421934	0,353254708	1,713839673	0,086558175	0,268060447
hsa-miR-654-3p	2,349143961	1,172950763	0,684464597	1,713676307	0,086588191	0,268060447
hsa-miR-873-5p	51,07500791	-0,612809632	0,358568252	-1,709045986	0,087442432	0,269093673
hsa-miR-193b-3p	53,54544367	0,796833901	0,472237091	1,687359838	0,091534182	0,280018768
hsa-miR-182-3p	1,347531495	-1,247849458	0,747014499	-1,670448779	0,094830606	0,288396607
hsa-miR-1468-5p	2,764737847	1,270186883	0,764476802	1,661511349	0,096610792	0,292092276
hsa-miR-548e-3p	3,82521139	-0,851844036	0,51356387	-1,658691519	0,097177969	0,292098896
hsa-miR-7706	9,066401995	0,946224336	0,575879933	1,643093085	0,100363675	0,299930753
hsa-miR-99b-5p	574,2579525	0,628131106	0,384196854	1,634919962	0,102065827	0,303264556
hsa-miR-377-3p	1,352298086	-1,167385212	0,724161595	-1,612050707	0,106950906	0,314168286
hsa-miR-497-5p	1,803062205	-0,986910787	0,611357401	-1,614294333	0,106463599	0,314168286
hsa-miR-656-3p	1,79497037	1,2738494	0,791685741	1,609034159	0,107608873	0,314315182
hsa-miR-29a-3p	932,3133094	-0,552917846	0,346280755	-1,596732817	0,110325277	0,318648985
hsa-miR-92a-1-5p	2,259052552	1,236111594	0,773392279	1,598298339	0,109976597	0,318648985
hsa-miR-335-3p	86,64570445	0,69730945	0,439930882	1,585043192	0,112956521	0,322643765
hsa-miR-340-3p	8,824546111	0,803834768	0,506564934	1,586834608	0,112550108	0,322643765
hsa-miR-1323	160,971208	0,419993274	0,267641335	1,569239197	0,116592214	0,328682674
hsa-miR-4773	4,738898286	-0,766203332	0,487103183	-1,572979522	0,11572356	0,328682674
hsa-miR-7-5p	211,6660022	-0,583517097	0,372239483	-1,56758518	0,116977973	0,328682674
hsa-miR-106a-5p	14,53083098	-0,731232585	0,470653637	-1,55365332	0,120267133	0,333826397
hsa-miR-1185-1-3p	1,180530512	-1,208099936	0,778589916	-1,551651146	0,120745718	0,333826397
hsa-miR-204-5p	28,62968731	0,959338693	0,617301839	1,554083645	0,120164466	0,333826397
hsa-miR-26b-3p	1,743441848	1,044377298	0,678255509	1,539799212	0,123609304	0,339925586
hsa-miR-29c-5p	12,14843686	0,508792939	0,335286346	1,517487799	0,129143572	0,349566631
hsa-miR-4700-5p	3,878676991	0,826756192	0,544222138	1,51915208	0,12872422	0,349566631
hsa-miR-625-5p	45,46001315	0,600098419	0,394177506	1,522406557	0,127907243	0,349566631
hsa-miR-21-3p	2,21297427	-0,992372906	0,659976886	-1,503647972	0,132672032	0,35609687
hsa-miR-223-3p	160,1104362	0,87996889	0,58561788	1,502633236	0,132933648	0,35609687
hsa-miR-3613-5p	12,75029402	-0,753836153	0,503943269	-1,49587503	0,134686224	0,358931845
hsa-miR-423-3p	1225,688223	0,619544984	0,416415074	1,487806334	0,136801975	0,36270062
hsa-miR-3130-3p	1,876036839	-1,394827039	0,944353975	-1,477017174	0,139671034	0,367728843
hsa-miR-532-5p	156,6157197	-0,296023493	0,200647587	-1,475340406	0,140121048	0,367728843
hsa-miR-200b-5p	90,7488463	0,614202464	0,421801161	1,456142184	0,145353318	0,379533664
hsa-miR-372-3p	246,0259068	-0,510302857	0,35111857	-1,453363338	0,1461229	0,379625826
hsa-miR-151a-5p	16,42789741	0,463549122	0,321407702	1,442246468	0,149232856	0,384721034
hsa-miR-504-5p	3,103156599	0,838607367	0,58194452	1,441043499	0,149572394	0,384721034
hsa-miR-30a-5p	1998,992092	-0,440960916	0,30763472	-1,43339125	0,151746058	0,38646656
hsa-miR-767-5p	4,084389331	0,716981684	0,499724131	1,434754977	0,151356932	0,38646656
hsa-miR-125b-5p	361,0643288	0,771218982	0,541291607	1,424775428	0,154222127	0,390847254
hsa-miR-1295a	11,52012987	0,598151781	0,421857248	1,417900922	0,156219693	0,39273224
hsa-miR-409-5p	2,180176533	1,0479524	0,740917167	1,414398865	0,157244823	0,39273224
hsa-miR-4661-5p	17,91777718	0,61910132	0,43701677	1,416653463	0,156584269	0,39273224
hsa-miR-199b-5p	11,05129338	-0,583133679	0,420147454	-1,387926248	0,165159504	0,410516652
hsa-miR-30b-3p	7,458186567	0,686961681	0,497419363	1,381051346	0,167263174	0,412197754
hsa-miR-515-5p	6,548393711	-0,818819842	0,593129559	-1,380507564	0,167430422	0,412197754
hsa-miR-1843	21,87180148	0,515886899	0,3756782	1,373214892	0,169685551	0,41472671
hsa-miR-196a-5p	23,91561792	0,588047115	0,428604343	1,372004566	0,170062016	0,41472671
hsa-miR-181a-5p	128,3565535	0,454165626	0,333614573	1,361348283	0,173403651	0,41495283
hsa-miR-320d	12,38142504	0,653385724	0,483788272	1,350561314	0,176836	0,41495283
hsa-miR-324-3p	6,608342343	0,712836612	0,528841352	1,347921469	0,177683642	0,41495283
hsa-miR-382-5p	39,93126188	0,353599175	0,261363986	1,352899379	0,176087778	0,41495283
hsa-miR-484	64,43501217	0,655944712	0,484135248	1,354879065	0,175456091	0,41495283
hsa-miR-505-5p	2,878625439	1,052174065	0,780305277	1,348413367	0,177525467	0,41495283
hsa-miR-520a-5p	37,73412149	-0,464540309	0,345029844	-1,346377183	0,178180906	0,41495283
hsa-miR-6866-5p	1,265760162	-0,909004133	0,673433857	-1,349804622	0,177078662	0,41495283
hsa-miR-941	87,81422352	0,529805087	0,389169519	1,361373543	0,173395673	0,41495283
hsa-miR-98-5p	55,32161365	-0,516608135	0,381913004	-1,352685376	0,176156164	0,41495283
hsa-let-7i-5p	1190,399261	-0,428190357	0,326489368	-1,311498621	0,189689359	0,437809816
hsa-miR-518f-3p	1,421813237	-1,051520235	0,801051652	-1,312674698	0,189292587	0,437809816
hsa-miR-1299	12,99593084	-1,070258966	0,819422524	-1,306113677	0,191513903	0,440056389
hsa-miR-378a-5p	2,684427281	0,791159908	0,607470092	1,302384953	0,192784824	0,44101661
hsa-miR-330-3p	3,558420591	0,670666168	0,518089813	1,294497887	0,195493499	0,444532864
hsa-miR-381-3p	94,28273488	-0,400249163	0,309571905	-1,29291178	0,196041573	0,444532864
hsa-miR-15b-3p	17,67339449	-0,458097273	0,355847114	-1,28734295	0,19797478	0,446956162
hsa-miR-20a-5p	32,21296686	-0,452432085	0,353055009	-1,281477597	0,200025964	0,447677157
hsa-miR-371a-3p	17,66033772	0,363758305	0,283496075	1,283115841	0,199451495	0,447677157
hsa-miR-149-5p	243,8875132	0,791249874	0,621561632	1,273003084	0,203016948	0,452412767
hsa-miR-27b-3p	1310,4988	-0,272001521	0,214208173	-1,269799924	0,204155908	0,452998302
hsa-miR-193a-5p	298,5790299	0,486389524	0,384701542	1,264329543	0,20611177	0,453445894
hsa-miR-2113	1,566347943	0,824621492	0,652092086	1,264578286	0,206022541	0,453445894
hsa-let-7d-3p	115,4718714	0,510439536	0,406776257	1,254841026	0,209536476	0,456117403
hsa-miR-197-3p	15,47161002	-0,572200008	0,45618659	-1,254311329	0,209728868	0,456117403
hsa-miR-411-5p	5,711111996	-0,450933286	0,359699124	-1,253640212	0,209972808	0,456117403
hsa-miR-10527-5p	4,028579	0,529545184	0,425553252	1,244368788	0,213363865	0,459621325
hsa-miR-195-5p	21,12660705	-0,406345043	0,326160198	-1,245844973	0,212821315	0,459621325
hsa-miR-127-3p	36,7198824	0,466216872	0,377086802	1,236364863	0,216322971	0,460259615
hsa-miR-142-3p	12,05444266	-0,788121781	0,640426245	-1,23062068	0,218464767	0,460259615
hsa-miR-219a-1-3p	1,336677427	0,836674416	0,680672969	1,229187076	0,219001674	0,460259615
hsa-miR-410-3p	2,958803295	0,736982876	0,595988289	1,236572747	0,216245743	0,460259615
hsa-miR-524-5p	3,422266049	-0,725477458	0,589183969	-1,231325861	0,218201013	0,460259615
hsa-miR-543	16,73998557	0,700082655	0,568916015	1,230555366	0,218489208	0,460259615
hsa-miR-218-5p	262,2989308	-0,497062337	0,406959263	-1,221405636	0,221932474	0,462657618
hsa-miR-382-3p	2,929005787	0,651650514	0,532994773	1,222620836	0,22147294	0,462657618
hsa-miR-30a-3p	630,7158654	0,271037366	0,224218431	1,208809487	0,226736046	0,468890143
hsa-miR-548o-3p	31,20445066	-0,479316409	0,396214488	-1,209739734	0,226378778	0,468890143
hsa-miR-26a-5p	3335,250978	-0,276159355	0,229349396	-1,204098898	0,228551356	0,470761159
hsa-miR-892a	67,72118857	0,395994714	0,335023038	1,181992487	0,237208682	0,48665432
hsa-miR-424-5p	2,452381502	-0,785435768	0,674147907	-1,165079294	0,243986897	0,498581921
hsa-let-7e-3p	2,081425828	0,843922792	0,743838782	1,134550676	0,25656363	0,502940354
hsa-miR-126-3p	249,6748011	0,504234242	0,439108463	1,148313649	0,250839107	0,502940354
hsa-miR-1269a	1,415053141	-1,931493194	1,704130263	-1,133418751	0,257038443	0,502940354
hsa-miR-1283	23,02258585	-0,407937925	0,358102028	-1,139166756	0,254633609	0,502940354
hsa-miR-181c-5p	5,804471116	-0,583800164	0,514266115	-1,135210249	0,256287236	0,502940354
hsa-miR-199a-5p	19,89404483	0,57738725	0,511380439	1,129075746	0,258865882	0,502940354
hsa-miR-200b-3p	895,4218558	-0,311504254	0,274224548	-1,135945911	0,255979202	0,502940354
hsa-miR-214-3p	3,119916943	0,93601716	0,815520422	1,147754409	0,251069963	0,502940354
hsa-miR-30e-3p	170,309093	-0,252492787	0,220682124	-1,144146984	0,252562686	0,502940354
hsa-miR-329-3p	3,03817015	0,866470545	0,768823652	1,127008181	0,259739022	0,502940354
hsa-miR-4446-3p	1,346020996	0,970580708	0,841654433	1,15318196	0,248835707	0,502940354
hsa-miR-486-5p	157,7280846	0,462386631	0,406328985	1,13796123	0,255136672	0,502940354
hsa-miR-514a-3p	381,6855966	-0,43780232	0,388423761	-1,127125485	0,25968943	0,502940354
hsa-miR-942-5p	4,530153975	0,786464228	0,682651702	1,152072463	0,249291298	0,502940354
hsa-miR-151a-3p	1412,132245	-0,286392781	0,255719843	-1,119947427	0,262736166	0,506233369
hsa-miR-320a-3p	5093,776305	0,394739282	0,352951351	1,118395724	0,26339802	0,506233369
hsa-miR-1255b-5p	1,983166939	-0,651480353	0,58504912	-1,113548131	0,265473089	0,508331804
hsa-miR-518c-3p	3,168736469	-0,684820717	0,623129293	-1,099002605	0,271766929	0,518463109
hsa-miR-574-3p	108,1937317	0,612554858	0,56267816	1,088641609	0,27631196	0,525195895
hsa-miR-499a-5p	1201,801141	-0,51220762	0,471559625	-1,086199056	0,277390935	0,525315433
hsa-miR-331-5p	5,525314348	-0,556685016	0,518291071	-1,074077959	0,282787742	0,531640955
hsa-miR-7159-5p	1,200675585	-0,798204957	0,741688803	-1,076199282	0,281838141	0,531640955
hsa-miR-521	8,911166162	-0,486815164	0,457822031	-1,063328391	0,287633054	0,538790903
hsa-miR-769-5p	23,99234225	-0,278153639	0,263123744	-1,057121012	0,290456363	0,542115305
hsa-miR-526b-5p	6,028260837	0,456635819	0,433632124	1,053048873	0,292318593	0,543628463
hsa-miR-185-5p	40,58021869	-0,28302438	0,269546632	-1,050001544	0,293717403	0,544272033
hsa-miR-146b-5p	631,7214183	0,342085639	0,327926482	1,043177841	0,296865934	0,54619106
hsa-miR-378d	3,373255367	0,541073616	0,518463778	1,043609291	0,296666192	0,54619106
hsa-miR-195-3p	6,073200906	0,496904136	0,478761365	1,037895227	0,299318836	0,548751199
hsa-miR-23b-5p	2,629315811	0,660014102	0,640973279	1,029706111	0,303147986	0,551857425
hsa-miR-483-5p	3,078304767	0,824289709	0,800060846	1,030283775	0,302876813	0,551857425
hsa-miR-182-5p	590,7430869	0,294529166	0,28675314	1,027117491	0,304365143	0,55212905
hsa-miR-22-3p	5253,314482	0,314292444	0,311551614	1,008797354	0,31307183	0,563965632
hsa-miR-30d-5p	1437,739673	0,208210987	0,2062847	1,009338003	0,312812561	0,563965632
hsa-miR-374b-5p	78,90547347	-0,272761038	0,275531419	-0,989945318	0,322200847	0,578395271
hsa-miR-23a-3p	575,2941916	0,261508657	0,267835077	0,976379419	0,328876477	0,588336119
hsa-let-7b-3p	21,85739045	0,44520734	0,461865162	0,963933581	0,335079226	0,591500158
hsa-miR-181a-2-3p	5,63340812	-0,652166067	0,677979626	-0,96192576	0,336086898	0,591500158
hsa-miR-205-5p	84,99316944	0,374287869	0,38932719	0,961370999	0,336365661	0,591500158
hsa-miR-615-3p	3,225768357	-0,862585042	0,897163658	-0,961457851	0,336322009	0,591500158
hsa-miR-6516-5p	2,94340056	0,514321699	0,532058599	0,966663635	0,333712212	0,591500158
hsa-miR-125a-5p	235,6744817	0,553971319	0,579877339	0,955324999	0,339413369	0,594836311
hsa-miR-766-5p	2,398064896	-0,808400394	0,849908934	-0,951161192	0,341522556	0,59651068
hsa-miR-708-3p	16,17729797	-0,296944645	0,313803064	-0,946277072	0,344007285	0,598827496
hsa-miR-135a-5p	69,08124798	-0,34807954	0,370561993	-0,939328768	0,347561973	0,600574728
hsa-miR-3925-5p	13,39941952	-0,382484044	0,407978367	-0,937510601	0,348495974	0,600574728
hsa-miR-92a-3p	1196,237161	0,374316228	0,397752695	0,941077791	0,346664996	0,600574728
hsa-miR-148b-3p	382,2126003	-0,225719276	0,244452336	-0,923367228	0,355815864	0,608072275
hsa-miR-320b	1545,892031	0,342873925	0,371761917	0,922294376	0,356375047	0,608072275
hsa-miR-3940-5p	1,724732684	0,979488418	1,058317721	0,925514521	0,354698334	0,608072275
hsa-miR-206	4,682205262	-0,590736897	0,643917784	-0,91741044	0,358927604	0,610059903
hsa-miR-3131	2,94557682	-0,66884029	0,730529278	-0,915555762	0,359899943	0,610059903
hsa-miR-362-5p	2,209124698	-0,685940834	0,751061648	-0,913294981	0,361087421	0,610072538
hsa-let-7c-5p	1574,902396	-0,269773311	0,297096483	-0,908032665	0,363860964	0,612756086
hsa-miR-181a-3p	3,722075704	0,593770295	0,65828423	0,901996839	0,367058547	0,616133989
hsa-miR-16-2-3p	42,18832929	0,330522133	0,369786208	0,893819526	0,37141846	0,62143477
hsa-miR-548k	4,226278382	-0,514503333	0,579637689	-0,887629191	0,374740246	0,624970023
hsa-miR-518e-5p	44,04056172	-0,251439016	0,288880436	-0,870391292	0,384086604	0,628394855
hsa-miR-519a-5p	44,04056172	-0,251439016	0,288880436	-0,870391292	0,384086604	0,628394855
hsa-miR-519b-5p	44,04056172	-0,251439016	0,288880436	-0,870391292	0,384086604	0,628394855
hsa-miR-519c-5p	44,04056172	-0,251439016	0,288880436	-0,870391292	0,384086604	0,628394855
hsa-miR-522-5p	44,04056172	-0,251439016	0,288880436	-0,870391292	0,384086604	0,628394855
hsa-miR-523-5p	44,04056172	-0,251439016	0,288880436	-0,870391292	0,384086604	0,628394855
hsa-miR-371a-5p	3,87051197	-0,472919862	0,546041774	-0,86608733	0,386442273	0,630254432
hsa-miR-30c-1-3p	3,214594035	0,480682346	0,557984641	0,861461608	0,388983857	0,632404573
hsa-miR-518e-3p	55,62040537	0,282754087	0,330119546	0,856520282	0,391710059	0,63484044
hsa-miR-143-5p	2,7569893	-1,02115777	1,196501922	-0,853452678	0,393408319	0,635600316
hsa-let-7g-5p	1314,607134	-0,253528227	0,299214272	-0,847313284	0,396820524	0,636067716
hsa-miR-34a-5p	3,120862607	-0,658005708	0,777525443	-0,846281898	0,397395504	0,636067716
hsa-miR-3605-5p	1,715941514	-0,517656645	0,612973839	-0,844500388	0,398389846	0,636067716
hsa-miR-4800-5p	1,597480574	-0,661718041	0,783942061	-0,844090493	0,39861884	0,636067716
hsa-miR-619-5p	6,002743263	0,444792693	0,532634345	0,83508076	0,403672239	0,642149377
hsa-let-7f-5p	927,7155945	-0,259724528	0,317928362	-0,816927834	0,41396967	0,644645541
hsa-miR-1278	7,495739686	-0,347405295	0,422370476	-0,822513209	0,410784872	0,644645541
hsa-miR-191-3p	3,235389124	0,543582294	0,657083584	0,827265066	0,40808684	0,644645541
hsa-miR-320c	318,8483202	0,313640088	0,378916866	0,827727969	0,407824576	0,644645541
hsa-miR-513c-5p	16,39085099	0,324836587	0,395170022	0,822017281	0,411067062	0,644645541
hsa-miR-518c-5p	8,951984738	-0,342661437	0,417612983	-0,820523907	0,411917506	0,644645541
hsa-miR-99a-5p	28268,04835	0,262278529	0,32062181	0,81803084	0,413339578	0,644645541
hsa-miR-592	19,47027882	-0,456852587	0,562473038	-0,812221309	0,416664654	0,646893773
hsa-miR-508-3p	25,31148154	0,31917415	0,39608976	0,805812676	0,420350868	0,65066287
hsa-miR-365b-5p	1,478261357	0,673611633	0,843335398	0,798747016	0,424437119	0,655026837
hsa-miR-32-3p	5,334547585	-0,339457549	0,430830443	-0,78791449	0,430746725	0,659628909
hsa-miR-372-5p	1,735559089	0,603935632	0,771597464	0,782708161	0,433798509	0,659628909
hsa-miR-424-3p	10,09066729	0,350639498	0,447511043	0,783532616	0,433314408	0,659628909
hsa-miR-4662a-5p	2,4991863	0,418769659	0,532529854	0,786377809	0,43164618	0,659628909
hsa-miR-9903	1,951745715	-0,528366723	0,669505042	-0,789190059	0,430000931	0,659628909
hsa-miR-412-5p	5,152980308	0,533141623	0,688591051	0,774250003	0,438782964	0,663410289
hsa-miR-503-5p	1,838369103	-0,459447237	0,595040631	-0,772127503	0,440038914	0,663410289
hsa-miR-550a-5p	10,96949365	-0,312587836	0,404924629	-0,771965481	0,440134872	0,663410289
hsa-miR-874-3p	8,98393257	-0,332111803	0,43201607	-0,768748724	0,442042494	0,664348748
hsa-miR-130b-3p	41,81737309	-0,281320314	0,369575856	-0,761197761	0,446538955	0,667227283
hsa-miR-4667-5p	1,794563942	-0,630316429	0,827850864	-0,761388864	0,446424837	0,667227283
hsa-miR-23b-3p	83,78833125	0,265193254	0,36639767	0,723785319	0,469197544	0,699063775
hsa-miR-423-5p	8946,538905	0,291285912	0,40434701	0,720385968	0,471287387	0,70015971
hsa-miR-516a-5p	82,03491791	-0,301039833	0,422951344	-0,711759962	0,47661343	0,705300736
hsa-miR-744-5p	93,77279256	-0,23296329	0,327947599	-0,710367422	0,47747632	0,705300736
hsa-miR-1248	1,311123108	0,542995527	0,779725762	0,696392954	0,486182774	0,710046594
hsa-miR-421	6,907751226	-0,284134174	0,40563671	-0,700464646	0,483637177	0,710046594
hsa-miR-4746-5p	1,88713513	-0,498962298	0,711186422	-0,701591429	0,482933998	0,710046594
hsa-miR-625-3p	46,69939455	-0,310350215	0,445019535	-0,697385599	0,485561511	0,710046594
hsa-miR-3679-5p	1,34400312	0,540231968	0,784858113	0,688318002	0,491252548	0,713429026
hsa-miR-628-3p	3,666428084	0,292549599	0,425026996	0,688308276	0,491258671	0,713429026
hsa-miR-1306-5p	1,459465837	-0,575150321	0,838828973	-0,685658626	0,492928403	0,71380175
hsa-miR-3913-5p	5,711299731	0,310243388	0,453888969	0,683522644	0,494276647	0,71380175
hsa-miR-520c-3p	1,614706661	0,50575846	0,74546129	0,678450332	0,497486205	0,716435566
hsa-miR-494-3p	1,667040451	-0,520340564	0,783994767	-0,663704128	0,506879697	0,727935564
hsa-miR-664b-5p	2,985587864	-0,42892709	0,654028299	-0,655823441	0,511937732	0,733162902
hsa-miR-125b-2-3p	11,94056828	-0,251483791	0,39082737	-0,643465145	0,519922324	0,736574157
hsa-miR-223-5p	8,175341741	-0,453872434	0,705519236	-0,64331868	0,520018505	0,736574157
hsa-miR-514a-5p	1,885481619	-0,453144299	0,701604736	-0,645868357	0,518364614	0,736574157
hsa-miR-652-3p	52,06812535	-0,269349243	0,416696907	-0,64639127	0,518025993	0,736574157
hsa-miR-487a-3p	2,399903438	-0,39189387	0,616298504	-0,635883209	0,524852548	0,739369938
hsa-miR-520h	6,778427676	0,301626797	0,473349688	0,637217695	0,523983053	0,739369938
hsa-miR-339-3p	59,74466219	-0,16740897	0,264772347	-0,632275129	0,527207113	0,740668688
hsa-miR-181b-5p	26,72872287	0,219083477	0,350394772	0,625247564	0,531808589	0,745108511
hsa-miR-146a-5p	626,1356175	-0,246542405	0,404414941	-0,609627341	0,542108696	0,755445272
hsa-miR-147b-5p	1,792575084	-0,491279324	0,804202027	-0,610890432	0,54127212	0,755445272
hsa-miR-135b-5p	8,519253951	-0,282362817	0,473247005	-0,596649982	0,550741094	0,763934535
hsa-miR-199a-3p	45,8434202	-0,227117993	0,382470369	-0,593818531	0,552633493	0,763934535
hsa-miR-199b-3p	45,8434202	-0,227117993	0,382470369	-0,593818531	0,552633493	0,763934535
hsa-miR-1224-5p	2,573446192	-0,428473885	0,747508538	-0,573202663	0,566507481	0,770748337
hsa-miR-187-3p	28,9676094	-0,21266985	0,369106074	-0,576174774	0,56458627	0,770748337
hsa-miR-202-3p	16,89405316	0,254127948	0,436322622	0,58243129	0,56027621	0,770748337
hsa-miR-3148	20,54418666	0,22224124	0,387275801	0,573857804	0,566064029	0,770748337
hsa-miR-373-5p	2,639661594	0,345428698	0,598727021	0,576938547	0,56398097	0,770748337
hsa-miR-508-5p	3,833238915	0,308905126	0,537882502	0,574298521	0,56576581	0,770748337
hsa-miR-3180-3p	4,820353321	0,332521397	0,59219248	0,561508982	0,574450609	0,779503845
hsa-miR-30c-2-3p	14,03391647	0,241479511	0,435790421	0,554118447	0,579497786	0,784294124
hsa-miR-501-3p	121,3990641	0,139633155	0,253041418	0,551819367	0,581072117	0,7843715
hsa-miR-1285-3p	4,788001184	-0,234869817	0,441319373	-0,532199199	0,594588037	0,787168162
hsa-miR-25-5p	6,579321991	0,287342271	0,540018113	0,53209747	0,59465849	0,787168162
hsa-miR-3133	1,778781791	0,532611646	0,978793063	0,544151432	0,586337271	0,787168162
hsa-miR-3663-3p	8,469244966	0,269099633	0,499967734	0,538233999	0,590415511	0,787168162
hsa-miR-522-3p	93,13208674	0,17136477	0,316692433	0,541107876	0,58843323	0,787168162
hsa-miR-7-1-3p	6,095221248	-0,216581144	0,406824496	-0,532369967	0,594469782	0,787168162
hsa-miR-873-3p	5,873045715	0,228306303	0,429845201	0,531136098	0,595324471	0,787168162
hsa-miR-93-3p	1,555358592	0,345093621	0,642662535	0,536974854	0,591284984	0,787168162
hsa-miR-30b-5p	228,1711605	-0,149486756	0,286726932	-0,521355826	0,602118918	0,790600325
hsa-miR-548ad-5p	1,348923643	-0,422421628	0,81110521	-0,520797577	0,602507791	0,790600325
hsa-miR-548ae-5p	1,348923643	-0,422421628	0,81110521	-0,520797577	0,602507791	0,790600325
hsa-miR-3617-5p	3,975295535	-0,287630548	0,563940033	-0,510037471	0,61002521	0,79843806
hsa-miR-375-3p	10877,90157	0,178778204	0,3590838	0,49787321	0,618573411	0,807581953
hsa-miR-3620-5p	19,84770929	-0,256889192	0,519000296	-0,494969259	0,62062182	0,808215317
hsa-miR-589-5p	16,77351234	0,169789834	0,35062948	0,484242893	0,628213499	0,816046178
hsa-miR-1246	172,8198435	-0,29318234	0,608701054	-0,481652427	0,630052876	0,816384304
hsa-miR-27a-5p	9,698519368	-0,243840693	0,513315214	-0,475031105	0,634764803	0,820433508
hsa-miR-212-3p	5,280331764	0,215969862	0,464362573	0,465088865	0,64186782	0,827545294
hsa-miR-629-5p	244,96578	-0,138124597	0,300653901	-0,459413955	0,645936929	0,828658542
hsa-miR-92b-3p	46,73894861	0,168760428	0,365627327	0,461564045	0,644393984	0,828658542
hsa-miR-1303	1,330661665	0,315755158	0,705712031	0,447427767	0,654566231	0,835976269
hsa-miR-488-3p	1,322782301	-0,365341623	0,821432361	-0,444761663	0,656492002	0,835976269
hsa-miR-552-3p	16,32062146	-0,216232341	0,484928126	-0,445905959	0,655665177	0,835976269
hsa-miR-512-3p	6,235221703	-0,211051117	0,479505442	-0,440143319	0,659833309	0,838166636
hsa-miR-379-5p	6,73048366	0,158906799	0,36528086	0,435026349	0,663543302	0,840813448
hsa-miR-548b-3p	1,639703254	-0,360326719	0,833257427	-0,432431453	0,665427862	0,841139865
hsa-miR-509-3-5p	263,5704225	0,150778112	0,35242488	0,427830499	0,66877453	0,843308371
hsa-miR-1292-5p	1,310819303	0,396554834	0,94407205	0,420047214	0,674450963	0,843981738
hsa-miR-1298-5p	1,23726082	-0,347120283	0,848220317	-0,409233635	0,682368213	0,843981738
hsa-miR-203a-3p	393,9603012	-0,192776545	0,460608636	-0,418525684	0,675562812	0,843981738
hsa-miR-31-5p	12,26141378	-0,185625781	0,444479388	-0,417625173	0,676221189	0,843981738
hsa-miR-493-3p	8,734653381	0,215806726	0,527339538	0,409236763	0,682365918	0,843981738
hsa-miR-519d-3p	9,330050666	-0,193655429	0,462878366	-0,418372176	0,675675026	0,843981738
hsa-miR-520e-3p	1,430440848	0,326942042	0,795431176	0,411024928	0,681054256	0,843981738
hsa-miR-6821-5p	9,849899297	0,218370258	0,528025279	0,413560234	0,679196201	0,843981738
hsa-miR-148a-5p	155,963908	-0,156384068	0,385164892	-0,406018491	0,684729014	0,84488043
hsa-let-7e-5p	97,89105274	0,12133205	0,303787505	0,399397762	0,689600143	0,846848631
hsa-miR-183-5p	325,7263759	0,110303964	0,275946001	0,399730249	0,68935521	0,846848631
hsa-let-7b-5p	5041,049644	-0,116641499	0,321128887	-0,363223314	0,716438073	0,849700095
hsa-miR-100-5p	765,4198335	-0,143579811	0,368052404	-0,390106979	0,696457442	0,849700095
hsa-miR-155-5p	149,3611961	0,137596176	0,360549936	0,381628624	0,70273685	0,849700095
hsa-miR-204-3p	187,8438263	0,200553104	0,525006466	0,382001208	0,702460469	0,849700095
hsa-miR-215-3p	4,624440996	-0,219721282	0,581588239	-0,377795264	0,705582693	0,849700095
hsa-miR-296-3p	6,388950823	-0,186037099	0,47770316	-0,389440796	0,696950099	0,849700095
hsa-miR-361-5p	109,3747695	0,123725387	0,342848233	0,360875091	0,718192825	0,849700095
hsa-miR-3663-5p	72,04888297	0,187676551	0,503004256	0,373111259	0,70906564	0,849700095
hsa-miR-369-3p	7,375660108	-0,166883637	0,434262288	-0,384292262	0,700761842	0,849700095
hsa-miR-369-5p	2,985342519	-0,239968661	0,646506259	-0,371177631	0,710505233	0,849700095
hsa-miR-378a-3p	205,5267248	-0,112610848	0,311230966	-0,361824049	0,717483519	0,849700095
hsa-miR-3911	1,585063704	0,36331949	0,964300246	0,376770089	0,706344471	0,849700095
hsa-miR-4423-5p	1,681749784	0,385076703	1,034165599	0,372354972	0,709628574	0,849700095
hsa-miR-548d-5p	2,468583674	-0,244151845	0,67389878	-0,362297503	0,717129724	0,849700095
hsa-miR-6724-5p	1,273323852	0,400558688	1,079697289	0,370991659	0,710643744	0,849700095
hsa-miR-708-5p	1,329401237	0,303406704	0,840832572	0,360840807	0,718218456	0,849700095
hsa-miR-145-3p	6,425130322	-0,242374384	0,677226791	-0,357892493	0,720423769	0,850363216
hsa-miR-624-5p	3,226932535	-0,255465201	0,725735419	-0,352008727	0,724831718	0,852874911
hsa-miR-889-3p	1,388525626	0,233075263	0,664695007	0,350649937	0,725850988	0,852874911
hsa-miR-330-5p	10,0265288	0,111680603	0,332669864	0,33571001	0,737089557	0,862161314
hsa-miR-520g-3p	29,17347052	0,141926048	0,421417235	0,336782733	0,736280691	0,862161314
hsa-miR-449a	61,39230111	-0,196482558	0,599222864	-0,327895628	0,742990579	0,867101872
hsa-miR-145-5p	42,43982728	-0,185335125	0,596650961	-0,310625704	0,756085185	0,878418068
hsa-miR-520a-3p	93,30924777	-0,093010062	0,29754252	-0,312594187	0,754588995	0,878418068
hsa-miR-130b-5p	2,183678313	0,251642117	0,841456482	0,299055415	0,764897764	0,879884995
hsa-miR-219a-2-3p	6,496186613	0,163696365	0,558688072	0,29300136	0,769521112	0,879884995
hsa-miR-324-5p	5,454643567	-0,154994654	0,521201898	-0,297379296	0,766176948	0,879884995
hsa-miR-345-5p	98,87487744	0,105009554	0,348057103	0,301702086	0,762879178	0,879884995
hsa-miR-4705	4,465817409	-0,189211092	0,649952186	-0,291115401	0,770963061	0,879884995
hsa-miR-486-3p	12,37320444	-0,138642474	0,4621138	-0,300018034	0,7641634	0,879884995
hsa-miR-548ay-5p	2,334244605	-0,208372795	0,708883236	-0,293945158	0,76879981	0,879884995
hsa-miR-6507-5p	3,603662931	0,155791514	0,524884123	0,296811253	0,766610614	0,879884995
hsa-let-7i-3p	3,04756745	-0,148836727	0,567074396	-0,262464198	0,792963584	0,901015764
hsa-miR-222-3p	198,6039993	-0,098205629	0,373567783	-0,262885702	0,792638677	0,901015764
hsa-miR-574-5p	130,573701	-0,109405625	0,423774706	-0,258169313	0,79627624	0,902795649
hsa-miR-365a-5p	1,476164338	0,202328797	0,815272423	0,248173237	0,804000372	0,909558408
hsa-miR-125b-1-3p	4,850730742	0,120299466	0,495516601	0,242775853	0,808179042	0,910301884
hsa-miR-143-3p	421,5951477	0,106870713	0,438002231	0,243995818	0,807234059	0,910301884
hsa-miR-1291	3,205975431	0,120946297	0,508648132	0,237779891	0,812051816	0,912675628
hsa-miR-184	100,0132886	-0,09676605	0,424373217	-0,228021105	0,819629834	0,913917346
hsa-miR-202-5p	134,815502	-0,118287257	0,528854401	-0,223666961	0,823016456	0,913917346
hsa-miR-373-3p	14,55665981	-0,080803802	0,358258249	-0,225546242	0,821554356	0,913917346
hsa-miR-3939	1,186692393	-0,183457474	0,795787782	-0,230535676	0,817675544	0,913917346
hsa-miR-892c-3p	19,31780888	0,096969348	0,435410882	0,22270768	0,823763023	0,913917346
hsa-miR-93-5p	117,7946077	-0,056954569	0,255205726	-0,223171204	0,823402263	0,913917346
hsa-miR-671-3p	6,424641373	-0,164784234	0,778438287	-0,211685674	0,832352265	0,92146921
hsa-miR-642a-3p	2,144858648	0,15091245	0,724824899	0,208205388	0,835068601	0,922500998
hsa-miR-1307-3p	52,9995787	0,070323238	0,356172194	0,197441685	0,843481911	0,929808418
hsa-miR-5698	3,193943674	0,10699777	0,557333508	0,191981584	0,847756631	0,932532294
hsa-miR-26a-2-3p	2,068161903	0,110589932	0,588524002	0,187910658	0,850946688	0,934054008
hsa-miR-221-3p	699,1764262	-0,062209853	0,340484359	-0,182709871	0,85502567	0,934771552
hsa-miR-505-3p	6,869205413	0,06643628	0,364100951	0,182466648	0,855216526	0,934771552
hsa-miR-509-5p	1,369412225	-0,149328042	0,841361036	-0,177483905	0,859128307	0,937066107
hsa-miR-892b	10,42893688	0,067950532	0,415597107	0,163500975	0,870124005	0,947061286
hsa-miR-455-5p	2,739560321	-0,091397135	0,57797725	-0,158132755	0,874352191	0,949664039
hsa-miR-126-5p	3,345519248	0,099924555	0,661283296	0,151107031	0,879891287	0,953676719
hsa-miR-378c	9,33732211	-0,054817641	0,378229485	-0,144932225	0,884764383	0,956952272
hsa-miR-190b-5p	34,52428383	0,06022316	0,426096354	0,141336953	0,887603759	0,957068519
hsa-miR-342-5p	23,79825672	-0,050735229	0,36211382	-0,140108514	0,888574254	0,957068519
hsa-miR-194-5p	455,7028327	-0,043883362	0,32314848	-0,135799377	0,891979884	0,958739293
hsa-miR-517-5p	7,870365734	0,067270286	0,509140542	0,13212518	0,89488528	0,959866576
hsa-miR-450b-5p	2,738484699	0,075407486	0,604256494	0,124793837	0,900686767	0,960711535
hsa-miR-451a	256,465174	-0,072726485	0,570630126	-0,127449431	0,898584695	0,960711535
hsa-miR-584-5p	4,650633257	-0,04789015	0,387873849	-0,123468366	0,901736223	0,960711535
hsa-miR-7162-3p	8,680746752	-0,089279159	0,733367453	-0,121738644	0,903106007	0,960711535
hsa-let-7a-5p	2949,232679	0,029142173	0,249914484	0,116608578	0,90717024	0,962126922
hsa-miR-1287-5p	6,91463073	0,041092133	0,356202764	0,115361634	0,908158488	0,962126922
hsa-miR-122-5p	8799,298533	-0,03385155	0,313499685	-0,107979533	0,914011927	0,962902406
hsa-miR-132-5p	224,6906332	0,032124887	0,299136376	0,10739211	0,914477913	0,962902406
hsa-miR-186-5p	718,2012498	-0,02221069	0,201597933	-0,110173205	0,912272012	0,962902406
hsa-miR-1908-5p	3,387641131	-0,078154607	0,750367231	-0,104155143	0,917046231	0,963644108
hsa-miR-3138	1,692877288	-0,086112284	0,849222832	-0,101401282	0,919231919	0,963981546
hsa-miR-16-5p	600,159667	-0,033065414	0,360632971	-0,091687163	0,926946597	0,964220164
hsa-miR-200c-3p	1239,390551	0,029360483	0,303480961	0,096745716	0,922928334	0,964220164
hsa-miR-548bc	1,34255743	0,070787732	0,753110372	0,093993835	0,925114054	0,964220164
hsa-miR-891a-5p	538,7173878	0,031690076	0,354577846	0,089374099	0,928784607	0,964220164
hsa-miR-99b-3p	20,17532288	-0,031868407	0,333299327	-0,095614976	0,923826371	0,964220164
hsa-miR-500a-3p	18,10866783	-0,025739358	0,301748561	-0,085300681	0,93202235	0,965642395
hsa-miR-655-3p	1,992716591	-0,049548956	0,60905894	-0,081353303	0,935160985	0,966956459
hsa-miR-363-5p	13,46757934	0,048285073	0,623873585	0,077395604	0,938308838	0,968274789
hsa-let-7f-1-3p	1,312715714	0,044972867	0,689073156	0,065265737	0,947962422	0,968570301
hsa-miR-122b-5p	3,900410094	-0,038485675	0,556162209	-0,069198652	0,944831496	0,968570301
hsa-miR-214-5p	1,91271388	0,075404088	1,011053068	0,074579753	0,940549084	0,968570301
hsa-miR-520g-5p	1,381258999	0,057307961	0,852955237	0,067187537	0,946432407	0,968570301
hsa-miR-628-5p	2,715611067	0,041613197	0,617516406	0,067388002	0,94627282	0,968570301
hsa-miR-641	1,210848055	0,043792111	0,717403791	0,061042486	0,951325373	0,970089187
hsa-miR-203b-3p	2,677070477	-0,036241632	0,633552037	-0,057203876	0,95438279	0,971291147
hsa-miR-181c-3p	1,323144501	0,036552866	0,70139924	0,052114208	0,958437692	0,973501546

Supplementary Table 3. Differentially expressed tsRNAs in high vs. low-TADS sperm samples. Misregulated tsRNAs (log2 FC < -1.0 or > 1.0 , Padj < 0.05) are indicated in red.

Id	baseMean	log2FoldChange	lfcSE	stat	pvalue	padj
mature-mt_tRNA-Gln-TTG_5_end	5730,980626	3,088135873	0,674811003	4,576297452	4,73E-06	0,000312293
mature-tRNA-Asp-GTC_CCA_end	31,97973334	-1,851476879	0,405537774	-4,565485625	4,98E-06	0,000312293
mature-tRNA-Glu-TTC_CCA_end	153,2206504	-1,166777219	0,251061027	-4,647384872	3,36E-06	0,000312293
mature-tRNA-Asp-GTC	4611,683078	-1,22510616	0,289851266	-4,226671756	2,37E-05	0,001114714
mature-tRNA-SeC-TCA	4500,595701	-1,346442003	0,324298611	-4,151858678	3,30E-05	0,001239995
mature-mt_tRNA-Thr-TGT_3_end	74,41654988	-2,831747942	0,696578882	-4,065222212	4,80E-05	0,001503584
mature-mt_tRNA-Leu-TAA_5_end	37,63722835	2,25596565	0,585612026	3,852321245	0,000117003	0,003142377
mature-tRNA-Met-CAT	286,7334901	-1,301079204	0,347529922	-3,743790458	0,000181265	0,004259724
mature-tRNA-Gly-CCC_5_end	135838,8617	-1,396229467	0,381483495	-3,659999675	0,000252216	0,005268503
mature-tRNA-Gly-GCC_5_end	192054,7197	-1,190960559	0,334839836	-3,556806661	0,00037539	0,007032663
mature-tRNA-Lys-TTT_5_end	4283,314934	-1,436314289	0,406587769	-3,532605748	0,000411486	0,007032663
mature-tRNA-Phe-GAA_5_end	19,19093783	-1,723596698	0,500057611	-3,446796251	0,000567276	0,008887328
mature-tRNA-Glu-CTC_CCA_end	9,440462548	-0,947845188	0,283725531	-3,340711657	0,00083564	0,012084634
mature-mt_tRNA-Gln-TTG_CCA_end	9,181184924	2,192544989	0,703532892	3,1164783	0,001830252	0,020240428
mature-tRNA-Arg-CCG_3_end	39,30557729	-1,506196235	0,483280326	-3,116609873	0,001829435	0,020240428
mature-tRNA-Asp-GTC_3_end	244,7359519	-1,144386279	0,367082136	-3,117521028	0,001823789	0,020240428
mature-tRNA-Lys-TTT	597,0359037	-0,941990904	0,297377488	-3,167660442	0,001536709	0,020240428
mature-tRNA-Gly-CCC	4970,449938	-0,7490759	0,247148252	-3,030876786	0,002438447	0,024527403
mature-tRNA-Leu-TAA_5_end	173,0325411	1,165379701	0,385507895	3,022972334	0,002503051	0,024527403
mature-tRNA-Lys-CTT	7035,395619	-1,071320679	0,357640834	-2,995521141	0,002739763	0,024527403
mature-tRNA-Val-AAC	922,4398744	-1,080345331	0,360062364	-3,000439473	0,002695903	0,024527403
mature-tRNA-Leu-CAG_5_end	780,6281463	0,959393791	0,322621289	2,973746068	0,002941884	0,025139738
mature-tRNA-Val-AAC_5_end	9057,898866	-0,613704265	0,208027596	-2,950109867	0,003176609	0,02596533
mature-tRNA-Arg-TCT	106,1387627	-0,653913133	0,223582775	-2,924702639	0,003447855	0,027008199
mature-tRNA-Arg-CCT_3_end	120,8472534	-1,268980423	0,441056688	-2,877136785	0,004013016	0,029630768
mature-tRNA-Glu-TTC_3_end	134,46027	-0,715014763	0,249088259	-2,87052776	0,004097872	0,029630768
mature-tRNA-Gly-GCC	10207,20078	-0,686979166	0,247568106	-2,774909806	0,005521702	0,038447406
mature-mt_tRNA-Lys-TTT_CCA_end	37,44495639	2,403302598	0,885954809	2,71266951	0,006674364	0,043268288
mature-tRNA-Val-CAC_5_end	9631,77677	-0,623271072	0,229641838	-2,714100693	0,006645596	0,043268288
mature-mt_tRNA-Trp-TCA_CCA_end	36,05998381	1,850092496	0,689446742	2,683445122	0,007286793	0,045663905
mature-tRNA-Leu-CAA_5_end	226,2097124	0,743370063	0,284067664	2,616876745	0,008873838	0,053815532
mature-tRNA-Lys-CTT_5_end	3720,500539	-1,032662901	0,399804764	-2,582917951	0,009796862	0,057556563
mature-mt_tRNA-Phe-GAA_5_end	50,37515013	2,082753059	0,813754901	2,559435349	0,010484235	0,057971655
mature-tRNA-Val-TAC	359,2428044	-0,842278015	0,328122784	-2,566959854	0,010259449	0,057971655
mature-tRNA-Tyr-GTA_CCA_end	93,1962749	-0,606398806	0,238662911	-2,540817101	0,011059376	0,059404646
mature-mt_tRNA-Ala-TGC	281,4366073	1,268109318	0,532607468	2,380945433	0,01726827	0,085432492
mature-mt_tRNA-Glu-TTC	4133,443597	1,353450584	0,567936206	2,383103188	0,017167381	0,085432492
mature-tRNA-Thr-CGT	54,10505299	-0,633413406	0,26405412	-2,398801454	0,016448831	0,085432492
mature-tRNA-Val-CAC	1155,253243	-0,742737649	0,313743299	-2,367341872	0,017916374	0,086366112
mature-mt_tRNA-Ser-TGA	1716,647795	1,704493346	0,726686943	2,345567596	0,018998133	0,089291227
mature-tRNA-Leu-CAG_CCA_end	38,67101329	1,035731055	0,452402231	2,28940307	0,022055944	0,098726606
mature-tRNA-Ser-GCT_5_end	3862,586792	0,709060112	0,309263484	2,292737906	0,021863099	0,098726606
mature-mt_tRNA-Ala-TGC_5_end	235,0373447	1,751476878	0,772959989	2,265934723	0,02345538	0,100218443
mature-tRNA-Leu-TAA	603,9368425	-0,519175886	0,228482018	-2,272283356	0,023069401	0,100218443
mature-tRNA-Tyr-GTA	331,7607979	-0,77295598	0,345025913	-2,240283847	0,025072501	0,104747338
mature-tRNA-Ala-TGC	34,78637604	0,581785234	0,263547391	2,207516573	0,02727799	0,111483961
mature-mt_tRNA-Ser-TGA_5_end	1642,052075	1,723622012	0,795552637	2,16657193	0,03026751	0,121070039
mature-tRNA-His-GTG	8704,695324	-0,848089797	0,39850886	-2,128157948	0,03332399	0,130518961
mature-tRNA-Trp-CCA_3_end	24,7515656	-0,616680054	0,293265361	-2,102805638	0,035482763	0,136137948
mature-mt_tRNA-Glu-TTC_3_end	52,31481454	1,107371744	0,538699199	2,055640228	0,039817207	0,147302967
mature-mt_tRNA-Pro-TGG_CCA_end	11,09342431	1,511219605	0,735686052	2,054163731	0,039959847	0,147302967
mature-mt_tRNA-Lys-TTT	59,54713833	1,142664622	0,561480809	2,035091146	0,041841703	0,151273848
mature-tRNA-Ile-TAT	85,65175314	-0,607743652	0,301309745	-2,017006294	0,043694857	0,154993076
mature-tRNA-Ser-CGA	56,33462376	-0,495546126	0,253758351	-1,95282687	0,050840114	0,173780752
mature-tRNA-iMet-CAT_CCA_end	66,71454922	-0,67234215	0,34366314	-1,956398786	0,050418195	0,173780752
mature-tRNA-Gly-CCC_CCA_end	71,67427766	0,719137618	0,383187963	1,876722885	0,060556084	0,201331437
mature-tRNA-Tyr-GTA_5_end	67,94014703	-0,700510418	0,373966306	-1,873191264	0,061041978	0,201331437
mature-tRNA-Asn-GTT	352,8628203	-0,418111345	0,225828121	-1,85145828	0,064103651	0,207784249
mature-tRNA-Gln-TTG	362,5314197	-0,473631515	0,260696548	-1,816792431	0,069248902	0,216979894
mature-tRNA-Leu-CAG	1413,861287	-0,622482174	0,342019375	-1,820020207	0,068755928	0,216979894
mature-tRNA-Gly-CCC_3_end	17,01206422	0,619503019	0,342667734	1,807882556	0,070624781	0,21766326
mature-tRNA-Ala-CGC	63,26444272	0,391478615	0,217625032	1,798867584	0,072039629	0,218442747
mature-tRNA-Ile-TAT_CCA_end	8,230160918	-0,708353584	0,397157745	-1,783557272	0,074495634	0,218830924
mature-tRNA-SeC-TCA_5_end	483,7467003	0,649753088	0,363026573	1,789822388	0,073482469	0,218830924
mature-mt_tRNA-His-GTG_CCA_end	91,84729313	1,152327451	0,655904055	1,756853676	0,078942753	0,225260088
mature-tRNA-Ala-TGC_5_end	1241,989932	0,503391399	0,287805119	1,749070342	0,080278861	0,225260088
mature-tRNA-Gly-GCC_CCA_end	58,30340511	0,640868967	0,365103632	1,755307018	0,079206807	0,225260088
mature-tRNA-Ile-AAT	204,195376	-0,353300032	0,207371189	-1,70370838	0,088435582	0,244498373
mature-tRNA-Gln-CTG_CCA_end	124,0987507	0,50791772	0,30274248	1,677722001	0,093401382	0,254484926
mature-tRNA-Cys-GCA_5_end	13975,71564	-0,61061199	0,377400498	-1,617941665	0,10567517	0,283813314
mature-mt_tRNA-Val-TAC_CCA_end	115,9682852	1,060958977	0,66490358	1,595658392	0,110565083	0,290898044
mature-tRNA-Val-CAC_CCA_end	88,215755	-0,432111865	0,271444546	-1,591897393	0,111407761	0,290898044
mature-mt_tRNA-Trp-TCA	37,23915721	0,974124994	0,615832333	1,581802289	0,113694712	0,292802821
mature-tRNA-Glu-TTC	7323,68055	-0,398346942	0,255434283	-1,559488954	0,118880697	0,29799428
mature-tRNA-Lys-CTT_3_end	215,7856364	-0,639421337	0,408379496	-1,565752796	0,117406501	0,29799428
mature-mt_tRNA-Thr-TGT_CCA_end	376,8492385	1,032432417	0,66523868	1,551972922	0,120668703	0,298496265
mature-tRNA-Trp-CCA	488,225614	-0,301076131	0,198236207	-1,518774674	0,128819223	0,314519661
mature-tRNA-Val-AAC_CCA_end	80,61462498	-0,417567834	0,276621809	-1,509526077	0,131164396	0,316139827
mature-mt_tRNA-Asn-GTT	193,6782547	0,86886799	0,605958683	1,433873321	0,151608416	0,336651119
mature-mt_tRNA-Met-CAT	59,48771667	0,72919043	0,498354831	1,463195266	0,143413956	0,336651119
mature-tRNA-Arg-ACG_3_end	32,2495199	0,523090932	0,365345311	1,431771303	0,152209283	0,336651119
mature-tRNA-Arg-CCG	43404,99752	-0,616469205	0,429868484	-1,434087931	0,151547171	0,336651119
mature-tRNA-Arg-TCT_3_end	9,701191583	-0,715326974	0,49599265	-1,442212852	0,149242336	0,336651119
mature-tRNA-Gln-TTG_CCA_end	82,01975582	0,427186882	0,293239293	1,456785951	0,145175475	0,336651119
mature-tRNA-Thr-AGT	37,22576973	-0,389813788	0,265832837	-1,466386893	0,142542915	0,336651119
mature-tRNA-Asp-GTC_5_end	131,190337	0,487014945	0,346821886	1,404222065	0,160252733	0,350319929
mature-tRNA-Leu-CAA	16687,87082	-0,608364884	0,439233203	-1,385061239	0,166033743	0,354708451
mature-tRNA-Leu-TAG	22011,30619	-0,615021059	0,442515546	-1,389829271	0,164580727	0,354708451
mature-tRNA-SeC-TCA_3_end	20,42749375	-0,880587373	0,647292062	-1,360417382	0,173697881	0,366912378
mature-tRNA-Gln-CTG	914,5564585	-0,346914581	0,258977894	-1,33955287	0,180390755	0,376816244
mature-mt_tRNA-Gln-TTG	179,2717188	0,633997012	0,477152549	1,328709263	0,183943908	0,380015985
mature-tRNA-Leu-AAG	18153,60312	-0,598154795	0,476214461	-1,256061802	0,209093564	0,427278152
mature-mt_tRNA-Val-TAC	157,3622433	0,69173471	0,557981069	1,239709999	0,215082678	0,434790789
mature-tRNA-Gly-TCC_CCA_end	27,30870463	0,370344239	0,302518861	1,224202146	0,22087598	0,44175196
mature-mt_tRNA-Ile-GAT	13,84659359	-0,760594857	0,627917231	-1,211297954	0,225781233	0,446809176
mature-tRNA-Val-TAC_5_end	366,3067504	-0,424093109	0,369852578	-1,146654464	0,251524457	0,492568729
mature-mt_tRNA-Ser-GCT	113,8162813	0,666879251	0,591854703	1,126761766	0,25984322	0,503613663
mature-tRNA-Cys-GCA_CCA_end	9,316781209	0,542081451	0,49463362	1,095925203	0,273111509	0,5239282
mature-mt_tRNA-Leu-TAG	21,75848699	0,715791149	0,670792141	1,067083386	0,285934183	0,537556265
mature-mt_tRNA-Thr-TGT	266,6984938	-0,633182676	0,592422646	-1,068802282	0,285158771	0,537556265
mature-tRNA-Arg-TCG	291,0116424	-0,38295542	0,363053685	-1,054817608	0,29150875	0,542146257
mature-tRNA-Ile-GAT_5_end	119,0176038	-0,440201411	0,41960873	-1,049075911	0,294143182	0,542146257
mature-tRNA-Thr-TGT_5_end	27,01302345	0,394323034	0,378264405	1,042453452	0,297201496	0,542464866
mature-tRNA-Thr-TGT_3_end	19,53496945	0,403855526	0,393436928	1,026480987	0,304664921	0,550740434
mature-mt_tRNA-Leu-TAA	18,48691544	0,561641031	0,562177393	0,99904592	0,317772447	0,55792556
mature-tRNA-Ala-AGC_5_end	2943,369673	0,249912311	0,251570282	0,993409512	0,320510428	0,55792556
mature-tRNA-Asn-GTT_3_end	142,5665687	-0,425080426	0,425412024	-0,999220524	0,317687876	0,55792556
mature-tRNA-Leu-CAA_CCA_end	37,35572379	0,519213006	0,514517279	1,009126471	0,312913984	0,55792556
mature-mt_tRNA-Lys-TTT_5_end	126,5725068	0,687706355	0,70477483	0,975781662	0,329172674	0,567747364
mature-mt_tRNA-Tyr-GTA	31,74636988	0,529255489	0,553981998	0,95536586	0,339392712	0,580052999
mature-tRNA-Trp-CCA_CCA_end	71,65823961	0,281796805	0,296875483	0,949208744	0,342514455	0,580114573
mature-tRNA-Arg-CCT	13391,01844	-0,393148027	0,431577672	-0,910955437	0,362318854	0,603158181
mature-tRNA-Ser-CGA_5_end	33,67492048	0,324917266	0,356839275	0,910542334	0,362536567	0,603158181
mature-tRNA-Ala-CGC_5_end	1788,687312	0,250666125	0,302119524	0,829691912	0,406712996	0,670719677
mature-tRNA-Gln-CTG_3_end	102,2513224	-0,264172533	0,325696716	-0,811099776	0,417308375	0,682208473
mature-mt_tRNA-Met-CAT_5_end	35,86431225	0,466926073	0,596405771	0,782899991	0,433685843	0,696114
mature-mt_tRNA-Met-CAT_CCA_end	12,00514863	0,502892075	0,658538987	0,763648145	0,44507695	0,696114
mature-mt_tRNA-Val-TAC_5_end	13,85041795	-0,436918626	0,554645793	-0,787743514	0,430846747	0,696114
mature-tRNA-Arg-ACG_CCA_end	46,77426626	0,323327125	0,429366223	0,753033441	0,451429819	0,696114
mature-tRNA-Gly-TCC	1500,332251	-0,166404668	0,221127522	-0,752528074	0,451733553	0,696114
mature-tRNA-Leu-AAG_5_end	10477,46735	0,230238017	0,300014209	0,767423708	0,442829637	0,696114
mature-tRNA-Leu-TAG_5_end	11359,77494	0,22022508	0,29080292	0,757300097	0,448870093	0,696114
mature-mt_tRNA-Ser-GCT_5_end	172,0699628	-0,52228316	0,719769401	-0,725625678	0,468068275	0,715421428
mature-tRNA-Ser-CGA_CCA_end	18,17575844	0,287414738	0,406427161	0,707174042	0,479458328	0,72692069
mature-mt_tRNA-Glu-TTC_5_end	27,01232229	-0,452483606	0,655255693	-0,690545098	0,48985146	0,727403929
mature-tRNA-Pro-AGG_3_end	8,819651168	0,316648089	0,464309308	0,681976613	0,495253739	0,727403929
mature-tRNA-Pro-CGG_3_end	8,83645247	0,322375099	0,462165427	0,697531836	0,485470023	0,727403929
mature-tRNA-Pro-TGG_3_end	8,819651168	0,316648089	0,464309308	0,681976613	0,495253739	0,727403929
mature-tRNA-His-GTG_5_end	9163,976585	-0,227178772	0,349663865	-0,649706175	0,515882034	0,751828081
mature-mt_tRNA-Glu-TTC_CCA_end	310,3456151	-0,362487335	0,567552153	-0,638685509	0,523027542	0,753833199
mature-tRNA-Thr-AGT_CCA_end	12,42971635	0,28888888	0,454777264	0,635231582	0,525277388	0,753833199
mature-mt_tRNA-Phe-GAA	321,1898134	-0,325547027	0,519082803	-0,627158182	0,530555552	0,755639725
mature-tRNA-Glu-CTC	19157,45098	-0,225963822	0,36658928	-0,616395062	0,537633812	0,759963583
mature-tRNA-Asn-GTT_CCA_end	8,43298156	0,232540931	0,389389282	0,59719397	0,550377885	0,772171958
mature-tRNA-Pro-CGG	3868,657364	-0,268864106	0,45670049	-0,588709914	0,556055886	0,774359308
mature-tRNA-Pro-AGG	3555,798352	-0,265086203	0,457877334	-0,578945896	0,56262568	0,777747264
mature-tRNA-Ser-AGA_CCA_end	28,44910779	0,189950708	0,337642732	0,562578991	0,573721601	0,787296796
mature-tRNA-Ala-CGC_CCA_end	9,712922457	-0,267909794	0,511060936	-0,524222799	0,600123585	0,787863279
mature-tRNA-Gln-TTG_5_end	51,49924952	0,237053285	0,461717954	0,513415783	0,607660508	0,787863279
mature-tRNA-Glu-TTC_5_end	8929,477295	-0,175264691	0,334280139	-0,52430483	0,600066538	0,787863279
mature-tRNA-Lys-TTT_CCA_end	32,10777502	0,199358439	0,384534675	0,518440734	0,6041508	0,787863279
mature-tRNA-Pro-AGG_5_end	297,6720842	0,268779386	0,512635585	0,524308874	0,600063726	0,787863279
mature-tRNA-Pro-CGG_5_end	306,3774067	0,274844825	0,507788232	0,541258752	0,588329247	0,787863279
mature-tRNA-Tyr-GTA_3_end	9,346359921	-0,278777019	0,523555326	-0,532469074	0,594401156	0,787863279
mature-tRNA-iMet-CAT	8819,685626	-0,132982132	0,242640465	-0,548062469	0,58364901	0,787863279
pre-tRNA-Leu-CAA	17,17456282	-0,225823654	0,449283646	-0,50263048	0,615224096	0,79220637
mature-tRNA-Gln-CTG_5_end	61,07522028	0,305032616	0,621714142	0,490631619	0,623687019	0,797640541
mature-tRNA-Val-TAC_CCA_end	65,99672169	0,117513882	0,247270672	0,475243913	0,634613131	0,800719924
pre-tRNA-Tyr-GTA	61,42934405	0,254356737	0,531703133	0,478381113	0,632378974	0,800719924
mature-tRNA-Ser-GCT_CCA_end	10,93401712	-0,266099009	0,572674165	-0,464660405	0,642174667	0,804858916
mature-tRNA-Ala-TGC_CCA_end	12,4547157	-0,20230989	0,502810829	-0,402357862	0,687420677	0,836262537
mature-tRNA-Arg-ACG	175,5298968	0,113321922	0,276701482	0,409545771	0,682139184	0,836262537
mature-tRNA-Cys-GCA	1360,356653	-0,1377159	0,35185101	-0,39140402	0,695498621	0,836262537
mature-tRNA-Ile-AAT_CCA_end	72,57269235	-0,113618808	0,297804538	-0,381521411	0,702816387	0,836262537
mature-tRNA-Phe-GAA_CCA_end	26,14855811	0,128561592	0,325452196	0,395024502	0,692824806	0,836262537
mature-tRNA-Pro-TGG_5_end	365,9841161	0,186602463	0,485875313	0,384054217	0,700938263	0,836262537
mature-tRNA-Ser-GCT	164,6878358	-0,112411889	0,275856595	-0,407501182	0,683639926	0,836262537
mature-tRNA-Ser-TGA_CCA_end	64,42199236	0,1287463	0,316098777	0,407297685	0,683789363	0,836262537
mature-tRNA-Ser-TGA	82,09769308	0,08794202	0,241749254	0,363773698	0,716027005	0,846623126
mature-tRNA-Gln-TTG_3_end	25,4249363	-0,121924596	0,346734533	-0,351636727	0,725110718	0,852005093
mature-tRNA-Ser-AGA	42,31793894	0,088742957	0,264619629	0,335360445	0,737353202	0,861008708
mature-mt_tRNA-Pro-TGG	38,85216816	0,143524125	0,492549935	0,291389999	0,770753063	0,888966723
mature-tRNA-Lys-CTT_CCA_end	35,7704528	-0,114099291	0,389217558	-0,293150421	0,769407178	0,888966723
mature-tRNA-Ala-AGC_CCA_end	11,45383858	0,123934324	0,505112292	0,245359945	0,80617774	0,907553384
mature-tRNA-Glu-CTC_5_end	71936,39156	0,096560219	0,39287085	0,245781072	0,80585171	0,907553384
mature-tRNA-Lys-TTT_3_end	59,41837372	0,096375609	0,390475773	0,246815848	0,805050745	0,907553384
mature-tRNA-SeC-TCA_CCA_end	11,5802633	0,135655712	0,548804798	0,247183902	0,804765903	0,907553384
mature-tRNA-Ile-AAT_5_end	42,19685938	0,061368069	0,319413811	0,192127162	0,847642599	0,948552432
mature-tRNA-Leu-TAG_CCA_end	25,31039093	-0,050438043	0,278977448	-0,18079613	0,856527601	0,951012992
mature-tRNA-Pro-TGG	6247,700144	-0,079823377	0,452444426	-0,17642692	0,859958557	0,951012992
mature-tRNA-Ile-GAT	155,5058925	-0,063411146	0,402937389	-0,157372207	0,874951516	0,961935
mature-tRNA-Val-CAC_3_end	9,588723045	-0,05540331	0,403046955	-0,13746118	0,890666275	0,973518952
mature-mt_tRNA-His-GTG	646,1922071	0,041131522	0,542804496	0,075775941	0,939597357	0,980726607
mature-mt_tRNA-Phe-GAA_CCA_end	11,08933643	0,052056528	0,743885401	0,069979231	0,94421019	0,980726607
mature-tRNA-Gly-TCC_5_end	2022,329339	-0,025340915	0,261507123	-0,096903347	0,922803151	0,980726607
mature-tRNA-Pro-AGG_CCA_end	29,79955968	-0,031520194	0,3565242	-0,08840969	0,92955106	0,980726607
mature-tRNA-Pro-CGG_CCA_end	29,79955968	-0,031520194	0,3565242	-0,08840969	0,92955106	0,980726607
mature-tRNA-Pro-TGG_CCA_end	29,79955968	-0,031520194	0,3565242	-0,08840969	0,92955106	0,980726607
mature-tRNA-Ser-AGA_5_end	2381,327137	-0,029854837	0,32034677	-0,093195374	0,92574835	0,980726607
mature-tRNA-Ser-TGA_5_end	2419,103826	-0,023633607	0,31563466	-0,074876462	0,940313004	0,980726607
mature-tRNA-iMet-CAT_5_end	69,92234413	0,035910323	0,370307903	0,096974229	0,92274686	0,980726607
mature-mt_tRNA-Arg-TCG	15,7858179	-0,044604995	0,72583073	-0,061453715	0,950997875	0,982349453
mature-mt_tRNA-Cys-GCA	8,60140402	-0,019950974	0,473059459	-0,042174346	0,966359713	0,988806979
mature-tRNA-Phe-GAA	73,28718694	-0,008537265	0,250343519	-0,034102202	0,972795653	0,988806979
mature-tRNA-Thr-TGT_CCA_end	49,31902762	0,016504077	0,488132508	0,033810649	0,973028144	0,988806979
mature-tRNA-Ala-AGC	189,8186528	-0,004445716	0,236275362	-0,018815827	0,984988028	0,995579297
mature-tRNA-Leu-AAG_CCA_end	21,5686005	-0,002115208	0,278343779	-0,007599264	0,993936723	0,999251893
mature-tRNA-Thr-TGT	238,1070876	-0,000149987	0,211491391	-0,000709187	0,999434151	0,999434151
mature-mt_tRNA-Ala-TGC_3_end	0,479530036	1,447828624	1,523981101	0,950030563	0,342096723	NA
mature-mt_tRNA-Ala-TGC_CCA_end	1,164957443	1,708573243	0,902127712	1,893937211	0,058233345	NA
mature-mt_tRNA-Arg-TCG_5_end	0,165039852	-0,789791135	2,985753928	-0,264519835	0,791379383	NA
mature-mt_tRNA-Arg-TCG_CCA_end	1,76415234	0,219334916	0,911777632	0,240557465	0,80989812	NA
mature-mt_tRNA-Asn-GTT_5_end	2,322758574	0,341060475	1,0285127	0,331605507	0,740187161	NA
mature-mt_tRNA-Asn-GTT_CCA_end	2,822063253	1,001672693	0,877235901	1,141851002	0,253515958	NA
mature-mt_tRNA-Asp-GTC	2,094568998	0,032560628	1,035473249	0,031445166	0,974914522	NA
mature-mt_tRNA-Asp-GTC_CCA_end	0,338508523	0,213780534	2,272350632	0,094079026	0,925046381	NA
mature-mt_tRNA-Cys-GCA_3_end	2,884431848	0,464545142	0,864077057	0,537620039	0,590839393	NA
mature-mt_tRNA-Cys-GCA_5_end	1,12485142	2,051924857	0,999326324	2,053308123	0,040042704	NA
mature-mt_tRNA-Cys-GCA_CCA_end	2,912513263	-0,047635285	0,7877116	-0,060473002	0,951778918	NA
mature-mt_tRNA-Gln-TTG_3_end	0,863082689	0,912460788	1,33191331	0,685075208	0,493296464	NA
mature-mt_tRNA-Gly-TCC	2,979747901	-0,049943914	0,70327119	-0,071016579	0,943384561	NA
mature-mt_tRNA-Gly-TCC_5_end	0,513496321	0,657711632	1,77544782	0,370448303	0,711048489	NA
mature-mt_tRNA-His-GTG_3_end	2,674266201	-1,503874145	0,796123875	-1,888995158	0,058892478	NA
mature-mt_tRNA-Ile-GAT_CCA_end	2,414099751	2,107579997	1,118209984	1,884780165	0,059459524	NA
mature-mt_tRNA-Leu-TAA_CCA_end	4,595207024	1,439538617	1,257557153	1,144710293	0,252329186	NA
mature-mt_tRNA-Leu-TAG_5_end	0,359968692	-1,181330685	1,954557264	-0,604398094	0,545579003	NA
mature-mt_tRNA-Leu-TAG_CCA_end	0,470809559	0,692774373	1,712390685	0,404565605	0,685796846	NA
mature-mt_tRNA-Lys-TTT_3_end	0,763929082	-0,497796967	1,076652697	-0,462356123	0,643825959	NA
mature-mt_tRNA-Met-CAT_3_end	3,97950396	-1,220732101	0,955081576	-1,278144329	0,201198541	NA
mature-mt_tRNA-Phe-GAA_3_end	0,579362279	-1,712600331	1,364373987	-1,255227927	0,20939603	NA
mature-mt_tRNA-Pro-TGG_3_end	1,556102098	0,423494265	0,814734205	0,519794385	0,603206894	NA
mature-mt_tRNA-Pro-TGG_5_end	0,402082414	0,491849493	1,771284939	0,277679487	0,781258409	NA
mature-mt_tRNA-Ser-GCT_CCA_end	1,111033142	1,077733072	1,277138777	0,84386528	0,398744692	NA
mature-mt_tRNA-Ser-TGA_CCA_end	0,547424595	1,353904685	1,688753976	0,801718133	0,422716024	NA
mature-mt_tRNA-Thr-TGT_5_end	3,325874378	0,794859685	0,826408518	0,961824167	0,336137936	NA
mature-mt_tRNA-Trp-TCA_3_end	1,427403782	-1,039766026	1,034217813	-1,005364647	0,314721297	NA
mature-mt_tRNA-Trp-TCA_5_end	2,851378891	0,826142438	1,041039673	0,793574404	0,427443238	NA
mature-mt_tRNA-Tyr-GTA_5_end	0,689353921	1,293998027	1,31577547	0,983448967	0,32538652	NA
mature-mt_tRNA-Val-TAC_3_end	6,632073449	-0,310447924	0,624545659	-0,497078027	0,619134029	NA
mature-tRNA-Ala-AGC_3_end	1,843287802	1,081985878	0,888377973	1,217934158	0,223248995	NA
mature-tRNA-Ala-CGC_3_end	2,51357371	0,23776025	0,789142253	0,301289468	0,763193771	NA
mature-tRNA-Ala-TGC_3_end	3,150041391	-0,123717516	0,775510412	-0,159530439	0,873250978	NA
mature-tRNA-Arg-ACG_5_end	1,969854547	1,12023321	0,913121335	1,226817475	0,219891201	NA
mature-tRNA-Arg-CCG_5_end	3,251885458	-0,793218813	1,009154472	-0,786023187	0,431853903	NA
mature-tRNA-Arg-CCG_CCA_end	7,136193143	-0,259369321	0,393407397	-0,659289384	0,509709958	NA
mature-tRNA-Arg-CCT_CCA_end	5,748044128	-0,950726492	0,446610998	-2,128757456	0,033274333	NA
mature-tRNA-Arg-TCG_3_end	4,51881164	0,942284809	0,570728286	1,651021743	0,098734135	NA
mature-tRNA-Arg-TCG_5_end	0,655245654	-0,863206878	1,208602294	-0,714219129	0,47509169	NA
mature-tRNA-Arg-TCG_CCA_end	7,678506887	-0,163789935	0,427312074	-0,383302847	0,701495228	NA
mature-tRNA-Arg-TCT_5_end	7,930228692	-0,009734669	0,427834662	-0,022753344	0,981847024	NA
mature-tRNA-Arg-TCT_CCA_end	3,396460604	0,470046518	0,442064168	1,063299294	0,287646245	NA
mature-tRNA-Asn-GTT_5_end	3,534192842	-0,413081347	0,541485964	-0,762866213	0,445543188	NA
mature-tRNA-Cys-GCA_3_end	3,859423431	1,371446166	0,559375625	2,451744597	0,014216553	NA
mature-tRNA-Glu-CTC_3_end	1,154161897	-1,377370771	0,826183704	-1,667148316	0,095484917	NA
mature-tRNA-Gly-GCC_3_end	2,241428962	0,497534217	0,660943721	0,752763361	0,451592128	NA
mature-tRNA-Gly-TCC_3_end	2,53529016	0,4424083	0,589598885	0,750354708	0,453041101	NA
mature-tRNA-His-GTG_3_end	1,122861935	1,463255653	1,356007982	1,079090737	0,28054728	NA
mature-tRNA-His-GTG_CCA_end	5,857396831	0,08323533	0,437671503	0,190177633	0,849169938	NA
mature-tRNA-Ile-AAT_3_end	5,585307369	0,911523335	0,521991767	1,746240825	0,080769114	NA
mature-tRNA-Ile-GAT_CCA_end	3,58611794	0,481771743	0,573879485	0,839499852	0,401188872	NA
mature-tRNA-Ile-TAT_3_end	1,108121459	-0,054746829	1,109005551	-0,049365694	0,960627867	NA
mature-tRNA-Leu-AAG_3_end	1,352041888	-0,227728825	0,730216094	-0,311864976	0,755143141	NA
mature-tRNA-Leu-CAA_3_end	3,671028725	0,491984294	0,534502405	0,920452911	0,357336129	NA
mature-tRNA-Leu-CAG_3_end	6,209732816	1,064196027	0,586839015	1,813437755	0,069764331	NA
mature-tRNA-Leu-TAA_3_end	0,92395035	1,26461826	0,864797987	1,46232794	0,143651365	NA
mature-tRNA-Leu-TAA_CCA_end	1,742160144	0,490267256	0,778712874	0,629586684	0,528965038	NA
mature-tRNA-Leu-TAG_3_end	1,472358216	0,017028417	0,75273487	0,022622064	0,981951744	NA
mature-tRNA-Met-CAT_3_end	4,265746973	-1,367502948	0,690976652	-1,979087058	0,047806205	NA
mature-tRNA-Met-CAT_5_end	6,975584239	-0,786913889	0,461655629	-1,704547371	0,088278874	NA
mature-tRNA-Met-CAT_CCA_end	2,970421494	-0,239587156	0,630164077	-0,380198054	0,703798404	NA
mature-tRNA-Phe-GAA_3_end	6,394731148	0,914407804	0,425656636	2,148228704	0,031695592	NA
mature-tRNA-Ser-AGA_3_end	2,908816077	0,987679427	0,517374799	1,909021139	0,056259365	NA
mature-tRNA-Ser-CGA_3_end	7,117488065	0,601215802	0,443406528	1,355902011	0,175130348	NA
mature-tRNA-Ser-GCT_3_end	3,325190074	0,579227837	0,530075807	1,092726416	0,274513938	NA
mature-tRNA-Ser-TGA_3_end	6,50439549	0,282566587	0,422685132	0,668503728	0,503812103	NA
mature-tRNA-Thr-AGT_3_end	4,449069104	-0,31473161	0,594304356	-0,529579847	0,59640327	NA
mature-tRNA-Thr-CGT_3_end	2,387822639	0,275527511	0,612816505	0,449608503	0,652992756	NA
mature-tRNA-Thr-CGT_5_end	0,456590543	0,633265155	1,551419469	0,408184355	0,683138333	NA
mature-tRNA-Thr-CGT_CCA_end	4,030089499	-0,211039385	0,557886685	-0,378283602	0,705219926	NA
mature-tRNA-Trp-CCA_5_end	2,131339517	2,001493557	0,92761889	2,157667958	0,030953657	NA
mature-tRNA-Tyr-ATA	0,37918255	-1,189527531	1,714830456	-0,693670635	0,487888786	NA
mature-tRNA-Val-AAC_3_end	8,057128366	-1,91E-05	0,415558223	-4,60E-05	0,999963302	NA
mature-tRNA-Val-TAC_3_end	6,988377487	0,085130218	0,554553502	0,15351128	0,877995095	NA
mature-tRNA-iMet-CAT_3_end	5,099234216	-0,1112323	0,456127939	-0,243862064	0,807337651	NA
pre-tRNA-Arg-TCT	3,524089093	0,138122548	0,58552499	0,235895223	0,813513975	NA
pre-tRNA-Ile-TAT	3,15145032	0,341992569	0,676502895	0,50553009	0,61318657	NA

Supplementary Table 4. Differentially expressed piRNA clusters in high vs. low-TADS sperm samples. Misregulated piRNA clusters (log2 FC < -1.0 or > 1.0 , Padj < 0.05) are indicated in red.

ID	baseMean	log2FoldChange	lfcSE	stat	pvalue	padj
ID=1896	6,263704877	-4,028461386	0,694953666	-5,796733773	6,76E-09	3,15E-05
ID=792	8,257139279	-1,304914545	0,316315464	-4,12535805	3,70E-05	0,045379705
ID=1763	2338,850156	2,437354777	0,592496189	4,11370541	3,89E-05	0,045379705
ID=4759	7,197205284	-2,260230535	0,54097167	-4,178094084	2,94E-05	0,045379705
ID=2230	3,293746048	-2,395793929	0,633869283	-3,779634055	0,000157059	0,146441828
ID=4999	42,73115372	2,647211011	0,731363813	3,619554269	0,000295111	0,229301183
ID=5638	0,913021727	-2,829984079	0,797431601	-3,548873753	0,000386883	0,257663758
ID=3250	5,639369544	2,29158721	0,654100847	3,503415752	0,000459332	0,267675681
ID=2755	4,136217309	-1,527572215	0,456328261	-3,347529276	0,000815354	0,421581237
ID=5591	1,040906791	2,659027132	0,807720157	3,292015322	0,000994722	0,421581237
ID=5899	1,718029566	-2,305157948	0,696336306	-3,310408962	0,000931598	0,421581237
ID=238	5,242665179	-1,216351969	0,43442365	-2,799921158	0,005111509	0,649846851
ID=772	2,458381143	1,300247068	0,463779694	2,803587745	0,005053748	0,649846851
ID=824	9,298736106	-1,989232345	0,70828918	-2,808503081	0,004977241	0,649846851
ID=933	244,3464516	2,247003429	0,734750124	3,058187207	0,002226804	0,649846851
ID=957	1,631052495	1,981866191	0,655998056	3,021146438	0,002518195	0,649846851
ID=1164	773,1463645	1,065111418	0,364542534	2,921775425	0,003480424	0,649846851
ID=1167	87,7571897	-1,387974069	0,460283454	-3,015476781	0,002565756	0,649846851
ID=1210	12,18494798	1,610943685	0,57600153	2,796769803	0,005161629	0,649846851
ID=1404	9,611799439	1,354365936	0,458918115	2,951214809	0,003165267	0,649846851
ID=1561	173,6983809	1,525703931	0,542033643	2,814777183	0,004881106	0,649846851
ID=1718	88,28303062	-0,892289938	0,317519863	-2,810186203	0,004951285	0,649846851
ID=1723	90,48619487	-0,882001426	0,307704549	-2,866390597	0,004151816	0,649846851
ID=1728	92,19640879	-0,898359153	0,311108986	-2,887602712	0,003881898	0,649846851
ID=1736	91,79190296	-0,885860456	0,314665445	-2,815245429	0,004874	0,649846851
ID=2100	29,9586154	1,771021065	0,614820148	2,880551442	0,003969802	0,649846851
ID=2262	5,470889668	-1,707476198	0,608296188	-2,806981587	0,00500081	0,649846851
ID=2315	1,481662633	1,856026952	0,661398774	2,806214683	0,005012728	0,649846851
ID=2322	29,59262444	1,74892477	0,627214411	2,78840017	0,005296907	0,649846851
ID=2468	2,971661028	-1,699802262	0,561554475	-3,026958802	0,002470276	0,649846851
ID=2823	2,95351386	1,4054133	0,486824605	2,886898657	0,003890595	0,649846851
ID=3187	5,287277078	1,462785502	0,489128429	2,990595957	0,002784336	0,649846851
ID=3297	7,448681194	1,354864541	0,470591466	2,879067387	0,003988531	0,649846851
ID=4180	10,2158626	1,459765035	0,484951065	3,010128527	0,002611372	0,649846851
ID=5003	11,25351013	0,949202306	0,311233482	3,049807819	0,002289878	0,649846851
ID=5126	470,678474	1,254703371	0,424479113	2,955865985	0,003117925	0,649846851
ID=5758	2,484278674	-2,032028188	0,718775188	-2,827070581	0,004697597	0,649846851
ID=6183	2,687636195	1,782795144	0,583820456	3,053670223	0,002260604	0,649846851
ID=165	3,809642011	1,398074586	0,509274843	2,745226092	0,006046922	0,654223921
ID=495	9,463382618	-1,241122562	0,463454744	-2,677980054	0,007406763	0,654223921
ID=728	3,295565035	-1,337706573	0,487040184	-2,746604113	0,006021577	0,654223921
ID=736	49,9160775	1,346751251	0,504102299	2,671583236	0,007549435	0,654223921
ID=1159	112,1443384	-1,251688729	0,462863102	-2,704230956	0,006846269	0,654223921
ID=1724	87,93927014	-0,876222838	0,320424812	-2,73456613	0,006246255	0,654223921
ID=1735	90,74128535	-0,811863985	0,302109641	-2,687315708	0,007202884	0,654223921
ID=2688	1,708805142	1,87289247	0,694836107	2,695444941	0,007029469	0,654223921
ID=2838	63,8038474	1,419884153	0,529295342	2,682593331	0,007305376	0,654223921
ID=2999	3,781983206	1,654061614	0,619424333	2,670320694	0,007577883	0,654223921
ID=3272	1,491948433	2,076103193	0,747756583	2,776442548	0,005495735	0,654223921
ID=3393	2,599143611	-1,995565862	0,736105456	-2,710978224	0,006708503	0,654223921
ID=4285	195,0992263	-0,846067432	0,314562193	-2,689666626	0,007152343	0,654223921
ID=4287	1229,332127	-0,98219972	0,365155788	-2,689810083	0,007149269	0,654223921
ID=4804	2,005301872	-1,959346526	0,724601892	-2,704031755	0,006850374	0,654223921
ID=5973	25,22925746	-1,073640926	0,400714365	-2,67931729	0,007377245	0,654223921
ID=2526	68,52664605	2,060999303	0,778950476	2,645866929	0,008148186	0,654945597
ID=3431	2283,473806	1,743356468	0,658162773	2,648822663	0,00807727	0,654945597
ID=3664	0,77690176	-2,134066198	0,804982108	-2,651072833	0,008023654	0,654945597
ID=4344	0,922499042	-2,668941873	1,005230398	-2,655054881	0,007929551	0,654945597
ID=3640	15,58433496	1,115017853	0,423427535	2,633314464	0,008455602	0,668135868
ID=1614	1,662927277	1,578653668	0,604556225	2,611260297	0,00902092	0,678315006
ID=3008	27,66962668	-1,080892374	0,413860765	-2,611729513	0,00900855	0,678315006
ID=4304	278,461638	-0,841846172	0,321079547	-2,621924	0,008743493	0,678315006
ID=1654	1,381932019	-1,737138677	0,669168668	-2,595965351	0,009432558	0,687102873
ID=4778	7,365554184	-2,219657025	0,854513902	-2,597566897	0,009388683	0,687102873
ID=3240	3,861727447	-1,172129661	0,458377035	-2,557129987	0,010553977	0,753157273
ID=5207	44,97203541	-1,234510115	0,483444629	-2,553570856	0,010662458	0,753157273
ID=1024	28,59088288	1,191001543	0,469774641	2,53526146	0,011236343	0,781812328
ID=1843	2,07249544	-1,55231355	0,613542326	-2,530083883	0,011403526	0,781812328
ID=2305	1,631622197	1,754943678	0,696806295	2,518553134	0,01178381	0,796175701
ID=4907	4,11857659	1,148139062	0,457251181	2,510959206	0,012040361	0,796283396
ID=4925	8,694036385	-1,096537009	0,437141328	-2,508426772	0,01212701	0,796283396
ID=1204	2,731251487	1,47759759	0,599312827	2,465486341	0,013682739	0,811818771
ID=1920	3,094550112	-1,249129351	0,504946735	-2,473784391	0,013369035	0,811818771
ID=2076	4,777381152	1,44295197	0,585094669	2,466185466	0,013656061	0,811818771
ID=3017	0,780522387	-2,761892203	1,116740164	-2,47317352	0,01339191	0,811818771
ID=3490	5,077213086	1,264342113	0,515095262	2,454579191	0,014104959	0,811818771
ID=3671	2,281267021	1,16147919	0,472698656	2,457123955	0,014005435	0,811818771
ID=4028	581,9781962	1,719664336	0,699644959	2,457909994	0,013974819	0,811818771
ID=4309	301,6014497	-0,828827469	0,334483595	-2,4779316	0,013214648	0,811818771
ID=5447	111,5723519	-0,698765254	0,284209002	-2,458631675	0,013946762	0,811818771
ID=5953	2,302353058	-1,422587865	0,577091034	-2,465101314	0,013697452	0,811818771
ID=5022	496,6563462	1,716950048	0,700847309	2,44982042	0,014292748	0,812595004
ID=3486	3,041152159	1,174225885	0,480227397	2,445145553	0,014479369	0,813286986
ID=388	3,847905219	1,375199772	0,565788075	2,430591652	0,015074193	0,824058925
ID=1215	380,9997209	1,395267922	0,574169143	2,430064277	0,015096145	0,824058925
ID=1362	1,100715134	-1,787992616	0,740344795	-2,415080957	0,015731713	0,824058925
ID=1947	1,387083788	-2,013803036	0,832673645	-2,418478173	0,015585581	0,824058925
ID=2375	14,95381185	-1,056647128	0,436047702	-2,423237462	0,015382868	0,824058925
ID=3388	4,936788691	-1,377538261	0,569726599	-2,417893536	0,015610643	0,824058925
ID=3270	4,164912963	-1,077545519	0,447152277	-2,409795444	0,015961467	0,826803985
ID=6	1,242909632	-1,568896538	0,663838971	-2,363369139	0,018109621	0,841601952
ID=203	1,72190835	1,948395365	0,813468006	2,395171478	0,016612594	0,841601952
ID=1271	1,283381925	1,626572566	0,696054029	2,336848143	0,019447082	0,841601952
ID=1754	808,1837112	1,570486266	0,662934238	2,368992543	0,017836613	0,841601952
ID=2069	362,2256357	0,980995305	0,412734992	2,376816419	0,017462777	0,841601952
ID=2221	0,861755903	1,760059969	0,760570298	2,314131873	0,02066049	0,841601952
ID=2473	14,75022249	-0,580147454	0,249971899	-2,32085069	0,020294904	0,841601952
ID=2520	126,5466464	1,763762184	0,753055058	2,342142405	0,019173398	0,841601952
ID=2561	12,38534981	-1,043387432	0,450478993	-2,316173337	0,020548806	0,841601952
ID=2566	1,260448447	1,777050398	0,768376829	2,312732933	0,020737327	0,841601952
ID=3021	3,998302091	1,526974058	0,647150245	2,359535624	0,018297824	0,841601952
ID=3023	1,793919832	-1,508855934	0,646745033	-2,332999645	0,019648164	0,841601952
ID=3340	79,61177188	-0,777486171	0,330685015	-2,3511382	0,018716081	0,841601952
ID=3437	1,430608717	1,682470469	0,724051728	2,323688218	0,02014221	0,841601952
ID=3864	1,305772188	1,641698266	0,708730714	2,316392155	0,020536867	0,841601952
ID=4619	2,695106476	1,227857947	0,531007688	2,312316702	0,020760237	0,841601952
ID=4621	1,222603889	2,042959783	0,868549225	2,352151983	0,018665147	0,841601952
ID=4659	8,214806027	1,025888118	0,430674052	2,382052303	0,017216452	0,841601952
ID=4698	3,369312909	-1,166408165	0,491045665	-2,375355794	0,017532042	0,841601952
ID=4824	40,30680644	-1,283967683	0,555159904	-2,312788934	0,020734246	0,841601952
ID=4972	2,42212207	1,490411987	0,640441233	2,327164321	0,019956521	0,841601952
ID=4980	1,64644364	1,441963597	0,610697211	2,361175997	0,018217083	0,841601952
ID=4994	59,5016524	1,13805269	0,486762599	2,338003559	0,019387064	0,841601952
ID=5491	2,024093618	1,950857186	0,822898808	2,370713346	0,017753794	0,841601952
ID=6173	3,382892152	1,312435404	0,565772871	2,319721345	0,020355956	0,841601952
ID=5325	2,316954516	-1,484725305	0,645245586	-2,301023575	0,021390299	0,859668724
ID=174	1,263763972	-2,026676411	0,887013745	-2,284830898	0,022322745	0,864281743
ID=2707	1,583441004	-2,010373914	0,877764408	-2,290334279	0,022001946	0,864281743
ID=2825	77,75963702	1,675358474	0,733094336	2,285324538	0,022293805	0,864281743
ID=4370	27,57041642	0,65753451	0,287546533	2,286706445	0,022212963	0,864281743
ID=4977	0,94024079	1,52349777	0,66733093	2,282971912	0,022432023	0,864281743
ID=4240	225,8088891	-0,729190382	0,320653558	-2,274075445	0,022961451	0,86915724
ID=6097	4,058599802	0,90913705	0,399666543	2,274738947	0,022921595	0,86915724
ID=6168	71,13281805	1,548416162	0,681676874	2,27148114	0,023117867	0,86915724
ID=2894	2,136518102	1,199332891	0,53254976	2,252057893	0,024318614	0,899788709
ID=4965	8,904613419	0,732784564	0,32532145	2,252493848	0,024291081	0,899788709
ID=4019	27,33102273	0,646512094	0,287729563	2,24694358	0,024643633	0,904634782
ID=4280	1,176049959	-2,178892019	0,972753203	-2,239922739	0,025095939	0,911926275
ID=4769	2,265227342	-1,765887051	0,789114113	-2,237809491	0,025233481	0,911926275
ID=3453	2,215829885	-1,689221604	0,757627724	-2,229619575	0,025772709	0,917193665
ID=3734	3,660224929	1,375817927	0,616995895	2,229865609	0,025756366	0,917193665
ID=73	7,964542823	-0,639637042	0,293204263	-2,181540731	0,029143445	0,928695961
ID=117	6,805320586	-0,929949328	0,4296464	-2,164452739	0,03042962	0,928695961
ID=173	2,263976778	1,123626591	0,52298526	2,148486154	0,031675155	0,928695961
ID=511	0,545864191	-2,001582268	0,930893406	-2,15017343	0,031541499	0,928695961
ID=618	3,215217184	-1,073761585	0,487653865	-2,201892906	0,027672874	0,928695961
ID=994	14,56462362	-0,890604396	0,413783371	-2,152344584	0,031370225	0,928695961
ID=1009	4,795363038	-1,008079018	0,454698596	-2,217026897	0,026621254	0,928695961
ID=1016	4,178661158	1,447337755	0,669178529	2,162857433	0,030552147	0,928695961
ID=1284	0,756609065	-2,4208936	1,116008594	-2,169242794	0,030064256	0,928695961
ID=1314	1,732769548	-1,836752235	0,843268883	-2,178133536	0,029396096	0,928695961
ID=1695	9,889900642	1,202033362	0,558342952	2,152858486	0,031329802	0,928695961
ID=1832	2,175103161	-1,607060748	0,748560862	-2,146867182	0,031803857	0,928695961
ID=2397	22,07954598	0,56815521	0,261389655	2,173594855	0,029735575	0,928695961
ID=2728	1,125387905	-1,749286032	0,8069184	-2,167859886	0,030169349	0,928695961
ID=2778	1,105503207	1,567060957	0,711393665	2,202804205	0,027608552	0,928695961
ID=3205	8,228058092	-0,847673937	0,388161651	-2,183816807	0,028975712	0,928695961
ID=3900	2,966098445	-0,896108911	0,417210349	-2,147858781	0,031724976	0,928695961
ID=3929	2,581253975	1,055341006	0,482261943	2,188314923	0,028646671	0,928695961
ID=4020	1,328129121	1,659240929	0,759142096	2,185678988	0,028839099	0,928695961
ID=4021	0,888994827	-1,76198203	0,813827346	-2,165056309	0,030383373	0,928695961
ID=4046	2,745863384	-1,370201513	0,638490541	-2,146001272	0,031872877	0,928695961
ID=4542	0,809611624	-1,585700085	0,732916211	-2,163548931	0,030498985	0,928695961
ID=4837	0,500530004	-2,554360022	1,171191866	-2,180991941	0,029184013	0,928695961
ID=4912	1,487148055	1,331799527	0,606047402	2,197517095	0,027983537	0,928695961
ID=4935	5,072689725	0,962845351	0,444608612	2,165602115	0,030341605	0,928695961
ID=5035	0,832613983	1,730559134	0,784318469	2,206449552	0,027352539	0,928695961
ID=5535	3,547145801	1,293116756	0,5820378	2,2217058	0,026303194	0,928695961
ID=5718	2,014720101	-1,381441647	0,643383674	-2,147150608	0,031781293	0,928695961
ID=6092	4,610211562	-0,818776908	0,37470284	-2,185136646	0,028878829	0,928695961
ID=4547	1,307763989	-2,272087158	1,060645802	-2,142173337	0,032179536	0,931807441
ID=2818	1,71917494	-1,892151179	0,88478468	-2,138544237	0,032472598	0,934489202
ID=1188	1,243568931	1,417875991	0,664538554	2,133624879	0,0328735	0,940222429
ID=1978	0,833491363	-1,891127397	0,887797219	-2,1301344	0,033160519	0,942648412
ID=2668	9,113552161	1,780406993	0,837491357	2,125881036	0,033513166	0,943300455
ID=5541	1,229998645	1,359032058	0,64013137	2,123051802	0,033749511	0,943300455
ID=5909	0,739072541	1,841183008	0,867433773	2,122563205	0,033790471	0,943300455
ID=1342	0,714834988	-1,881074366	0,888334217	-2,117530013	0,034214891	0,949463222
ID=5292	20,53526364	-0,7346596	0,347967968	-2,111285139	0,034747811	0,958546112
ID=6233	23,70204838	-0,652964278	0,309646179	-2,108743212	0,034966753	0,958911772
ID=5127	3,276345643	-0,929561969	0,442506811	-2,100672678	0,035669709	0,972468917
ID=561	0,613600964	-1,961697882	0,93950981	-2,088001488	0,0367977	0,995972588
ID=4044	0,643746325	-2,088603903	1,001144738	-2,086215733	0,036959086	0,995972588
ID=2	17,91450177	0,13020064	0,232059417	0,56106596	0,574752573	0,999930046
ID=3	18,68699985	0,013194049	0,229724286	0,057434278	0,954199258	0,999930046
ID=4	368,7404703	0,852523892	0,662669778	1,286498826	0,198269028	0,999930046
ID=5	35,66038048	-0,032271105	0,225596252	-0,143048055	0,886252229	0,999930046
ID=7	1,099651811	-0,527306304	0,822208152	-0,641329452	0,521308658	0,999930046
ID=8	13,09848631	-0,184078924	0,379830955	-0,484633812	0,627936125	0,999930046
ID=9	10,57664106	-0,16199831	0,274538065	-0,590075952	0,55513973	0,999930046
ID=10	2,462771082	-0,163861169	0,639644446	-0,256175395	0,797815407	0,999930046
ID=11	47,12156306	0,090446343	0,327449701	0,276214462	0,78238335	0,999930046
ID=13	30,76504426	0,088572919	0,375240943	0,23604279	0,813399466	0,999930046
ID=14	8,04358848	0,511664257	0,377523689	1,355316957	0,175316595	0,999930046
ID=15	4,083783255	0,176842552	0,588716341	0,300386688	0,763882216	0,999930046
ID=16	1,431798485	-1,433341424	0,71681768	-1,999589945	0,045544561	0,999930046
ID=17	3,120128373	0,215994907	0,615645493	0,350842992	0,725706142	0,999930046
ID=18	8,673616827	-0,462464782	0,376997863	-1,226703987	0,219933869	0,999930046
ID=19	0,812846996	0,504491512	0,876894106	0,575316345	0,565077369	0,999930046
ID=20	7,580961305	-0,335778272	0,441745207	-0,760117522	0,44718434	0,999930046
ID=21	13,57552199	-0,130934933	0,292653745	-0,44740563	0,654582212	0,999930046
ID=22	40,47255721	-0,078678169	0,377582467	-0,208373471	0,834937368	0,999930046
ID=23	0,583111682	1,081513298	1,047953573	1,032024056	0,302060848	0,999930046
ID=24	1,369027487	0,692855513	0,702375898	0,986445456	0,323914568	0,999930046
ID=25	5,392000594	-0,073629691	0,432839912	-0,170108369	0,864924912	0,999930046
ID=26	0,428576347	0,34198816	1,209811635	0,282678848	0,777423032	0,999930046
ID=27	6,320042171	-0,32114833	0,426390521	-0,753178869	0,451342436	0,999930046
ID=29	4,53410604	0,3664272	0,426019766	0,860117838	0,389724089	0,999930046
ID=30	0,94215822	-0,91874861	0,787710531	-1,166353087	0,243471718	0,999930046
ID=31	0,31660896	-0,889553223	1,252557621	-0,710189461	0,477586655	0,999930046
ID=32	1,698060006	-1,154576254	0,598461224	-1,929241541	0,053700883	0,999930046
ID=33	2,909518007	-0,480033537	0,653951314	-0,734050879	0,46291773	0,999930046
ID=34	0,779551516	0,281788681	0,836167732	0,337000186	0,73611676	0,999930046
ID=35	23,535481	0,089508136	0,282805802	0,316500352	0,751622758	0,999930046
ID=36	1,643234385	-1,303847964	0,700616	-1,861002268	0,062743856	0,999930046
ID=37	2,935551501	-0,566302658	0,547062474	-1,035169994	0,300589529	0,999930046
ID=38	2,361557689	-0,61178823	0,635470339	-0,962732943	0,33568156	0,999930046
ID=39	0,723029182	0,015122546	0,917719968	0,016478389	0,986852743	0,999930046
ID=40	2,876854535	-0,274018548	0,60202175	-0,455163868	0,648991353	0,999930046
ID=41	1,427738128	-0,131356053	0,61446436	-0,213773266	0,830723873	0,999930046
ID=42	0,356435815	1,346846114	1,174398313	1,146839278	0,251448052	0,999930046
ID=44	1,939336492	-0,505762852	0,609636311	-0,829614055	0,406757028	0,999930046
ID=46	0,711977313	0,232491345	0,772917263	0,300797196	0,763569147	0,999930046
ID=48	9,057911731	0,001531677	0,484213273	0,003163228	0,997476114	0,999930046
ID=50	0,615354042	0,392501039	1,819387042	0,215732568	0,829196213	0,999930046
ID=51	0,379242129	-1,288185502	1,16856486	-1,102365428	0,270302835	0,999930046
ID=52	0,343310452	0,721386897	1,40618589	0,513009626	0,607944587	0,999930046
ID=53	6,859522771	-0,280992156	0,379926289	-0,739596506	0,459544862	0,999930046
ID=54	8,408038913	0,56945919	0,403769442	1,410357324	0,1584342	0,999930046
ID=57	0,418189651	0,339076557	1,120697855	0,302558406	0,762226417	0,999930046
ID=58	5,882793567	-0,766643832	0,561420772	-1,365542336	0,172082656	0,999930046
ID=61	0,82252127	-0,440992151	0,855433262	-0,51551906	0,606190354	0,999930046
ID=63	3,160352195	0,885735469	0,475435863	1,862996753	0,062462723	0,999930046
ID=64	0,953646199	-0,480489556	0,773621437	-0,621091316	0,534539543	0,999930046
ID=65	0,394117405	0,136087342	1,310443623	0,1038483	0,917289736	0,999930046
ID=66	1,160067098	0,572554152	0,721466597	0,79359759	0,427429736	0,999930046
ID=67	3,701756291	0,023241529	0,459938474	0,050531821	0,959698592	0,999930046
ID=68	6,825697497	-0,417573883	0,424867627	-0,982832901	0,325689686	0,999930046
ID=69	20,62572358	-0,337626186	0,284941207	-1,184897718	0,23605789	0,999930046
ID=71	151,0209354	0,112954307	0,266650019	0,423605095	0,671853839	0,999930046
ID=72	19,77392671	-0,289527966	0,326291169	-0,887330071	0,37490122	0,999930046
ID=75	161,5600668	0,110414267	0,255047663	0,432916208	0,665075645	0,999930046
ID=76	183,1090067	0,227925478	0,254700587	0,89487614	0,370853303	0,999930046
ID=78	20,68866018	-0,101383357	0,380286132	-0,266597566	0,78977903	0,999930046
ID=79	180,9213112	0,205772257	0,25587776	0,804181875	0,421291945	0,999930046
ID=80	4,270486963	0,46066635	0,417931066	1,102254384	0,270351094	0,999930046
ID=81	0,564811628	-0,856041008	1,003856271	-0,852752563	0,393796535	0,999930046
ID=82	1,553228024	0,376766027	0,641951643	0,586907177	0,557266049	0,999930046
ID=83	1,948142987	-0,418747514	0,574264091	-0,729189793	0,465885574	0,999930046
ID=84	1,450753233	0,146004833	0,641228009	0,227695658	0,819882848	0,999930046
ID=85	0,763459635	0,815906164	0,939513722	0,868434537	0,385156495	0,999930046
ID=86	0,854649231	0,430742635	0,82390958	0,522803285	0,601111153	0,999930046
ID=87	1,851215711	0,367241217	0,563723622	0,651456143	0,514752079	0,999930046
ID=88	0,653419155	1,210057007	0,977048547	1,238481968	0,215537404	0,999930046
ID=89	13,72153443	0,100261483	0,276495154	0,362615695	0,716891984	0,999930046
ID=90	2,898250687	0,777521366	0,587685913	1,323021956	0,185828076	0,999930046
ID=91	3,736261329	0,09346287	0,518797371	0,180152937	0,857032505	0,999930046
ID=92	0,50389249	1,38745442	1,252879158	1,107412803	0,268115493	0,999930046
ID=93	6,832650584	-0,39479557	0,391179882	-1,009243032	0,312858094	0,999930046
ID=94	0,641995437	-0,955107826	0,915928854	-1,042775126	0,297052454	0,999930046
ID=95	0,556650682	-1,233377782	1,220505993	-1,010546273	0,312233642	0,999930046
ID=96	2,182168517	-0,275381927	0,505178704	-0,545117845	0,58567247	0,999930046
ID=97	1,48416526	0,692822408	0,658014438	1,05289849	0,292387518	0,999930046
ID=98	44,48192035	0,200320602	0,327361629	0,611924503	0,540587709	0,999930046
ID=99	0,813689133	-1,887201531	1,934359207	-0,975621035	0,329252297	0,999930046
ID=100	2,81298865	-0,376783358	0,540922155	-0,696557451	0,486079792	0,999930046
ID=101	1,397729646	0,839168433	0,632056933	1,327678552	0,184284322	0,999930046
ID=102	62,5073283	0,29529413	0,532057238	0,555004441	0,578891623	0,999930046
ID=103	47,63455325	0,726804129	0,42637116	1,704627794	0,088263864	0,999930046
ID=104	0,864381444	0,662079734	0,92731348	0,713976178	0,47524191	0,999930046
ID=105	0,365453748	1,001338733	1,283719265	0,780029373	0,435373586	0,999930046
ID=106	0,779881036	-0,818604451	1,02068982	-0,802010988	0,422546602	0,999930046
ID=108	0,904188687	0,390211853	1,041560153	0,374641687	0,707926966	0,999930046
ID=110	12,91868913	-0,523836302	0,324413549	-1,614717709	0,106371841	0,999930046
ID=111	16,83260261	-0,705622812	0,383305081	-1,840890837	0,065637558	0,999930046
ID=112	16,67726937	-0,138854568	0,26002852	-0,533997457	0,593343294	0,999930046
ID=113	44,88015808	-0,108561536	0,261699754	-0,414832397	0,678264602	0,999930046
ID=114	0,893304891	0,511310581	0,832141082	0,614451795	0,538916818	0,999930046
ID=115	1,994741719	0,269861192	0,583003694	0,462880759	0,64344984	0,999930046
ID=116	33,1471594	-0,389382725	0,30338036	-1,283480333	0,199323845	0,999930046
ID=119	1,838231466	0,315653788	0,636992161	0,495537948	0,620220442	0,999930046
ID=120	2,942132377	-0,890299882	0,451975303	-1,969797634	0,048861568	0,999930046
ID=121	2,288868809	0,093294812	0,596948851	0,156286107	0,875807507	0,999930046
ID=122	2,180990562	-0,098119288	0,591609393	-0,165851472	0,868273838	0,999930046
ID=123	0,618440135	-1,397423286	0,908871781	-1,537536224	0,124162051	0,999930046
ID=124	1,951934998	0,486747515	0,551090954	0,883243522	0,377104712	0,999930046
ID=125	0,333551584	-0,095958528	1,063850993	-0,090199219	0,928128905	0,999930046
ID=126	22,95339656	-0,192869352	0,270259582	-0,713644823	0,475446833	0,999930046
ID=127	0,477298306	1,046287274	1,109036804	0,943419795	0,345466222	0,999930046
ID=128	0,568283767	-1,323121401	0,892390023	-1,482671665	0,138161666	0,999930046
ID=129	2,381246304	0,151336861	0,483109146	0,313256046	0,754086141	0,999930046
ID=130	11,08725184	0,095178609	0,326894274	0,291160221	0,770928784	0,999930046
ID=131	8,45490908	-0,190596563	0,313439447	-0,60808097	0,543133773	0,999930046
ID=133	2,04368819	-0,995098402	0,576908677	-1,724880282	0,08454905	0,999930046
ID=134	0,450463685	-0,723490052	1,270635895	-0,569392109	0,56909007	0,999930046
ID=135	2,118923167	0,86017833	0,520133468	1,653764625	0,098175346	0,999930046
ID=136	31,58724815	0,141361755	0,322430446	0,438425578	0,661077808	0,999930046
ID=137	5,696028673	0,466483686	0,364275347	1,280579896	0,200341265	0,999930046
ID=138	9,700853365	-0,060897371	0,421231025	-0,144570004	0,885050381	0,999930046
ID=139	1,03547688	-0,710815669	0,757313883	-0,938601133	0,347935571	0,999930046
ID=140	1,578427069	0,19444409	0,679636622	0,286100077	0,774801473	0,999930046
ID=141	9,28690693	0,081240237	0,358678949	0,22649848	0,820813742	0,999930046
ID=143	34,72096093	0,087552681	0,258165775	0,33913357	0,73450911	0,999930046
ID=144	6,86550258	-0,016638039	0,417452675	-0,039856109	0,968207843	0,999930046
ID=145	6,504134431	-0,270036183	0,474086487	-0,56959266	0,568954008	0,999930046
ID=146	9,649609903	-0,813367872	0,429333586	-1,89448927	0,058160097	0,999930046
ID=147	9,84382126	-0,415926295	0,313452195	-1,326920983	0,184534822	0,999930046
ID=148	4,778056615	0,333182022	0,354143679	0,940810304	0,34680208	0,999930046
ID=149	12,21977562	0,122189703	0,357559195	0,341732796	0,732551989	0,999930046
ID=150	1,015281912	-1,566234417	0,961622954	-1,628740673	0,103367929	0,999930046
ID=151	175,1238455	-0,467265221	0,346762678	-1,347507244	0,177816922	0,999930046
ID=152	1,730610608	0,801496124	0,546478358	1,466656662	0,142469478	0,999930046
ID=153	2,911113652	0,017357968	0,548051123	0,031672169	0,97473349	0,999930046
ID=154	9,904262717	-0,022658291	0,419253213	-0,054044407	0,956899784	0,999930046
ID=155	1,697208613	-0,914588158	0,640596426	-1,427713489	0,153374342	0,999930046
ID=156	1,260969661	1,385682787	0,738525023	1,87628414	0,060616274	0,999930046
ID=157	3,960258207	0,570430714	0,387218388	1,473149857	0,140710632	0,999930046
ID=158	1,822743303	1,209299821	0,633497566	1,908925758	0,05627167	0,999930046
ID=159	1,068318811	0,553145784	0,76152882	0,726362246	0,467616729	0,999930046
ID=161	1,789145606	-0,657192649	0,584005342	-1,125319584	0,260453634	0,999930046
ID=162	0,282548595	0,186654717	1,222371928	0,152698792	0,878635813	0,999930046
ID=163	7,377094575	-0,722520357	0,423471269	-1,706185073	0,087973627	0,999930046
ID=166	18,55722318	0,033372458	0,309522246	0,107819254	0,914139069	0,999930046
ID=167	47,93296654	-0,341920134	0,511872903	-0,667978577	0,504147267	0,999930046
ID=168	1,352621902	-0,04017018	0,629897402	-0,063772576	0,949151315	0,999930046
ID=169	1,313008983	-0,844619351	0,728862251	-1,158818897	0,246530013	0,999930046
ID=170	1,831610115	-0,342808048	0,679164683	-0,504749521	0,613734775	0,999930046
ID=171	7,333992677	-0,258584913	0,472675281	-0,547066715	0,584332899	0,999930046
ID=172	4,767345571	0,173597798	0,421721777	0,411640582	0,680602882	0,999930046
ID=175	0,288948943	0,33771235	1,59071361	0,212302421	0,831871107	0,999930046
ID=176	0,671147336	0,760755694	0,992441897	0,766549353	0,443349498	0,999930046
ID=180	1,165220978	-0,612067891	0,851753459	-0,718597482	0,472388965	0,999930046
ID=181	2,737382947	-0,039830082	0,53393742	-0,07459691	0,940535433	0,999930046
ID=182	0,562774287	0,498542521	0,891560063	0,559179961	0,576038908	0,999930046
ID=183	0,834584657	0,119057478	0,926874391	0,128450499	0,897792469	0,999930046
ID=184	0,753934984	0,270432447	0,806445813	0,335338646	0,737369644	0,999930046
ID=185	0,386420946	-0,498631958	1,170921077	-0,425845915	0,670220127	0,999930046
ID=187	1,185735638	-0,992053909	0,914713775	-1,084551185	0,278120485	0,999930046
ID=188	2,611176558	-0,160759034	0,533149343	-0,30152721	0,763012504	0,999930046
ID=189	2,10090328	-0,989635695	0,577753672	-1,712902475	0,086730483	0,999930046
ID=190	3,155222072	0,073119865	0,48476542	0,150835562	0,880105432	0,999930046
ID=191	1,097219236	-0,118093828	0,720100704	-0,163996268	0,869734082	0,999930046
ID=193	1,454240016	0,768909988	0,605409396	1,270066162	0,204061064	0,999930046
ID=195	0,274194082	-0,079502745	1,643403312	-0,048376892	0,961415875	0,999930046
ID=199	13,08321659	0,488374247	0,34705134	1,407210379	0,159365015	0,999930046
ID=201	1,437901946	-0,105070366	0,727280501	-0,144470209	0,885129179	0,999930046
ID=202	4,081773908	-0,455953868	0,699889984	-0,651465056	0,514746327	0,999930046
ID=207	0,612145694	-0,62841893	2,656153765	-0,236589816	0,812975022	0,999930046
ID=209	1,07598717	0,417350876	0,773541188	0,539532842	0,589519242	0,999930046
ID=212	12,831266	0,116145662	0,446609996	0,260060597	0,794817031	0,999930046
ID=214	4,632413222	-0,176183865	0,396609353	-0,44422519	0,656879782	0,999930046
ID=215	0,400268318	1,187323357	1,083904695	1,095413058	0,273335716	0,999930046
ID=216	1,878305397	-0,944952399	0,556492408	-1,698050837	0,089498167	0,999930046
ID=219	0,395199547	-0,100945661	1,294005792	-0,078010208	0,937819933	0,999930046
ID=224	0,428338016	-1,323643231	1,164880745	-1,136290763	0,255834895	0,999930046
ID=225	0,394551661	0,138106247	1,026915444	0,134486483	0,893017899	0,999930046
ID=227	0,494528308	-0,046886143	0,938959834	-0,04993413	0,960174879	0,999930046
ID=231	2,229868095	0,136866622	0,532942625	0,256813053	0,797323093	0,999930046
ID=232	0,629572613	-1,209467819	1,072769525	-1,127425594	0,259562583	0,999930046
ID=233	1,529349315	-0,403569287	0,716007708	-0,563638188	0,573000392	0,999930046
ID=234	1,335591606	0,239374137	0,813590196	0,294219544	0,768590147	0,999930046
ID=235	1,61326927	0,00058926	0,740141012	0,000796145	0,999364768	0,999930046
ID=236	2,204066992	0,057326899	0,626176368	0,091550723	0,927055004	0,999930046
ID=237	1,217535739	-0,048429692	0,712509286	-0,067970612	0,945809028	0,999930046
ID=239	16,80312115	-0,120302923	0,322168097	-0,373416623	0,708838389	0,999930046
ID=241	1,80428868	-1,227443555	0,627586516	-1,955815692	0,05048687	0,999930046
ID=242	0,273985059	0,82234055	1,205984618	0,681883116	0,495312862	0,999930046
ID=243	3,906134675	0,463455336	0,490530344	0,944804622	0,344758632	0,999930046
ID=244	1,865326715	0,361571272	0,577788357	0,625784973	0,531455989	0,999930046
ID=245	0,563072515	-0,748253174	0,93159066	-0,803199523	0,421859421	0,999930046
ID=246	1,917568041	0,444951867	0,52844611	0,842000458	0,399787697	0,999930046
ID=247	0,489851502	-0,139909805	1,142605402	-0,122448051	0,902544185	0,999930046
ID=248	1,047514296	-0,191110795	0,949585425	-0,201257085	0,840497557	0,999930046
ID=249	1,036896117	0,942114084	0,738579816	1,275575182	0,202105732	0,999930046
ID=251	1,443178177	-0,494395384	0,75327522	-0,656327689	0,511613306	0,999930046
ID=252	110,8299741	-0,678892037	0,378395249	-1,794134674	0,072791676	0,999930046
ID=253	1,34948353	-0,33857343	0,766753079	-0,441567748	0,658802027	0,999930046
ID=256	0,716844083	-0,155370067	0,827728746	-0,187706501	0,851106734	0,999930046
ID=257	1,569621868	1,436369154	0,725496979	1,979841674	0,047721322	0,999930046
ID=258	0,851439368	-0,829582521	0,771927029	-1,074690339	0,28251339	0,999930046
ID=260	0,662585716	-0,063675078	0,815908653	-0,078041921	0,937794707	0,999930046
ID=261	0,630848534	1,17880704	1,044806046	1,12825442	0,259212487	0,999930046
ID=262	0,859402607	-0,085253568	0,712325831	-0,119683387	0,904733961	0,999930046
ID=263	2,796596419	-0,494220472	0,446717864	-1,106336934	0,268580711	0,999930046
ID=267	0,577412855	-0,293220397	0,804057588	-0,364675866	0,715353376	0,999930046
ID=268	1,165513437	-0,496527091	0,672504804	-0,738324973	0,460316995	0,999930046
ID=269	0,684493196	-0,289009733	0,717679082	-0,402700511	0,687168558	0,999930046
ID=270	1,292131653	-0,562424465	0,615050319	-0,914436507	0,360487526	0,999930046
ID=271	1,401967309	0,30735212	0,697230996	0,440818211	0,659344609	0,999930046
ID=272	0,626483803	-0,758281544	0,778861208	-0,973577239	0,330266492	0,999930046
ID=276	1,90102843	-0,06165595	0,48292456	-0,127672011	0,898408541	0,999930046
ID=277	1,378682636	-0,376741804	0,55429654	-0,679675548	0,496709922	0,999930046
ID=279	0,886967583	-1,693035312	0,880378816	-1,923075933	0,054470514	0,999930046
ID=280	3,081934887	0,500224026	0,435286965	1,149182187	0,250480865	0,999930046
ID=282	0,48003047	-0,605535684	0,896448536	-0,67548293	0,499368993	0,999930046
ID=284	0,716177089	0,300927885	0,790257748	0,380797134	0,703353786	0,999930046
ID=285	3,10979191	-0,103940191	0,405126655	-0,256562211	0,79751675	0,999930046
ID=286	2,31662726	0,601057744	0,436113907	1,378212742	0,168137614	0,999930046
ID=288	2,037438781	-0,347541772	0,509444636	-0,682197333	0,495114181	0,999930046
ID=289	1,388389998	-0,701246998	0,862987102	-0,812581087	0,416458279	0,999930046
ID=290	11,36608846	0,268022597	0,300246871	0,89267407	0,37203174	0,999930046
ID=291	5,008191219	-0,12638934	0,466868451	-0,270717244	0,786608513	0,999930046
ID=292	1,09322338	0,102729856	0,770376202	0,133350246	0,893916392	0,999930046
ID=293	0,449275504	-1,583887907	1,068622457	-1,482177262	0,138293135	0,999930046
ID=296	0,734300367	-1,569837097	0,913896729	-1,717740143	0,085844024	0,999930046
ID=307	2,93614541	0,252710921	0,42482204	0,594863019	0,551935039	0,999930046
ID=309	0,274695679	0,298013758	1,433953678	0,207826628	0,83536434	0,999930046
ID=310	14,77263085	0,025433256	0,442024366	0,05753813	0,954116533	0,999930046
ID=311	0,422495532	1,215467551	1,139870198	1,066321019	0,286278551	0,999930046
ID=315	0,603161391	0,737292075	0,814932388	0,904727908	0,365609553	0,999930046
ID=316	5,278897096	0,597756117	0,398670871	1,499372443	0,133777038	0,999930046
ID=325	1,672786158	0,055712354	0,643633734	0,086559096	0,93102198	0,999930046
ID=326	1,641642091	0,691478565	0,792616356	0,872400071	0,382990162	0,999930046
ID=327	0,805074772	-0,908290857	0,747370563	-1,215315268	0,224245871	0,999930046
ID=328	0,45784082	-0,963285444	1,138708477	-0,845945615	0,397583084	0,999930046
ID=331	143,8069461	0,094445239	0,264273821	0,357376447	0,720810006	0,999930046
ID=332	0,599107551	-0,823203732	0,831985494	-0,989444813	0,322445558	0,999930046
ID=333	10,91232623	-0,146453129	0,353230803	-0,414610298	0,678427208	0,999930046
ID=335	1,879507377	0,58204813	0,546199972	1,06563193	0,28659006	0,999930046
ID=336	0,396768574	0,485258122	1,035987555	0,468401498	0,639497497	0,999930046
ID=337	1,197600634	-0,205737144	0,680071185	-0,302522955	0,762253437	0,999930046
ID=338	0,571565574	-1,591680316	1,137233445	-1,399607375	0,161630924	0,999930046
ID=348	1,391581493	0,190116282	0,578906838	0,328405659	0,742604965	0,999930046
ID=355	149,9130497	0,104792243	0,26855884	0,390202173	0,696387055	0,999930046
ID=357	2,973861616	0,166287574	0,413659481	0,401991449	0,687690319	0,999930046
ID=359	2,960805748	0,177553951	0,408036568	0,435142252	0,663459176	0,999930046
ID=360	2,440559231	-0,971567726	0,577369799	-1,682747743	0,092423935	0,999930046
ID=361	14,64592791	-0,18942867	0,405345027	-0,467326986	0,640265952	0,999930046
ID=362	2,913668363	-0,276547549	0,571752532	-0,483683996	0,628610153	0,999930046
ID=363	13,33492189	0,348402475	0,360297202	0,966986347	0,333550859	0,999930046
ID=364	25,91125939	-0,253371816	0,274596693	-0,92270527	0,356160819	0,999930046
ID=366	17,54790233	0,058801368	0,238200246	0,246856873	0,805018994	0,999930046
ID=367	1,383922897	0,22418953	0,699188389	0,320642524	0,748481308	0,999930046
ID=369	5,318205599	-0,415437833	0,375456849	-1,106486231	0,26851612	0,999930046
ID=370	1,156794461	-0,324013011	0,823890562	-0,393271906	0,694118666	0,999930046
ID=371	0,758993029	-0,801813499	1,128211584	-0,710694262	0,477273716	0,999930046
ID=372	3,26227238	-0,062609631	0,443979171	-0,141019299	0,887854697	0,999930046
ID=373	1,212735589	0,172129967	0,697789105	0,246679069	0,805156607	0,999930046
ID=374	3,777325699	-0,446663003	0,389895181	-1,14559765	0,25196167	0,999930046
ID=375	57,0314284	-0,218937001	0,239215111	-0,915230645	0,36007056	0,999930046
ID=377	1,743714496	-0,065159834	0,58415521	-0,111545449	0,911183855	0,999930046
ID=378	33,12494515	0,108649116	0,215037895	0,505255673	0,613379272	0,999930046
ID=379	0,890786765	1,029417076	0,823729975	1,249702094	0,211408392	0,999930046
ID=380	16,21678448	-0,153042902	0,344788474	-0,443874762	0,657133132	0,999930046
ID=381	2,747720359	-0,355859966	0,594861023	-0,598223706	0,549690675	0,999930046
ID=382	0,618263044	-1,32165716	0,997158805	-1,325422945	0,185030911	0,999930046
ID=383	3,746828049	0,150938871	0,594732366	0,253792932	0,799655534	0,999930046
ID=384	4,764548663	-0,919270179	0,529221311	-1,737024114	0,082382915	0,999930046
ID=385	0,417803689	0,873377397	1,355109535	0,644506864	0,519246808	0,999930046
ID=386	2,198687459	0,233920859	0,617381281	0,37889205	0,704768028	0,999930046
ID=387	1,291754251	-0,103657492	0,792645179	-0,13077414	0,89595398	0,999930046
ID=389	2,385957766	-1,197714207	0,62882393	-1,904689296	0,056820471	0,999930046
ID=390	0,739368672	-0,99717176	0,839566595	-1,187722054	0,234942932	0,999930046
ID=391	4,498086399	-0,137342602	0,417262203	-0,329151793	0,742040958	0,999930046
ID=392	1,398848018	1,268072253	0,700607205	1,809961764	0,070301717	0,999930046
ID=393	5,807059741	0,201300755	0,436421874	0,461252672	0,644617336	0,999930046
ID=394	3,0160278	-0,391163373	0,455087813	-0,859533834	0,390046062	0,999930046
ID=395	2,217189964	-0,23728579	0,595191408	-0,398671396	0,690135347	0,999930046
ID=396	3,339993958	0,37058641	0,588374524	0,629847817	0,528794157	0,999930046
ID=397	1,006014843	-0,04163367	0,842232859	-0,049432493	0,960574634	0,999930046
ID=398	6,994937136	0,174186166	0,343797295	0,506653684	0,612397834	0,999930046
ID=399	14,7503681	-0,193952369	0,227267596	-0,853409695	0,393432147	0,999930046
ID=400	3,190896555	-0,394547348	0,465277809	-0,847982304	0,396447826	0,999930046
ID=401	14,51308695	-0,166700368	0,249609556	-0,667844495	0,504232861	0,999930046
ID=402	4,505888265	0,371477243	0,424934386	0,874199064	0,382009856	0,999930046
ID=403	0,687905824	-0,400475094	1,204819571	-0,332394247	0,739591582	0,999930046
ID=404	2,47291676	0,428349476	0,479377267	0,893554002	0,371560567	0,999930046
ID=405	108,1023337	-0,418681363	0,274680169	-1,524250421	0,127446167	0,999930046
ID=406	0,646160224	-0,790268815	0,925589265	-0,853800758	0,393215395	0,999930046
ID=407	2,115718009	0,72243859	0,683567909	1,056864403	0,290573476	0,999930046
ID=409	0,367074189	0,540808404	1,133236255	0,477224764	0,633202079	0,999930046
ID=413	1,095677153	-1,142453864	0,914807003	-1,248846872	0,211721087	0,999930046
ID=414	12,50721778	-0,33617603	0,289812672	-1,159976984	0,246058177	0,999930046
ID=415	0,769503634	0,375564147	0,880898056	0,42634235	0,669858403	0,999930046
ID=418	7,136505044	0,342178159	0,356426159	0,960025381	0,337042441	0,999930046
ID=419	5,912524162	0,349275274	0,323857905	1,078483091	0,280818224	0,999930046
ID=420	1,774173192	-0,283600788	0,552778623	-0,513045867	0,607919237	0,999930046
ID=421	1,886273641	0,921317634	0,580216947	1,587884736	0,112312406	0,999930046
ID=422	1,028152859	0,638506245	0,878743287	0,726612942	0,467463098	0,999930046
ID=423	1,145850116	0,263935644	0,774165215	0,34092935	0,733156769	0,999930046
ID=424	0,913150738	-0,074561307	0,876750556	-0,085042783	0,932227378	0,999930046
ID=425	21,14789689	0,451400896	0,515541795	0,875585454	0,381255436	0,999930046
ID=426	0,419052604	0,78358191	1,219006025	0,642803968	0,520351308	0,999930046
ID=428	0,461481268	0,079790793	2,645637705	0,03015938	0,975939944	0,999930046
ID=429	5,057311511	-0,065054413	0,427555418	-0,152154341	0,879065205	0,999930046
ID=431	0,696596332	-0,211162144	1,098692878	-0,192193968	0,847590271	0,999930046
ID=432	0,306647724	0,434067293	1,330084793	0,32634558	0,744162906	0,999930046
ID=435	1,38869624	-0,092781573	0,635785389	-0,14593222	0,883974896	0,999930046
ID=436	14,96360347	-0,171880502	0,436851715	-0,393452734	0,693985128	0,999930046
ID=437	0,248532316	-0,495435997	1,350885163	-0,366749159	0,713806134	0,999930046
ID=438	1,140767641	0,841632304	0,745668787	1,128694561	0,259026705	0,999930046
ID=439	2,600918481	0,241253698	0,538877505	0,447696732	0,654372081	0,999930046
ID=441	20,04862516	-0,056213972	0,241400807	-0,232865719	0,815865679	0,999930046
ID=442	24,84128431	0,339635239	0,44009326	0,771734697	0,440271576	0,999930046
ID=443	92,57565615	0,101724099	0,521597602	0,195024092	0,845374087	0,999930046
ID=444	1,944213763	1,295526955	0,688670436	1,881200191	0,059944692	0,999930046
ID=448	1,902063833	-0,612735645	0,645233305	-0,949634249	0,34229813	0,999930046
ID=450	918,6021985	0,090198458	0,462043601	0,195216334	0,845223592	0,999930046
ID=451	16,30665026	0,180409287	0,307317732	0,587044833	0,557173597	0,999930046
ID=452	1,930062098	-0,742747324	0,766294612	-0,969271233	0,332409878	0,999930046
ID=453	1,079003332	-1,19036053	0,756796966	-1,572892841	0,115743633	0,999930046
ID=454	1,795075382	1,002730095	0,592335166	1,692842419	0,090485466	0,999930046
ID=455	7,734589012	-0,471073439	0,447878	-1,051789637	0,292896075	0,999930046
ID=456	24,35562574	-0,067767058	0,26127583	-0,259369793	0,795349934	0,999930046
ID=457	3,688098273	0,267387729	0,480293261	0,556717637	0,577720365	0,999930046
ID=460	2,986976259	-0,089944325	0,485046459	-0,185434454	0,852888287	0,999930046
ID=462	0,450791642	0,045887734	1,04757755	0,043803663	0,965060907	0,999930046
ID=463	128,7373281	-0,419388363	0,309623977	-1,354508674	0,175574148	0,999930046
ID=464	0,240532525	0,311724589	1,330206386	0,234343025	0,814718686	0,999930046
ID=468	0,547258355	1,957804563	2,025855777	0,966408658	0,333839733	0,999930046
ID=469	0,897184854	-0,190082293	0,758609731	-0,250566643	0,802149174	0,999930046
ID=472	12,34887623	-0,127682913	0,295971752	-0,431402363	0,666175832	0,999930046
ID=474	0,550775353	0,285917196	0,945653433	0,302348816	0,762386168	0,999930046
ID=475	1,554548126	-0,707485548	0,81458637	-0,86852122	0,385109061	0,999930046
ID=476	7,33006571	-0,139893763	0,423620324	-0,330233831	0,741223286	0,999930046
ID=477	2,142257393	-0,695669501	0,535711771	-1,298589163	0,194084959	0,999930046
ID=478	1,239466603	-1,022915109	0,602392657	-1,69808695	0,089491352	0,999930046
ID=479	1,317080558	-0,36511853	0,604177252	-0,604323531	0,545628565	0,999930046
ID=480	0,568372542	0,297020176	0,855407654	0,347226466	0,72842119	0,999930046

	DICKSON ET AL. 2018		TUULARI ET AL. 2023			ROBERTS ET AL. 2018		TUULARI ET AL. 2023	
**SPERM EPIGENOME ANALYSIS**	sncRNA		sncRNA			DNAme		DNAme	
**SAMPLE SIZE**	N = 28		N = 30			N = 34		N = 55	
**SOURCE POPULATION**	Infertility treatment clinic		Community sample			Community sample		Community sample	
**MEAN AGE OF THE PARTICIPANTS**	32.4 years		38.5 years			25.7 years		39.5 years	
**DISCOVERY ANALYSIS**	Low	High	Low		High	Low	High	Low	High
**CONTROL VS. CASE (N)**	5	5	16		14	12	17	30	25
**MULTIVARIATE STATISTICS**	No		Yes			Yes		Yes	
			A	B	C	A	B		
**SEMEN SAMPLE VOLUME**	X		*		*	*	*	*	
**SPERM CONCENTRATION**	X		*		*			*	
**SPERM MORPHOLOGY**	X								
**AGE**				*	*	*	*	*	
**BMI**	X			*	*		*	*	
**SMOKING**	X			*	*		*	*	
**ALCOHOL USE**	X			*	*			*	
**DRUG USE**	X						*		
**DEPRESSIVE SYMPTOMS**				*	*			*	
**ANXIETY SYMPTOMS**				*	*			*	
**POST-TRAUMATIC STRESS**							*		
**ADULTHOOD TRAUMA**							*		
**MEASURE OF EARLY LIFE STRESS EXPOSURE**	Adverse child experiences (ACE) questionnaire		Trauma and Distress Scale (TADS) questionnaire			Childhood Trauma Questionnaire (CTQ)		Trauma and Distress Scale (TADS) questionnaire	

**MAIN FINDINGS**	A negative correlation between levels of multiple miRNAs of the miR-449/34 family and ACE scores of Caucasian males.<br><br>The authors also performed studies in mice and found the same sperm miRNA family are also reduced in mice exposed to adolescent chronic social instability.	A replication of the negative correlation between CME miR-34 but not with miR-449.<br><br>Discovery analyses across a wider set of small non-coding RNAs revealed multiple associations with dozens of implicated sncRNAs.		13 differentially methylated DNA regions associated with by childhood abuse (high abuse vs. no abuse); 3 negative associations and 10 positive associations.<br><br>The implicated sites included genes associated with neuronal function (*MAPT*, *CLU*), fat cell regulation (*PRDM16*), and immune function (*SDK1*).	3 differentially methylated DNA regions associated with by childhood abuse (high abuse vs. no abuse); all associations were negative.<br><br>The implicated sites included genes associated with neuronal function (*CRTC1*, GBX2).

Supplementary Table 5. Table comparing the data, sample sizes and statistical analyses in published studies on childhood trauma and sperm epigenome. (X = reported only in univariate linear regression model across the whole sample: miRNA (miR-449a / miR-34c) ~ X), (* = included in multivariate testing associations between early life stress and sperm epigenome that statistically adjusted for these potential confounders in the same model).

Footnotes:

Jawaid et al. report change in sperm miRNAs form men aged 21-50 years (n = 23, control n = 35) exposed to two or more significant traumatic events in childhood (Mann Whitney U test; no covariates reported). The traumatic events were defined from childhood trauma questionnaire (CTQ). The study was available as a preprint at the time of writing and did not perform discovery analyses, so the data are not included in the

table. **Reference:** Distinct microRNA signature in human serum and germline after childhood trauma; Ali Jawaid, et al 2020, medRxiv 2020.08.11.20168393; doi: https://doi.org/10.1101/2020.08.11.20168393

Methodological differences to prior work in sncRNA analyses: In contrast to the earlier study by Dickson et al. that used miRNA microarrays for initial screening of differentially expressed miRNAs (Dickson et al., 2018), we chose a genome-wide high throughput approach and used small RNA-seq to identify differentially expressed miRNAs in men exposed to high CME compared to men with low CME.

Methodological differences to prior work in DNA methylation analyses: The previous study by Roberts et al. (8) identified differentially methylated sperm DNA using methylation BeadChips, but we chose a sequencing-based approach and used RRBS. Of note, these methods have been shown to cover different CpG loci (33). Our approach was validated separately with pyrosequencing as detailed in the manuscript.

Supplementary Table 6. Multivariate statistics of miRNA

	Variables		Low / High CME	Low / High CME	Low / High CME	Low / High CME	Variables		TADS sum score	TADS sum score	TADS sum score	TADS sum score
**A) Controlling for**			No covariates	A	B	C			No covariates	A	B	C
semen sample volume	1. Low [0] / High [1] CME	Spearman's rho	—	—	—	—	1. TADS sum score		—	—	—	—
sperm concentration		p-value	—	—	—	—			—	—	—	—
	2. TADS sum score	Spearman's rho	0.864	0.861	0.819	0.812	2. Low [0] / High [1] CME	Spearman's rho	0.864	0.861	0.819	0.812
**B) Controlling for**		p-value	< .001	< .001	< .001	< .001		p-value	< .001	< .001	< .001	< .001
age, BMI,	3. hsa-miR-1-3p	Spearman's rho	0.641	0.623	0.585	0.576	3. hsa-miR-1-3p	Spearman's rho	0.581	0.563	0.532	0.529
smoking (yes / no),		p-value	< .001	< .001	0.005	0.010		p-value	0.001	0.003	0.013	0.020
average alcohol use / day	4. hsa-miR-101-3p	Spearman's rho	-0.622	-0.611	-0.609	-0.605	4. hsa-miR-101-3p	Spearman's rho	-0.671	-0.664	-0.701	-0.692
depressive symptoms		p-value	< .001	0.001	0.003	0.006		p-value	< .001	< .001	< .001	0.001
anxiety symptoms	5. hsa-miR-122-3p	Spearman's rho	0.634	0.598	0.484	0.487	5. hsa-miR-122-3p	Spearman's rho	0.572	0.523	0.384	0.394
		p-value	< .001	0.002	0.026	0.035		p-value	0.002	0.007	0.086	0.095
**C) Controlling for**	6. hsa-miR-1251-5p	Spearman's rho	-0.373	-0.373	-0.604	-0.590	6. hsa-miR-1251-5p	Spearman's rho	-0.531	-0.544	-0.796	-0.796
covariates in A + B.		p-value	0.055	0.066	0.004	0.008		p-value	0.004	0.005	< .001	< .001
	7. hsa-miR-1296-5p	Spearman's rho	-0.536	-0.543	-0.675	-0.684	7. hsa-miR-1296-5p	Spearman's rho	-0.521	-0.545	-0.644	-0.677
		p-value	0.004	0.005	< .001	0.001		p-value	0.005	0.005	0.002	0.001
	8. hsa-miR-1307-5p	Spearman's rho	-0.479	-0.555	-0.605	-0.650	8. hsa-miR-1307-5p	Spearman's rho	-0.539	-0.617	-0.656	-0.702
		p-value	0.012	0.004	0.004	0.003		p-value	0.004	0.001	0.001	< .001
	9. hsa-miR-134-5p	Spearman's rho	0.479	0.450	0.162	0.161	9. hsa-miR-134-5p	Spearman's rho	0.409	0.381	0.133	0.136
		p-value	0.011	0.024	0.483	0.510		p-value	0.034	0.060	0.565	0.580
	10. hsa-miR-141-3p	Spearman's rho	-0.612	-0.611	-0.641	-0.642	10. hsa-miR-141-3p	Spearman's rho	-0.669	-0.663	-0.727	-0.717
		p-value	< .001	0.001	0.002	0.003		p-value	< .001	< .001	< .001	< .001
	11. hsa-miR-141-5p	Spearman's rho	-0.609	-0.622	-0.659	-0.677	11. hsa-miR-141-5p	Spearman's rho	-0.672	-0.682	-0.743	-0.745
		p-value	< .001	< .001	0.001	0.001		p-value	< .001	< .001	< .001	< .001
	12. hsa-miR-148a-3p	Spearman's rho	-0.574	-0.543	-0.570	-0.556	12. hsa-miR-148a-3p	Spearman's rho	-0.754	-0.736	-0.774	-0.762
		p-value	0.002	0.005	0.007	0.013		p-value	< .001	< .001	< .001	< .001
	13. hsa-miR-18a-3p	Spearman's rho	0.607	0.572	0.567	0.552	13. hsa-miR-18a-3p	Spearman's rho	0.533	0.503	0.564	0.569
		p-value	< .001	0.003	0.007	0.014		p-value	0.004	0.010	0.008	0.011
	14. hsa-miR-196b-5p	Spearman's rho	-0.536	-0.534	-0.582	-0.585	14. hsa-miR-196b-5p	Spearman's rho	-0.617	-0.625	-0.671	-0.687
		p-value	0.004	0.006	0.006	0.009		p-value	< .001	< .001	< .001	0.001
	15. hsa-miR-19b-3p	Spearman's rho	-0.569	-0.623	-0.588	-0.643	15. hsa-miR-19b-3p	Spearman's rho	-0.633	-0.671	-0.701	-0.729
		p-value	0.002	< .001	0.005	0.003		p-value	< .001	< .001	< .001	< .001
	16. hsa-miR-200a-3p	Spearman's rho	-0.507	-0.509	-0.538	-0.536	16. hsa-miR-200a-3p	Spearman's rho	-0.610	-0.609	-0.675	-0.665
		p-value	0.007	0.009	0.012	0.018		p-value	< .001	0.001	< .001	0.002
	17. hsa-miR-21-5p	Spearman's rho	-0.670	-0.677	-0.656	-0.663	17. hsa-miR-21-5p	Spearman's rho	-0.646	-0.645	-0.686	-0.688
		p-value	< .001	< .001	0.001	0.002		p-value	< .001	< .001	< .001	0.001
	18. hsa-miR-210-3p	Spearman's rho	-0.335	-0.400	-0.459	-0.537	18. hsa-miR-210-3p	Spearman's rho	-0.324	-0.394	-0.478	-0.596
		p-value	0.088	0.048	0.036	0.018		p-value	0.100	0.051	0.028	0.007
	19. hsa-miR-26b-5p	Spearman's rho	-0.603	-0.656	-0.643	-0.685	19. hsa-miR-26b-5p	Spearman's rho	-0.524	-0.553	-0.602	-0.611
		p-value	< .001	< .001	0.002	0.001		p-value	0.005	0.004	0.004	0.005
	20. hsa-miR-29b-3p	Spearman's rho	-0.421	-0.505	-0.660	-0.689	20. hsa-miR-29b-3p	Spearman's rho	-0.410	-0.474	-0.682	-0.710
		p-value	0.029	0.010	0.001	0.001		p-value	0.034	0.017	< .001	< .001
	21. hsa-miR-29c-3p	Spearman's rho	-0.737	-0.757	-0.716	-0.739	21. hsa-miR-29c-3p	Spearman's rho	-0.729	-0.755	-0.737	-0.775
		p-value	< .001	< .001	< .001	< .001		p-value	< .001	< .001	< .001	< .001
	22. hsa-miR-30e-5p	Spearman's rho	-0.651	-0.674	-0.622	-0.647	22. hsa-miR-30e-5p	Spearman's rho	-0.519	-0.509	-0.503	-0.495
		p-value	< .001	< .001	0.003	0.003		p-value	0.005	0.009	0.020	0.031
	23. hsa-miR-32-5p	Spearman's rho	-0.699	-0.718	-0.714	-0.736	23. hsa-miR-32-5p	Spearman's rho	-0.662	-0.672	-0.723	-0.726
		p-value	< .001	< .001	< .001	< .001		p-value	< .001	< .001	< .001	< .001
	24. hsa-miR-323a-3p	Spearman's rho	0.546	0.492	0.209	0.181	24. hsa-miR-323a-3p	Spearman's rho	0.507	0.469	0.213	0.223
		p-value	0.003	0.012	0.363	0.458		p-value	0.007	0.018	0.355	0.358
	25. hsa-miR-323b-3p	Spearman's rho	0.557	0.484	0.402	0.366	25. hsa-miR-323b-3p	Spearman's rho	0.545	0.460	0.435	0.374
		p-value	0.003	0.014	0.071	0.123		p-value	0.003	0.021	0.049	0.115
	26. hsa-miR-34c-5p	Spearman's rho	-0.469	-0.422	-0.443	-0.420	26. hsa-miR-34c-5p	Spearman's rho	-0.454	-0.398	-0.455	-0.409
		p-value	0.014	0.035	0.044	0.073		p-value	0.017	0.049	0.038	0.082
	27. hsa-miR-3615	Spearman's rho	0.224	0.101	0.131	0.030	27. hsa-miR-3615	Spearman's rho	0.317	0.194	0.328	0.232
		p-value	0.261	0.631	0.572	0.904		p-value	0.107	0.352	0.147	0.340
	28. hsa-miR-362-3p	Spearman's rho	-0.568	-0.656	-0.628	-0.693	28. hsa-miR-362-3p	Spearman's rho	-0.589	-0.658	-0.695	-0.734
		p-value	0.002	< .001	0.002	0.001		p-value	0.001	< .001	< .001	< .001
	29. hsa-miR-370-3p	Spearman's rho	0.354	0.452	0.101	0.209	29. hsa-miR-370-3p	Spearman's rho	0.165	0.259	-0.100	-0.002
		p-value	0.070	0.023	0.662	0.391		p-value	0.412	0.212	0.665	0.995
	30. hsa-miR-374a-5p	Spearman's rho	-0.737	-0.760	-0.699	-0.733	30. hsa-miR-374a-5p	Spearman's rho	-0.665	-0.661	-0.644	-0.643
		p-value	< .001	< .001	< .001	< .001		p-value	< .001	< .001	0.002	0.003
	31. hsa-miR-3909	Spearman's rho	0.442	0.450	0.556	0.568	31. hsa-miR-3909	Spearman's rho	0.466	0.455	0.653	0.639
		p-value	0.021	0.024	0.009	0.011		p-value	0.014	0.022	0.001	0.003
	32. hsa-miR-409-3p	Spearman's rho	0.575	0.559	0.511	0.495	32. hsa-miR-409-3p	Spearman's rho	0.532	0.517	0.577	0.560
		p-value	0.002	0.004	0.018	0.031		p-value	0.004	0.008	0.006	0.013
	33. hsa-miR-429	Spearman's rho	-0.555	-0.520	-0.560	-0.543	33. hsa-miR-429	Spearman's rho	-0.681	-0.657	-0.698	-0.686
		p-value	0.003	0.008	0.008	0.016		p-value	< .001	< .001	< .001	0.001
	34. hsa-miR-433-3p	Spearman's rho	0.732	0.792	0.726	0.769	34. hsa-miR-433-3p	Spearman's rho	0.477	0.536	0.410	0.460
		p-value	< .001	< .001	< .001	< .001		p-value	0.012	0.006	0.065	0.048
	35. hsa-miR-452-5p	Spearman's rho	-0.502	-0.525	-0.533	-0.551	35. hsa-miR-452-5p	Spearman's rho	-0.535	-0.569	-0.572	-0.602
		p-value	0.008	0.007	0.013	0.015		p-value	0.004	0.003	0.007	0.006
	36. hsa-miR-509-3p	Spearman's rho	0.526	0.465	0.539	0.525	36. hsa-miR-509-3p	Spearman's rho	0.531	0.457	0.548	0.503
		p-value	0.005	0.019	0.012	0.021		p-value	0.004	0.022	0.010	0.028
	37. hsa-miR-511-5p	Spearman's rho	0.447	0.385	0.295	0.269	37. hsa-miR-511-5p	Spearman's rho	0.486	0.417	0.354	0.322
		p-value	0.019	0.057	0.194	0.266		p-value	0.010	0.038	0.115	0.179
	38. hsa-miR-532-3p	Spearman's rho	0.440	0.396	0.310	0.285	38. hsa-miR-532-3p	Spearman's rho	0.515	0.482	0.448	0.422
		p-value	0.022	0.050	0.172	0.237		p-value	0.006	0.015	0.042	0.072
	39. hsa-miR-548a-3p	Spearman's rho	-0.550	-0.508	-0.496	-0.480	39. hsa-miR-548a-3p	Spearman's rho	-0.497	-0.442	-0.494	-0.450
		p-value	0.003	0.010	0.022	0.038		p-value	0.008	0.027	0.023	0.053
	40. hsa-miR-548ah-3p	Spearman's rho	-0.579	-0.558	-0.534	-0.548	40. hsa-miR-548ah-3p	Spearman's rho	-0.503	-0.468	-0.466	-0.433
		p-value	0.002	0.004	0.013	0.015		p-value	0.007	0.018	0.033	0.064
	41. hsa-miR-576-5p	Spearman's rho	0.544	0.559	0.561	0.587	41. hsa-miR-576-5p	Spearman's rho	0.731	0.752	0.772	0.787
		p-value	0.003	0.004	0.008	0.008		p-value	< .001	< .001	< .001	< .001
	42. hsa-miR-582-3p	Spearman's rho	-0.528	-0.501	-0.568	-0.565	42. hsa-miR-582-3p	Spearman's rho	-0.683	-0.658	-0.762	-0.750
		p-value	0.005	0.011	0.007	0.012		p-value	< .001	< .001	< .001	< .001
	43. hsa-miR-618	Spearman's rho	0.517	0.530	0.623	0.621	43. hsa-miR-618	Spearman's rho	0.559	0.572	0.688	0.688
		p-value	0.006	0.006	0.003	0.005		p-value	0.002	0.003	< .001	0.001
	44. hsa-miR-660-3p	Spearman's rho	-0.761	-0.757	-0.727	-0.731	44. hsa-miR-660-3p	Spearman's rho	-0.824	-0.821	-0.814	-0.814
		p-value	< .001	< .001	< .001	< .001		p-value	< .001	< .001	< .001	< .001
	45. hsa-miR-660-5p	Spearman's rho	-0.483	-0.512	-0.608	-0.641	45. hsa-miR-660-5p	Spearman's rho	-0.558	-0.580	-0.761	-0.775
		p-value	0.011	0.009	0.003	0.003		p-value	0.002	0.002	< .001	< .001
	46. hsa-miR-760	Spearman's rho	0.601	0.616	0.535	0.553	46. hsa-miR-760	Spearman's rho	0.510	0.521	0.432	0.444
		p-value	< .001	0.001	0.012	0.014		p-value	0.007	0.008	0.051	0.057
	47. hsa-miR-888-5p	Spearman's rho	-0.440	-0.471	-0.527	-0.546	47. hsa-miR-888-5p	Spearman's rho	-0.535	-0.561	-0.718	-0.724
		p-value	0.022	0.017	0.014	0.016		p-value	0.004	0.004	< .001	< .001
	48. hsa-miR-891b	Spearman's rho	-0.422	-0.424	-0.464	-0.473	48. hsa-miR-891b	Spearman's rho	-0.540	-0.534	-0.659	-0.653
		p-value	0.028	0.035	0.034	0.041		p-value	0.004	0.006	0.001	0.002
	49. hsa-miR-935	Spearman's rho	0.529	0.511	0.392	0.393	49. hsa-miR-935	Spearman's rho	0.401	0.359	0.276	0.266
		p-value	0.005	0.009	0.079	0.096		p-value	0.038	0.078	0.225	0.271

Supplementary Table 7. Multivariate statistics of tsRNA

		Variables		Low / High CME	Low / High CME	Low / High CME	Low / High CME
**A) Controlling for**				No covariates	A	B	C
semen sample volume		1. Low [0] / High [1] CME	Spearman's rho	—	—	—	—
sperm concentration			p-value	—	—	—	—
**B) Controlling for**		2. TADS sum score	Spearman's rho	0.864	0.861	0.819	0.812
age, BMI,			p-value	< .001	< .001	< .001	< .001
smoking (yes / no),		3. mature-mt_tRNA-Gln-TTG_5_end	Spearman's rho	0.182	0.187	0.098	0.135
average alcohol use / day			p-value	0.364	0.371	0.672	0.582
depressive symptoms		4. mature-mt_tRNA-Gln-TTG_CCA_end	Spearman's rho	0.443	0.489	0.449	0.502
anxiety symptoms			p-value	0.021	0.013	0.041	0.029
		5. mature-mt_tRNA-Leu-TAA_5_end	Spearman's rho	0.345	0.365	0.379	0.416
**C) Controlling for**			p-value	0.078	0.073	0.090	0.077
covariates in A + B.		6. mature-mt_tRNA-Lys-TTT_CCA_end	Spearman's rho	0.208	0.268	0.136	0.228
			p-value	0.297	0.194	0.558	0.347
		7. mature-mt_tRNA-Thr-TGT_3_end	Spearman's rho	-0.330	-0.308	-0.477	-0.562
			p-value	0.093	0.134	0.029	0.012
		8. mature-mt_tRNA-Trp-TCA_CCA_end	Spearman's rho	0.048	0.118	0.059	0.151
			p-value	0.813	0.576	0.798	0.537
		9. mature-tRNA-Arg-CCG_3_end	Spearman's rho	-0.316	-0.177	-0.016	0.114
			p-value	0.109	0.398	0.944	0.641
		10. mature-tRNA-Arg-CCT_3_end	Spearman's rho	-0.507	-0.415	-0.373	-0.319
			p-value	0.007	0.039	0.096	0.182
		11. mature-tRNA-Asp-GTC	Spearman's rho	-0.622	-0.556	-0.588	-0.610
			p-value	< .001	0.004	0.005	0.006
		12. mature-tRNA-Asp-GTC_3_end	Spearman's rho	-0.402	-0.328	-0.434	-0.393
			p-value	0.038	0.109	0.049	0.096
		13. mature-tRNA-Asp-GTC_CCA_end	Spearman's rho	-0.737	-0.694	-0.713	-0.716
			p-value	< .001	< .001	< .001	< .001
		14. mature-tRNA-Glu-TTC_CCA_end	Spearman's rho	-0.756	-0.730	-0.724	-0.748
			p-value	< .001	< .001	< .001	< .001
		15. mature-tRNA-Gly-CCC_5_end	Spearman's rho	-0.478	-0.447	-0.525	-0.511
			p-value	0.012	0.025	0.014	0.025
		16. mature-tRNA-Gly-GCC_5_end	Spearman's rho	-0.545	-0.522	-0.528	-0.524
			p-value	0.003	0.007	0.014	0.021
		17. mature-tRNA-Leu-TAA_5_end	Spearman's rho	0.249	0.203	-0.040	-0.051
			p-value	0.211	0.329	0.862	0.835
		18. mature-tRNA-Lys-CTT	Spearman's rho	-0.345	-0.292	-0.070	-0.053
			p-value	0.078	0.157	0.762	0.828
		19. mature-tRNA-Lys-TTT_5_end	Spearman's rho	-0.373	-0.410	-0.570	-0.572
			p-value	0.055	0.042	0.007	0.011
		20. mature-tRNA-Met-CAT	Spearman's rho	-0.545	-0.593	-0.597	-0.638
			p-value	0.003	0.002	0.004	0.003
		21. mature-tRNA-Phe-GAA_5_end	Spearman's rho	-0.460	-0.468	-0.540	-0.552
			p-value	0.016	0.018	0.012	0.014
		22. mature-tRNA-SeC-TCA	Spearman's rho	-0.498	-0.481	-0.570	-0.564
			p-value	0.008	0.015	0.007	0.012
		23. mature-tRNA-Val-AAC	Spearman's rho	-0.373	-0.419	-0.434	-0.457
			p-value	0.055	0.037	0.049	0.049

Variables		TADS sum score	TADS sum score	TADS sum score	TADS sum score
		No covariates	A	B	C
1. TADS sum score	Spearman's rho	—	—	—	—
	p-value	—	—	—	—
2. Low [0] / High [1] CME	Spearman's rho	0.864	0.861	0.819	0.812
	p-value	< .001	< .001	< .001	< .001
3. mature-mt_tRNA-Gln-TTG_5_end	Spearman's rho	0.160	0.190	0.063	0.144
	p-value	0.425	0.363	0.785	0.558
4. mature-mt_tRNA-Gln-TTG_CCA_end	Spearman's rho	0.567	0.635	0.568	0.649
	p-value	0.002	< .001	0.007	0.003
5. mature-mt_tRNA-Leu-TAA_5_end	Spearman's rho	0.441	0.486	0.454	0.520
	p-value	0.021	0.014	0.039	0.022
6. mature-mt_tRNA-Lys-TTT_CCA_end	Spearman's rho	0.221	0.320	0.128	0.287
	p-value	0.269	0.119	0.579	0.233
7. mature-mt_tRNA-Thr-TGT_3_end	Spearman's rho	-0.367	-0.304	-0.534	-0.552
	p-value	0.060	0.140	0.013	0.014
8. mature-mt_tRNA-Trp-TCA_CCA_end	Spearman's rho	0.006	0.096	-0.031	0.105
	p-value	0.977	0.648	0.894	0.668
9. mature-tRNA-Arg-CCG_3_end	Spearman's rho	-0.268	-0.098	-0.005	0.164
	p-value	0.177	0.642	0.981	0.502
10. mature-tRNA-Arg-CCT_3_end	Spearman's rho	-0.465	-0.368	-0.300	-0.228
	p-value	0.015	0.070	0.186	0.347
11. mature-tRNA-Asp-GTC	Spearman's rho	-0.718	-0.665	-0.713	-0.747
	p-value	< .001	< .001	< .001	< .001
12. mature-tRNA-Asp-GTC_3_end	Spearman's rho	-0.381	-0.287	-0.431	-0.373
	p-value	0.050	0.165	0.051	0.116
13. mature-tRNA-Asp-GTC_CCA_end	Spearman's rho	-0.717	-0.660	-0.694	-0.680
	p-value	< .001	< .001	< .001	0.001
14. mature-tRNA-Glu-TTC_CCA_end	Spearman's rho	-0.613	-0.577	-0.545	-0.583
	p-value	< .001	0.003	0.011	0.009
15. mature-tRNA-Gly-CCC_5_end	Spearman's rho	-0.486	-0.438	-0.587	-0.551
	p-value	0.010	0.028	0.005	0.015
16. mature-tRNA-Gly-GCC_5_end	Spearman's rho	-0.565	-0.525	-0.604	-0.575
	p-value	0.002	0.007	0.004	0.010
17. mature-tRNA-Leu-TAA_5_end	Spearman's rho	0.053	0.003	-0.252	-0.246
	p-value	0.792	0.989	0.271	0.310
18. mature-tRNA-Lys-CTT	Spearman's rho	-0.330	-0.280	-0.076	-0.047
	p-value	0.093	0.175	0.744	0.847
19. mature-tRNA-Lys-TTT_5_end	Spearman's rho	-0.330	-0.351	-0.540	-0.523
	p-value	0.093	0.086	0.012	0.022
20. mature-tRNA-Met-CAT	Spearman's rho	-0.671	-0.723	-0.746	-0.775
	p-value	< .001	< .001	< .001	< .001
21. mature-tRNA-Phe-GAA_5_end	Spearman's rho	-0.609	-0.631	-0.663	-0.683
	p-value	< .001	< .001	0.001	0.001
22. mature-tRNA-SeC-TCA	Spearman's rho	-0.594	-0.585	-0.648	-0.646
	p-value	0.001	0.002	0.001	0.003
23. mature-tRNA-Val-AAC	Spearman's rho	-0.547	-0.599	-0.656	-0.675
	p-value	0.003	0.002	0.001	0.002

Supplementary Table 8. Multivariate statistics of the DNA methylation levels

ID	chr	start	end	direction	[illegible]h_diff_perc[illegible]	[illegible]taCoefficie[illegible]	statistic	p.value	q.value	width	CpGs	CpG.Island	CpG.Shore	CpG.Shelf	Open.Sea	annotation	geneId	[illegible]istanceToTS[illegible]	ENSEMBL	[illegible]geneSymbo[illegible]	gene
chr19:187[illegible]	19	18777319	18778224	Hypomethy	19	-0,59763	-21,845	3,62E-05	0,034391	906	67	Yes	Yes	No	No	3' UTR	23373	93641	ENSG0000(	CRTC1	CREB regulated transcription coactivator 1
chr16:633[illegible]	16	633127	633991	Hypomethy	18	-0,56785	-17,8397	3,62E-05	0,034391	865	79	Yes	No	No	No	Promoter	117166	2142	ENSG0000(	WFIKKN1	WAP, follistatin/kazal, immunoglobulin, kunitz and netrin domain containing 1
chr2:2361[illegible]	2	2,36E+08	2,36E+08	Hypomethy	21	-0,6576	-17,0464	3,62E-05	0,034391	1837	54	Yes	Yes	No	No	Distal Inter	2637	6612	ENSG0000(	GBX2	gastrulation brain homeobox 2
chr8:1435[illegible]	8	1,44E+08	1,44E+08	Hypomethy	17	-0,53872	-13,4417	0,000181	0,114636	591	37	Yes	Yes	No	No	Promoter	10031075[illegible]	0	ENSG0000(	LOC10031[illegible]	uncharacterized LOC100310756
chr10:473[illegible]	10	47348873	47350323	Hypomethy	15	-0,48563	-13,1042	0,000217	0,114636	1440	53	Yes	Yes	No	No	Promoter	5949	521	ENSG0000(	RBP3	retinol binding protein 3
chr19:357[illegible]	19	35784156	35785100	Hypomethy	17	-0,52434	-12,5801	0,000326	0,114636	945	41	No	No	No	Yes	Promoter	115703	0	ENSG0000(	ARHGAP33	Rho GTPase activating protein 33
chr22:197[illegible]	22	19730768	19731227	Hypomethy	13	-0,40092	-12,5642	0,000326	0,114636	460	18	Yes	No	No	No	Distal Inter	2812	7229	ENSG0000(	GP1BB	glycoprotein Ib platelet subunit beta
chr7:1746[illegible]	7	1746069	1747064	Hypomethy	14	-0,43191	-12,3889	0,000362	0,114636	996	75	Yes	No	No	No	Exon	392617	80324	ENSG0000(	ELFN1	extracellular leucine rich repeat and fibronectin type III domain containing 1
chr8:2258[illegible]	8	22589123	22589913	Hypomethy	18	-0,5648	-12,2977	0,000362	0,114636	791	27	No	No	No	Yes	Promoter	541565	0	ENSG0000(	C8orf58	chromosome 8 open reading frame 58
chr12:486[illegible]	12	48641732	48642363	Hypomethy	16	-0,50317	-11,84	0,00047	0,134124	632	22	No	No	No	Yes	Distal Inter	677815	12155	ENSG0000(	SNORA2C	small nucleolar RNA, H/ACA box 2C
chr5:1917[illegible]	5	191754	192606	Hypomethy	12	-0,36157	-11,6553	0,000543	0,14069	853	56	Yes	Yes	No	No	Promoter	389257	259	ENSG0000(	LRRC14B	leucine rich repeat containing 14B
chr16:101[illegible]	16	1019306	1020485	Hypomethy	12	-0,3793	-10,9118	0,000724	0,162695	1180	43	Yes	Yes	No	No	Distal Inter	30812	37220	ENSG0000(	SOX8	SRY-box transcription factor 8
chr12:132[illegible]	12	1,32E+08	1,32E+08	Hypomethy	11	-0,3387	-10,8241	0,00076	0,162695	860	33	Yes	Yes	No	No	Distal Inter	10192841[illegible]	69925	ENSG0000(	LOC10192[illegible]	uncharacterized LOC101928416
chrX:3310[illegible]	X	331070	331931	Hypomethy	15	-0,45604	-10,3432	0,000904	0,162695	862	47	Yes	No	No	No	Distal Inter	28227	9780	ENSG0000(	PPP2R3B	protein phosphatase 2 regulatory subunit B"beta
chr14:104[illegible]	14	1,05E+08	1,05E+08	Hypomethy	17	-0,52588	-10,3167	0,000904	0,162695	921	24	Yes	Yes	No	No	Distal Inter	10192963[illegible]	4752	ENSG0000(	LINC02280	long intergenic non-protein coding RNA 2280
chr22:487[illegible]	22	48779597	48780016	Hypomethy	10	-0,32713	-10,2301	0,000941	0,162695	420	11	No	No	No	Yes	Intron	25817	290005	ENSG0000(	TAFA5	TAFA chemokine like family member 5
chr19:466[illegible]	19	46623004	46624153	Hypomethy	9	-0,27116	-10,1788	0,001013	0,162695	1150	49	Yes	Yes	No	No	Promoter	5739	936	ENSG0000(	PTGIR	prostaglandin I2 receptor
chr19:522[illegible]	19	5222502	5223288	Hypomethy	10	-0,3139	-10,1289	0,001049	0,162695	787	62	Yes	No	No	No	Exon	5802	117515	ENSG0000(	PTPRS	protein tyrosine phosphatase receptor type S
chr16:898[illegible]	16	89850192	89850683	Hypomethy	16	-0,49242	-10,0946	0,001158	0,162695	492	27	Yes	Yes	No	No	3' UTR	84501	32013	ENSG0000(	SPIRE2	spire type actin nucleation factor 2
chr7:5610[illegible]	7	5610452	5611508	Hypomethy	12	-0,37918	-9,92087	0,001302	0,162695	1057	16	No	No	Yes	No	Distal Inter	6624	7580	ENSG0000(	FSCN1	fascin actin-bundling protein 1
chr2:2397[illegible]	2	2,4E+08	2,4E+08	Hypomethy	13	-0,39595	-9,86614	0,001338	0,162695	1424	24	No	No	No	Yes	Exon	150935	-26311		LOC15093[illegible]	uncharacterized LOC150935
chr19:178[illegible]	19	17869654	17870422	Hypomethy	12	-0,39165	-9,83837	0,001338	0,162695	769	37	No	Yes	Yes	No	Promoter	6528	-1523	ENSG0000(	SLC5A5	solute carrier family 5 member 5
chr21:453[illegible]	21	45306352	45307605	Hypomethy	11	-0,34766	-9,73012	0,001447	0,162695	1254	29	No	No	Yes	Yes	Distal Inter	642852	18265	ENSG0000(	LINC00205	long intergenic non-protein coding RNA 205
chr19:271[illegible]	19	2716692	2718145	Hypomethy	9	-0,28377	-9,72168	0,001447	0,162695	1454	53	Yes	Yes	No	No	5' UTR	148252	3227	ENSG0000(	DIRAS1	DIRAS family GTPase 1
chr22:493[illegible]	22	49301955	49302925	Hypomethy	10	-0,32276	-9,70852	0,001447	0,162695	971	37	Yes	Yes	No	No	Distal Inter	12490514[illegible]	-69999	ENSG0000(	LOC12490[illegible]	uncharacterized LOC124905143
chr3:4741[illegible]	3	47417265	47418824	Hypomethy	16	-0,50488	-9,6581	0,001483	0,162695	1560	64	Yes	Yes	No	No	3' UTR	22937	57117	ENSG0000(	SCAP	SREBF chaperone
chr21:433[illegible]	21	43361847	43362631	Hypomethy	9	-0,29564	-9,57611	0,001556	0,164312	785	32	Yes	Yes	No	No	Promoter	10192839[illegible]	0	ENSG0000(	LINC01679	long intergenic non-protein coding RNA 1679
chr16:887[illegible]	16	88736874	88737741	Hypomethy	16	-0,50397	-9,4847	0,0017	0,173183	868	19	Yes	Yes	No	No	Exon	10028958[illegible]	5694		LOC10028[illegible]	uncharacterized LOC100289580
chrX:3390[illegible]	X	339080	341564	Hypomethy	13	-0,42245	-9,46031	0,001773	0,174326	2485	44	Yes	No	No	No	Exon	28227	45343	ENSG0000(	PPP2R3B	protein phosphatase 2 regulatory subunit B"beta
chr13:111[illegible]	13	1,12E+08	1,12E+08	Hypomethy	12	-0,3665	-9,31639	0,001953	0,185711	2262	32	Yes	Yes	No	No	Intron	10272448[illegible]	33759		LOC10272[illegible]	uncharacterized LOC102724489
chr11:242[illegible]	11	2420232	2420873	Hypomethy	15	-0,4731	-9,06875	0,002171	0,193449	642	19	Yes	Yes	No	No	Promoter	29850	2139	ENSG0000(	TRPM5	transient receptor potential cation channel subfamily M member 5
chr19:761[illegible]	19	7615345	7616101	Hypomethy	17	-0,52494	-9,03593	0,002171	0,193449	757	31	Yes	Yes	No	No	Exon	57662	19482	ENSG0000(	CAMSAP3	calmodulin regulated spectrin associated protein family member 3
chr11:146[illegible]	11	1460258	1461161	Hypomethy	16	-0,496	-8,87664	0,002532	0,218851	904	44	Yes	Yes	No	No	3' UTR	9024	70359	ENSG0000(	BRSK2	BR serine/threonine kinase 2
chr6:1653[illegible]	6	1,65E+08	1,65E+08	Hypomethy	13	-0,41523	-8,70176	0,003039	0,254897	1447	29	No	No	No	Yes	Exon	168090	6931	ENSG0000(	C6orf118	chromosome 6 open reading frame 118
chr11:721[illegible]	11	721758	722341	Hypomethy	11	-0,33928	-8,57403	0,003328	0,271197	584	27	No	Yes	No	No	Promoter	64787	0	ENSG0000(	EPS8L2	EPS8 like 2
chr14:100[illegible]	14	1,01E+08	1,01E+08	Hypomethy	11	-0,33723	-8,41203	0,003581	0,279652	620	31	Yes	Yes	No	No	Promoter	12490338[illegible]	39		LOC12490[illegible]	uncharacterized LOC124903382
chr11:463[illegible]	11	46366824	46367399	Hypomethy	12	-0,3797	-8,38163	0,00369	0,279652	576	20	Yes	Yes	No	No	Promoter	8525	0	ENSG0000(	DGKZ	diacylglycerol kinase zeta
chr7:4626[illegible]	7	462642	464190	Hypomethy	7	-0,22407	-8,37041	0,003726	0,279652	1549	83	Yes	No	No	No	Distal Inter	12490156[illegible]	16361		LOC12490[illegible]	uncharacterized LOC124901566
chr8:1434[illegible]	8	1,43E+08	1,43E+08	Hypomethy	10	-0,31839	-8,16948	0,004233	0,309518	989	44	Yes	Yes	No	No	Intron	10432605[illegible]	3015	ENSG0000(	MAFA-AS1	MAFA antisense RNA 1
chr19:179[illegible]	19	17981968	17983133	Hypomethy	12	-0,38854	-8,1273	0,00445	0,309518	1166	30	Yes	Yes	No	No	Exon	3780	30675	ENSG0000(	KCNN1	potassium calcium-activated channel subfamily N member 1
chr19:437[illegible]	19	43718955	43719453	Hypomethy	16	-0,49771	-8,09744	0,004486	0,309518	499	28	Yes	No	No	No	Promoter	56269	2879	ENSG0000(	IRGC	immunity related GTPase cinema
chr16:849[illegible]	16	84940886	84941842	Hypomethy	10	-0,30712	-8,03198	0,004703	0,309518	957	31	No	No	No	Yes	Downstrea[illegible]	10537137[illegible]	12533		LINC02176	long intergenic non-protein coding RNA 2176
chr2:2419[illegible]	2	2,42E+08	2,42E+08	Hypomethy	10	-0,31937	-8,00964	0,004739	0,309518	1123	38	No	No	No	Yes	Intron	10192728[illegible]	71431	ENSG0000(	LINC01237	long intergenic non-protein coding RNA 1237
chr18:794[illegible]	18	79411038	79412117	Hypomethy	16	-0,49765	-7,92805	0,005173	0,309518	1080	41	Yes	Yes	No	No	Exon	4772	15182	ENSG0000(	NFATC1	nuclear factor of activated T cells 1
chrX:7899[illegible]	X	789970	794133	Hypomethy	9	-0,27358	-7,92747	0,005209	0,309518	4164	53	Yes	Yes	Yes	No	Distal Inter	6473	159163	ENSG0000(	SHOX	SHOX homeobox
chr22:293[illegible]	22	29310878	29312428	Hypomethy	9	-0,29043	-7,87049	0,005318	0,309518	1551	42	Yes	Yes	No	No	3' UTR	10634	4228	ENSG0000(	GAS2L1	growth arrest specific 2 like 1
chr19:132[illegible]	19	13208528	13208963	Hypomethy	11	-0,35951	-7,86606	0,005354	0,309518	436	24	Yes	Yes	No	No	Exon	773	297516	ENSG0000(	CACNA1A	calcium voltage-gated channel subunit alpha1 A
chr19:350[illegible]	19	35059547	35059782	Hypomethy	17	-0,52395	-7,83578	0,005463	0,309518	236	14	Yes	Yes	No	No	Exon	10012867[illegible]	46493	ENSG0000(	HPN-AS1	HPN antisense RNA 1
[illegible]																					

Supplementary table 9. Details of the pyrosequencing analyses

PCR primers for pyrosequencing

**Name**		**Sequence (5’ to 3’, bio - biotinylated on 5’ end)**
CTRC1 (3’UTR)	F	GAA GGT AGT GAG AGT TGG ATA AA
	R	bio – TAC AAA CCC TCC CTA CAC TAA C
C8orf58/PDLIM2 (promoter)	F	bio – GTT TTT AGG GTT TTT TGT AAG AG
	R	CCA CCC CCT TAC TTA CCT
FSCN1 (distal intergenic region)	F	TTT TTT TGG GAA GGA GTT TGG
	R	bio – CCC CTA TAA TCC CAA CTA CTC A
MRPL36 (distal intergenic region)	F	GGA ATA TAT TGT ATA GGT TAA TTA TAA GT
	R	bio – TCC TAA TAA AAA AAA ACC CAA AAA AAC T

Sequencing primers for pyrosequencing

**Name**		**Sequence (5’ to 3’)**	**Chromosomal region targeted (hg38)**
CTRC1	F	GTG AGA GTT GGA TAA ATA G	chr19:18,777,661-18,777,792
C8orf58	F	CCC CCT TAC TTA CCT AA	chr8:22,589,551-22,589,731
FSCN1	F	GGA AGG AGT TTG GTT TT	chr7:5,611,146-5,611,178
MRPL36	F	AGT AAG TAG AAA TTA GAG TGT TG	chr5:1,779,081-1,779,213
